# Manganese(I)‐Catalyzed C−H Activation: The Key Role of a 7‐Membered Manganacycle in H‐Transfer and Reductive Elimination

**DOI:** 10.1002/anie.201606236

**Published:** 2016-09-07

**Authors:** Nasiru P. Yahaya, Kate M. Appleby, Magdalene Teh, Conrad Wagner, Erik Troschke, Joshua T. W. Bray, Simon B. Duckett, L. Anders Hammarback, Jonathan S. Ward, Jessica Milani, Natalie E. Pridmore, Adrian C. Whitwood, Jason M. Lynam, Ian J. S. Fairlamb

**Affiliations:** ^1^ Department of ChemistryUniversity of YorkYorkYO10 5DDUK

**Keywords:** catalysis, C−H activation, functionalization, manganese, sustainability

## Abstract

Manganese‐catalyzed C−H bond activation chemistry is emerging as a powerful and complementary method for molecular functionalization. A highly reactive seven‐membered Mn^I^ intermediate is detected and characterized that is effective for H‐transfer or reductive elimination to deliver alkenylated or pyridinium products, respectively. The two pathways are determined at Mn^I^ by judicious choice of an electron‐deficient 2‐pyrone substrate containing a 2‐pyridyl directing group, which undergoes regioselective C−H bond activation, serving as a valuable system for probing the mechanistic features of Mn C−H bond activation chemistry.

C−H bond activation–functionalization chemistry is a central arena for catalyst development and synthetic application.^1^ Transition metals mediate the efficient and selective activation of C−H bonds, with recent attention focusing on environmentally benign and sustainable metals, for example, Mn, Co, Fe, and Cu.^2^ Mn^I^ promotes C−H activation of substrates containing nitrogen‐directing groups.^3^ For example, **1** gives cyclomanganated complex **2**, with subsequent reaction with alkyne **3** forming a proposed 7‐membered ring intermediate **4** (Scheme 1).^4^ Formation of either **5**, **6**, or **7** results from reductive elimination, H‐transfer, or dehydrogenative annulation, respectively.

**Scheme 1:**
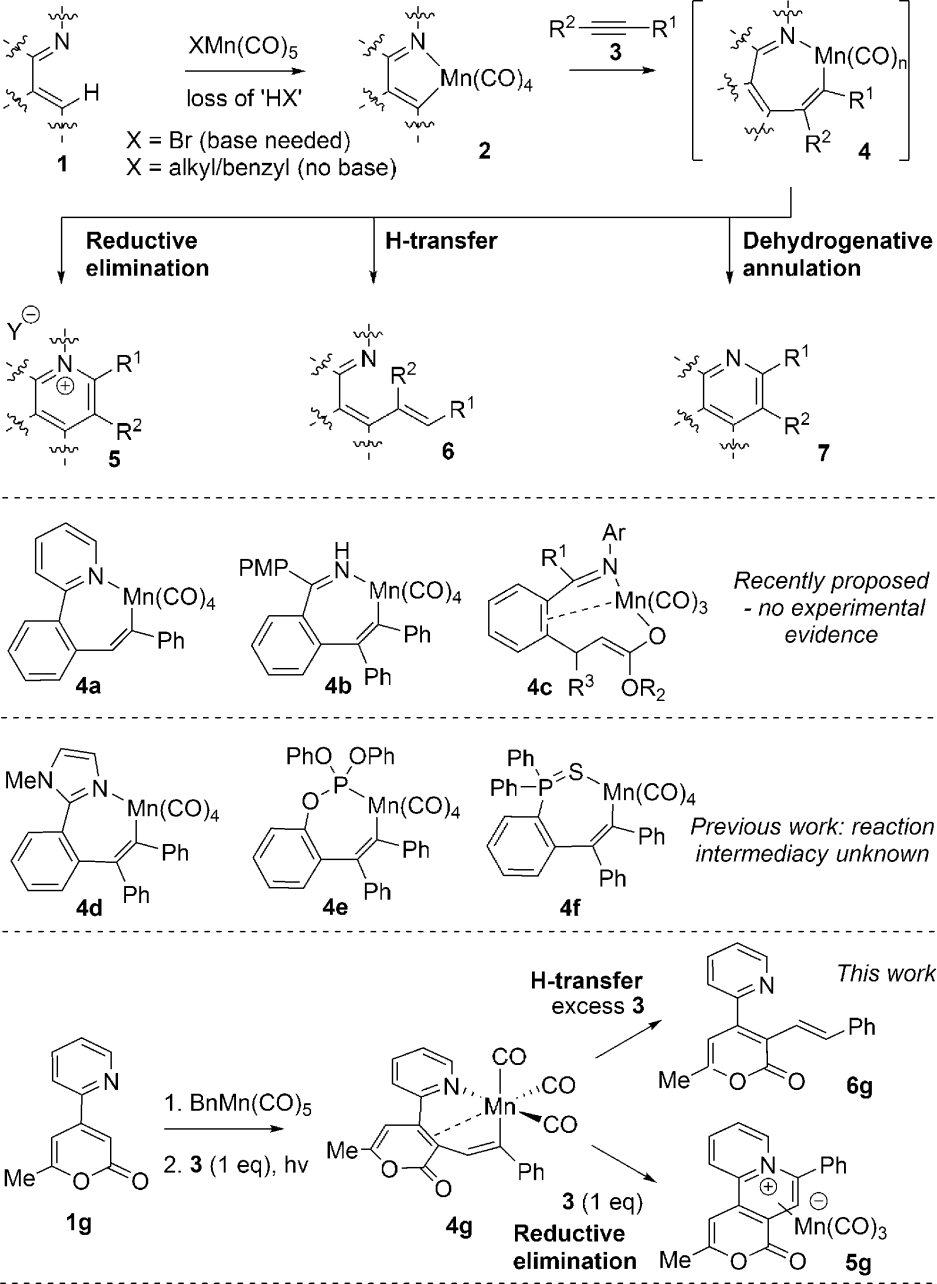
Manganese(I)‐catalyzed C−H activation, and potential products and intermediates.

Processes utilizing Mn^I^, particularly [Mn(C^N)(CO)_4_] **2**,^5^, ^6^ have been of broad interest. The mechanistic features of the remarkable synthetic work of Ackermann and Wang,^3^, ^4^ where intermediates **4 a**–**c** have been proposed, prompted us to examine whether they could be detected and characterized and then subsequently be shown to deliver organic products such as **5**–**7**. Complexes **4 d**–**f**, formed by insertion of internal alkynes are known,^6^, ^7^ but their competence in terms of a fully connected reaction system, affording organic products, has not been examined. As 18‐electron species containing four CO ligands, possessing high thermodynamic stability, they are unlikely to be directly involved in the catalytic cycle.^8^

Herein we describe a suitable reaction system (**1 g**→**4 g**→**5 g** or **6 g**, Scheme 1) that takes advantage of the exquisite reactivity of an electron‐deficient 2‐pyrone ring system containing a 2‐pyridyl directing group (**1 g**). We recognized that the 2‐pyrone could act as a hemilabile ligand in 7‐membered manganacycle **4 g**, potentially providing sufficient stabilisation for observation of this key intermediate. Our findings demonstrate that **4 g** acts as a central manifold to reductive elimination and H‐transfer, giving products **5 g** and **6 g**, respectively, with details described herein.

Our study began with the reaction of 2‐pyrone **1 g** with BnMn(CO)_5_ in hexane at 75 °C, which gave cyclometalated **2 g** cleanly and in quantitative yield (Scheme 2). Complex **2 g** was fully characterized (see the Supporting Information); a single crystal X‐ray structure confirmed that regioselective C−H activation occurred at C3, in keeping with Pd^II^‐direct arylations of 2‐pyrones,^9^ albeit most likely by a σ‐CAM‐type process.^10^

**Scheme 2:**
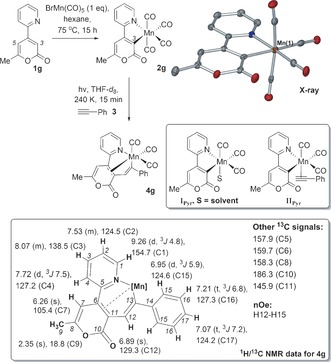
Cyclomanganation of **1 g** gives **2 g**, which upon photolysis with phenylacetylene **3** gives **4 g**. The X‐ray structure of **2 g** is given (top right, ellipsoids set at 50 % probability; H‐atoms omitted and Mn atom labeled only for clarity). Insets: proposed transient intermediates on route to **4 g** and the key NMR data for **4 g**.

We hypothesized that UV irradiation^11^ of **2 g** would lead to solvated intermediate **I_Pyr_** (Scheme 2, middle inset).^12^ Subsequent alkyne trapping via intermediate **II_Pyr_**, would then convert into the alkyne insertion manganacycle **4 g**. UV irradiation (Hg/Xe Arc lamp, 200–2500 nm) of a mixture of **2 g** and **3** (1.1 equiv) in [D_8_]THF at 240 K (at 5 min intervals), and reaction monitoring by ^1^H NMR spectroscopy between intervals, revealed the formation of a new intermediate that grows up to 9.6 % conversion. Further irradiation resulted in spectral broadening (paramagnetic species), but crucially, full NMR analysis of manganacycle **4 g** was possible, with HMQC/ HMBC correlation methods/n.O.e. experiments. Analysis shows that **4 g** formed regioselectively at C3 (Scheme 2, bottom inset). MS analysis also confirmed the presence of **4 g** (LIFDI *m*/*z* 427 for [M]^+.^ and ESI *m*/*z* 428 for [MH]^+^) in solution.

Experimentally there is evidence in **4 g** of an interaction between the 2‐pyrone olefinic bond (C6‐C11) and the Mn^I^ center at *δ*=159.7 ppm (C6) and *δ*=145.9 ppm (C11), which stabilizes the tricarbonyl complex. Computational studies (DFT methods) confirm that HOMO−4 within **4 g** has 2‐pyrone‐Mn bonding character (see the Supporting Information), confirming **4 g** as a feasible structure. The small coordination shifts in the ^13^C{^1^H} NMR spectrum imply this interaction is weak, although generation of a vacant site at Mn (**4 g′**) and subsequent alkyne coordination (**4 g′′**) ought to be feasible. The DFT studies for **III_Pyr_** (**4 g**) and **III_Ph_** (**4 a**) indicate no low‐lying vacant orbitals (HOMO–LUMO gap=1.70154–1.97588 eV), consistent with Mn having an 18‐electron count.

Warming of the [D_8_]THF solution of **4 g** to room temperature led to the formation of the reductive elimination product **5 g** (Scheme 3). Complex **5 g** was fully characterized (see the Supporting Information) and confirmed by X‐ray analysis to possess a Mn(CO)_3_ anion. **5 g** was also formed in 87 % yield on treatment of **2 g** with **3** (1.1 equiv.) at 80 °C, Et_2_O, 18 h (sealed tube). Thus, the same reaction pathway (**2 g**+**3**→**5 g**) results from either UV irradiation or thermal heating, validating our approach in utilizing UV irradiation to enable detection and characterization of intermediate **4 g**.

**Scheme 3:**
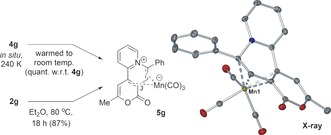
Thermally controlled reductive elimination from either **2 g** or **4 g** to give **5 g**. An X‐ray structure of a single crystal of **5 g** is also shown (ellipsoids set to 50 % probability; H‐atoms omitted and Mn atom labeled only, for clarity).

Interestingly, catalytic reactions of **1 g** with **3**, under the reaction conditions reported by Wang et al.^4^ for 2‐phenylpyridine **1 a** (conditions: BrMn(CO)_5_, Cy_2_NH, Et_2_O, 100 °C for 6–24 h), do not lead to formation of alkenylated products (for example, **6 g**). This indicates that the rate of reductive elimination from **4 g** to give **5 g** is faster than the rate for alkyne H‐transfer to give **6 g** (see above). We rationalized that reaction of **2 g** in neat phenylacetylene **3** would enable H‐transfer to become the dominant pathway (Scheme 4), but the reaction afforded three new products. Firstly, the H‐transfer product **6 g** was formed in 28 % yield; an excess of **3** favors H‐transfer over reductive elimination. Central to the success of the reaction is coordination of a second molecule of alkyne **3** and subsequent alkyne H‐transfer of intermediate **4 g**. The other products **8** and **9** were unexpected, resulting from a noteworthy Diels–Alder reaction (DAR) of **3** with the 2‐pyridine ring,^13^ followed by ring fragmentation (single‐crystal X‐ray structures of **8** and **9** confirmed the molecular connectivity, correlating with NMR spectroscopy, see the Supporting Information). Compound **9** shows that the 2‐pyrone participated in a secondary inverse electron demand DAR.^14^ Along with **6 g**, both **8** and **9** derive from **4 g**, where the DARs and 2‐pyridyl fragmentation are secondary reactions.

**Scheme 4:**
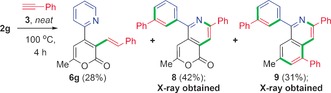
Reaction of **2 g** in neat phenylacetylene **3**. The green bonds show the newly formed bonds in the organic products, with red showing the insertion location of **3** (**5 g** not observed under these reaction conditions).

To understand the steps leading to the formation of **5 g** DFT methods were used (Scheme 5, see the Supporting Information for details of DFT calculations). Starting from **II_Pyr_**, formed via loss of CO from **2 g** and coordination of **3**, insertion of coordinated alkyne into the Mn−C(pyrone) bond proceeds through a low‐energy transition state (**TS_IIPyr‐IIIPyr_**) to give **III_Pyr_**. The latter intermediate is equivalent to characterized **4 g**. C−N reductive elimination from **III_Pyr_**, via transition state **TS_IIIPyr‐5g_**_‐**iso**_, results in the formation of the 2‐methyl‐4‐oxo‐6‐phenyl‐4*H*‐3,7λ^5^‐pyrano[4,3‐a]quinolizin‐7‐ylium ring system (**5 g**). A DRC analysis of **TS_IIIPyr‐5g_**_‐**iso**_ revealed that the imaginary eigenvector led to **5 g**‐**iso** (the coordination isomer of **5 g**); a π‐slip then gives **5 g**.

**Scheme 5:**
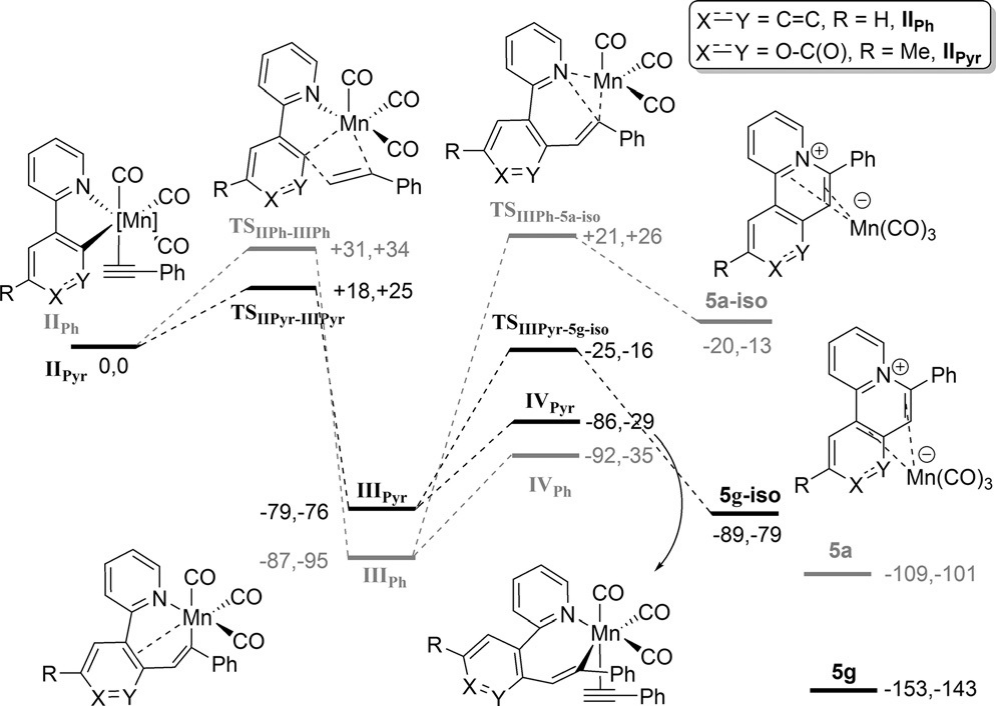
DFT calculations showing the feasibility of reductive elimination from **5 a** and **5 g**, starting from intermediates **II_Ph_** and **II_Pyr_**, respectively. Energies are zero point energy‐corrected electronic energies and Gibbs energies at 298.15 K in kJ mol^−1^ relative to **II**.

The corresponding potential energy surface for the phenyl‐substituted system (giving the Chen and Wang product **5 a**) revealed that the same reaction pathway was viable (pathway shown in gray in Scheme 5). The barrier to insertion of **3** (**TS_II‐III_**) was slightly greater (Gibbs energies at 298.15 K relative to the respective compound **II** +25 kJ mol^−1^ for 2‐pyrone versus +34 kJ mol^−1^ for phenyl) and that **III_Pyr_** was relatively higher in energy than **III_Ph_** (−76 kJ mol^−1^ versus −95 kJ mol^−1^). To explain the different outcome from the phenyl and 2‐pyrone substituents it is informative to consider the higher energy of **TS_IIIPh‐5a_**_‐**iso**_ (+26 kJ mol^−1^) against **TS_IIIPyr‐5g_**_‐**iso**_ (−16 kJ mol^−1^). Therefore, the energetic spans for reductive elimination are 60 kJ mol^−1^ (2‐pyrone) and 121 kJ mol^−1^ (phenyl). When compared with the formation of **IV_Pyr_** and **IV_Ph_**, which is the next step in forming H‐transfer products **5 g** and **5 a**, respectively, it is evident that the reductive elimination to form **5 g** is competitive, but in the case of **5 a** the much larger energetic span to reductive elimination allows for productive catalysis via alkyne coordination to give **IV_Ph_**.^4^

While no double alkyne insertion products were detected in reactions of **2 g** with phenylacetylene **3**, the reaction of related derivative **2 h** with **3** resulted in exclusive formation of double alkyne insertion product **10** (Scheme 6; the structure of **6 h** is shown as an expected alkenylated product). This remarkable result shows the impact that a subtle change to the pyridyl directing group has on the barriers to these steps.

**Scheme 6:**
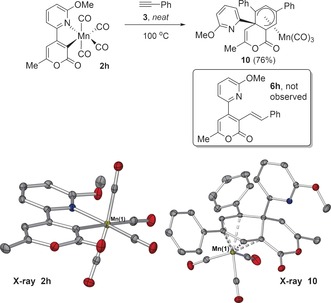
Double alkyne insertion into **2 h**. Dotted lines show Mn coordination in complex **10** for clarity (ellipsoids set to 50 % probability; H‐atoms omitted and Mn atom labeled only, for clarity).

We rationalized the experimental observations by DFT calculations, which enabled a mechanism for this reaction and the differences between the phenyl‐ and 2‐pyrone‐substituted complexes to be proposed (Scheme 7). In the case of the pyrone derivative, coordination of alkyne to **III_Pyr_** results in formation of **IV_Pyr_** having two energetically accessible fates. H‐transfer through **TS_IVPyr‐VPyr_** (+3 kJ mol^−1^) results in the formation of alkynyl complex **V_Pyr_** which would liberate **6 h**, however insertion of the alkyne into the Mn−C bond of **IV_Pyr_** through **TS_IVPyr‐VIPyr_** (+4 kJ mol^−1^) affords more energetically favourable **VI_Pyr_**. The process seen for reactions of **2 h** has resulted in the formation of two C−C bonds.Preliminary investigations indicate that this proceeds through a “two‐steps no intermediate” pathway^15^ with the initial insertion into the Mn−C bond, followed by cyclization giving a six‐membered ring without an intermediate. However, in **VI_Pyr_** the Mn is η^3^‐coordinated to the pendant pyridyl group and newly formed ring. To form **10_Pyr_**, which is the lowest point on the potential energy surface at −320 kJ mol^−1^, the Mn needs to migrate to the alternative ring‐face. We postulate that this involves migration onto one of the phenyl rings in the ligand, for example, **VIIa_Pyr_**. The ring rotates allowing the Mn to migrate to the other face of the pentadienyl system, giving **VIIb_Pyr_**. It is reasonable to presume that this proceeds via a low energy ring‐walking process.^16^

**Scheme 7:**
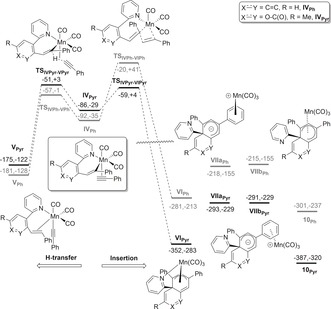
DFT calculations showing the feasibility of a double alkyne insertion pathway to rationalize formation of double alkyne insertion product **10**. Energies are zero point energy‐corrected electronic energies and Gibbs energies at 298.15 K in kJ mol^−1^ relative to **II**.^17^

In the case of the phenyl derivative, all of the states predicted for the 2‐pyrone system are viable; however, **TS_IVPh‐VIPh_** is far higher in energy than **TS_IVPh‐VPh_** (+41 kJ mol^−1^ versus −1 kJ mol^−1^). Therefore, insertion of the second alkyne is non‐competitive, with the H‐transfer pathway leading to the alkenylated product, consistent with experimental observations.

In conclusion, we have detected and characterized a commonly proposed 7‐membered manganacycle **4 g** (of direct relevance to generic structure **4**, Scheme 1). Manganacycle **4 g** sits at the selectivity junction to reductive elimination or H‐transfer steps. Depending on the reaction conditions, **5 g** or **6 g** products form that correspond to reductive elimination and protonation pathways, respectively. Double alkyne insertion to give **10** has also been revealed in these studies. Our observations provide the first clear cut evidence that manganacycles such as **4** are key intermediates in Mn^I^‐mediated C−H bond activation processes involving substrates containing directing groups.^3^, ^4^, ^7^ More generally, such intermediates may be considered as leading to side reactions, but here we have shown that it presents an opportunity to control product selectivity. Serendipitously we have uncovered a rare example of a DAR of a pyridine derivative, where the intermediate fragments to form products such as **8** and **9**. Taken together, our findings provide a unique insight into Mn^I^‐mediated C−H bond activation processes, especially how relatively minor changes in substrate structure influence product selection; Mn^I^‐based metallocycles clearly offer rich chemistry,^3^ much potential, and warrant further study more generally in organic and organometallic chemistry.

*Dedicated to Professors Michael Bruce and Robin N. Perutz*

## Supporting information

As a service to our authors and readers, this journal provides supporting information supplied by the authors. Such materials are peer reviewed and may be re‐organized for online delivery, but are not copy‐edited or typeset. Technical support issues arising from supporting information (other than missing files) should be addressed to the authors.

Supplementary
