# Supplementary material for: Manganese(I)‐Catalyzed C−H Activation: The Key Role of a 7‐Membered Manganacycle in H‐Transfer and Reductive Elimination
Source: Angew Chem Int Ed Engl. 2016 Sep 7;55(40):12455–9. doi: 10.1002/anie.201606236 (PMC5113680; doi:10.1002/anie.201606236)
Supplement: Supplementary file 1 — Supplementary [file ANIE-55-12455-s001.pdf]

Angewandte Chemie
Eine Zeitschrift der Gesellschaft Deutscher Chemiker

Supporting Information

Manganese(I)-Catalyzed C−H Activation: The Key Role of a 7-Membered Manganacycle in H-Transfer and Reductive Elimination

Nasiru P. Yahaya, Kate M. Appleby, Magdalene Teh, Conrad Wagner, Erik Troschke, Joshua T. W. Bray, Simon B. Duckett, L. Anders Hammarback, Jonathan S. Ward, Jessica Milani, Natalie E. Pridmore, Adrian C. Whitwood, Jason M. Lynam, and Ian J. S. Fairlamb**

anie_201606236_sm_miscellaneous_information.pdf

Supporting Information

Contents

1. Synthetic procedures and characterization data 2
1.1. General details........ 2
1.2. Synthesis and characterization of manganacycle (4g) 3
1.3. Synthesis of 4-(2'-pyridyl)-2-pyrone (1g) 6
1.4. Synthesis of 4-(6'-methoxy-2'-pyridyl)-2-pyrone (1h)........ 7
1.5. Synthesis of manganese complex (2g) 8
1.6. Synthesis of manganese complex (2h) 8
1.7. Synthesis of complex (5g) formed by alkene insertion and reductive elimination 9
1.8. H-transfer reactions of manganacycle 2g in neat phenylacetylene (3) 9
1.9. Synthesis of η^4-tricarbonylmanganese(I) complex (10) 11

2. Representative NMR spectra of compounds 12

3. X-ray structure details 22
3.1. X-ray structure for complex 2g........ 22
3.2. X-ray structure for complex 2h........ 23
3.3. X-ray structure for complex 5g........ 24
3.4. X-ray structure for compound 8........ 25
3.5. X-ray structure for compound 9........ 26
3.6. X-ray structure for complex 10 27
4. Computational studies (using DFT methods)........ 28
4.1. Computational details (general) 28
4.2. Collated theoretical data for the 2-pyrone system 29
4.3. Collated theoretical data for the 2-phenylpyridine system 30
4.4. Output files 31
5. References........ 89

1. **Synthetic procedures and characterization data**

1.1. General details

Solvents and reagents. All commercially-sourced reagents were purchased and used as received, unless otherwise noted, from Alfa Aesar, Acros Organics, Sigma-Aldrich, or Fluorochem. Dry solvents used were obtained from a Pure Solv MD-7 solvent machine and stored under nitrogen. Anhydrous di-n-butyl ether was used as received (<H_2O 50 ppm). All reactions requiring anhydrous or air-free conditions were carried out in dry solvents, under an argon or nitrogen atmosphere, on a high vacuum line (~0.1 mmHg), using Schlenk techniques.

Nuclear magnetic resonance spectroscopy. Proton (^1H) and Carbon-13 (^{13}C decoupled ^1H) were recorded on a Jeol ECX400 or Jeol ECS400 spectrometer at 400 and 100 MHz respectively, or on a Bruker AV500 operating at 500 and 125 MHz respectively. Chemical shifts are reported in parts per million (ppm) of Multiplicities are described as singlet (s), doublet (d), triplet (t), quartet (q), pentet (p), multiplet (m), apparent (app.) and broad (br). Coupling constants (J) are quoted to the nearest 0.1 Hz. Spectra were processed using MestreNova; apodization (Sine Bell) was used to aid enhancement of the J couplings, where necessary, for processing. Spectra were exported directly from MNova (as .emf or .tiff image files), embedded directly into this document. Representative ^1H and ^{13}C NMR spectra for all novel compounds are provided. For the NMR-photoirradiation studies: A LOT Hg/Xe Arc lamp, model LSE 139, with an output of 200-2500 nm, was used with a LOT LSN 271 power supply to irradiate samples. Samples were irradiated in an NMR probe, with a liquid nitrogen cooling system, and a EUROTHERM B-VT 2000 variable temperature unit.

Chromatography. Thin layer chromatography (TLC) was carried out using Merck aluminium backed 5554 plates. Spots were visualized by quenching of ultraviolet light (λ_{max} = 254 nm) and then stained and heated with one of the following treatments – anisaldehyde, potassium permanganate or phosphomolybdic acid, as appropriate. Flash column chromatography was ordinarily performed using Merck 60 silica gel. Preparatory TLC was carried out using Analtech UNIPLATE glass-backed silica plates.

Melting points. Melting points were determined using a Stuart SMP3 melting point apparatus using a slow temperature ramp of 3 °C min^{-1}.

Elemental analysis. Elemental analysis was carried out using an Exeter Analytical CE-440 Elemental Analyser, with the percentages reported as an average of two runs.

X-Ray crystallography. Diffraction data were collected at 110 K on an Agilent SuperNova diffractometer with MoKα radiation (λ = 0.7107 Å). Data collection, unit cell determination and frame integration were carried out with CrysalisPro. Absorption corrections were applied using face indexing and the ABSPACK absorption correction software within CrysalisPro. Structures were solved and refined using Olex2252 implementing SHELX algorithms and the Superflip253-255 structure solution

program. Structures were solved by charge flipping, Patterson or direct methods and refined with the ShelXL256 package using full-matrix least squares minimisation. All non-hydrogen atoms were refined anisotropically and structures presented were processed using X-seed software.

1.2. Synthesis and characterization of manganacycle (4g)

The manganese complex **2g** (10 mg, 0.283 µmol) and phenylacetylene (3.42 µL, 0.311 µmol) were dissolved in dry THF-d_8 (0.5 mL), under N_2, and injected into a Young's NMR tube. The tube was cooled to 243 K and probed using an Avance III 500 MHz Bruker NMR spectrometer, also at 243 K. A Dewar flask containing acetone cooled with dry ice was used to transport the NMR tube between the lamp and the spectrometer. A ^{1}H NMR spectrum was collected, which showed no reaction. The sample was irradiated with UV light at 240 K in 5 minute intervals, with ^{1}H NMR spectra collected after each exposure. NMR signals corresponding to the intermediate species (**4g**) grew only after UV exposure – the 1H resonances are indicated by a star (*) in the figure below. The experiment (filename: kma-3-53, HMQC-^{13}C cnst2 = 145 Hz) was stopped after 15 mins exposure to prevent the formation of paramagnetic species.

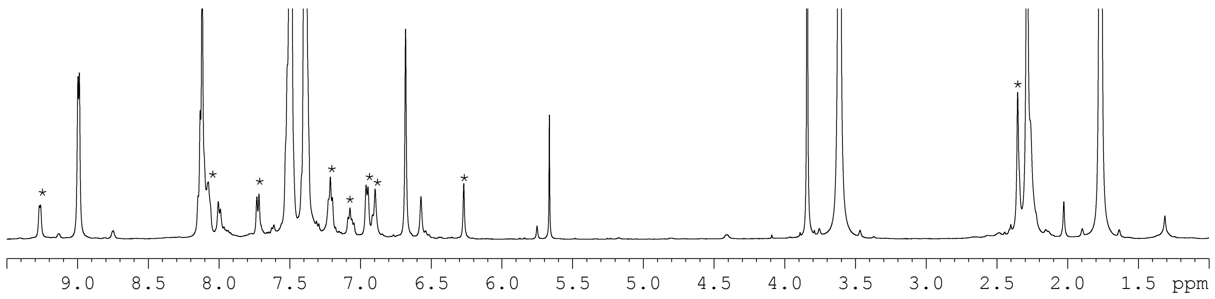

Figure 1 ^{1}H NMR spectrum showing formation of manganacycle 4g.

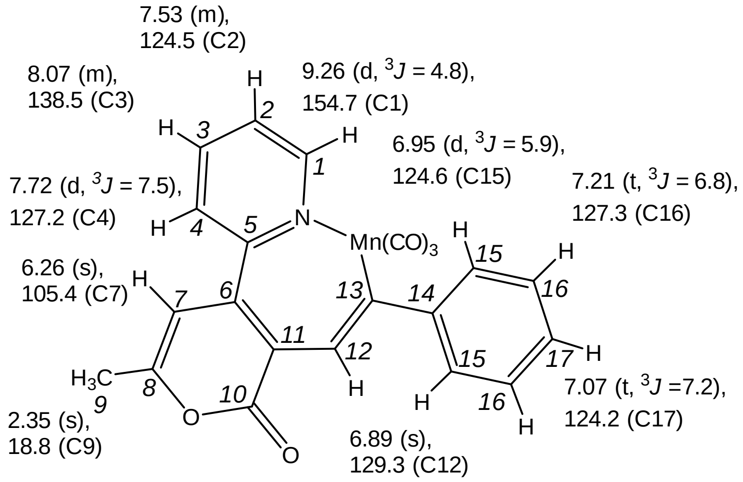

Other ^{13}C signals:
157.9 (C5)
159.7 (C6)
158.3 (C8)
186.3 (C10)
145.9 (C11)

nOe:
H12-H15

Figure 2 Summary of ^{1}H and ^{13}C NMR data for manganacycle 4g.

Collated NMR spectroscopic data for 4g: ^{1}H NMR (500 MHz, d^8-THF): 9.26 (d. J_{HH} = 4.8, 1H, H-1), 8.07 (m, 1H, H-3), 7.72 (d, J_{HH} = 7.5, 1H, H-4), 7.53 (m, 1H, H-2), 7.21 (*p*-t, J_{HH} = 6.8, 2H, H-16), 7.07 (*p*-t, J_{HH} = 7.2, 1H, H-17), 6.95 (d, J_{HH} = 5.9, 2H, H-15), 6.89 (s, 1H, H-12), 6.26 (s, 1H, H-7), 2.35 (s, 3H, H-9). ^{13}C NMR (500 MHz, d^8-THF): 186.3 (C-10), 159.7 (C-6), 159.0 (C-14), 158.3 (C-8), 157.9 (C-5), 154.7 (C-1), 145.9 (C-11), 138.5 (C-3), 129.3 (C-12), 127.3 (C-16), 127.2 (C-4), 124.6 (C-15), 124.5 (C-2), 124.2 (C-17), 105.4 (C-7), 119.2 (C-13), 18.8 (C-9).

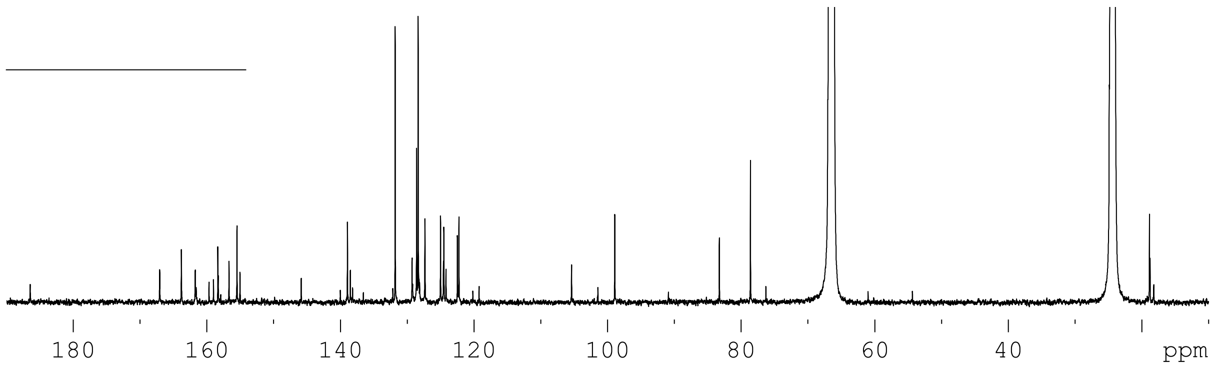

Figure 3 ^{13}C NMR spectrum of reaction mixture containing 4g.

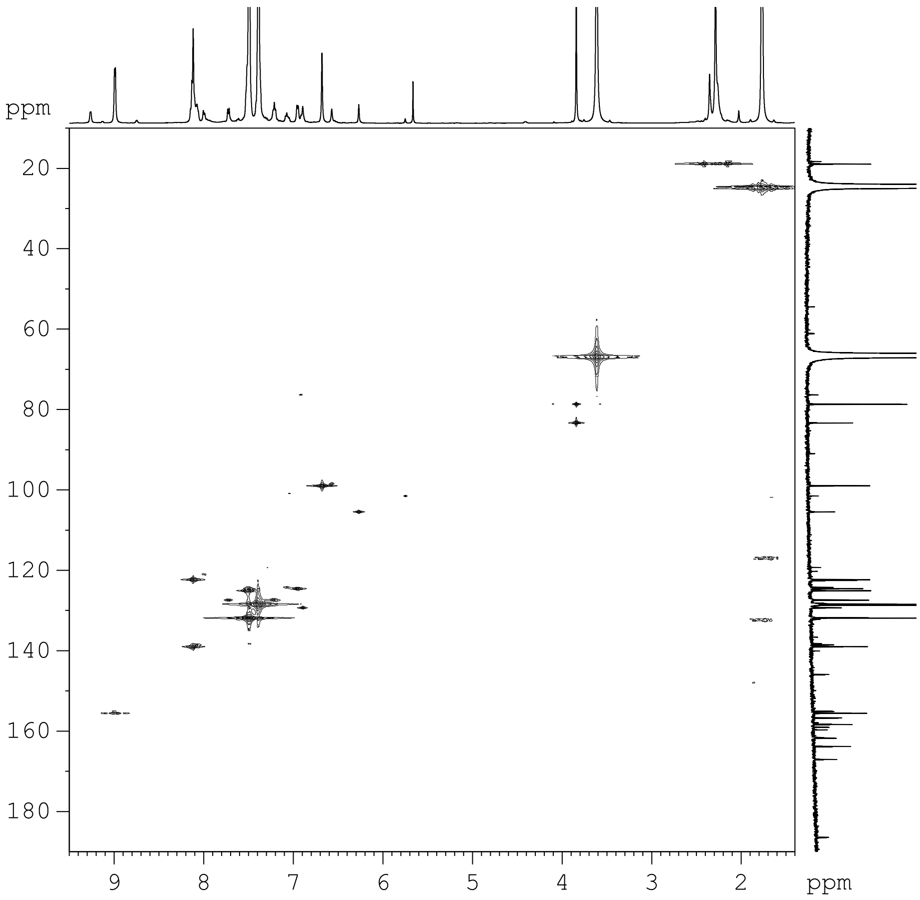

Figure 4 HMQC spectrum of reaction mixture containing 4g (HMQC-^{13}C cnst2 = 12 Hz).

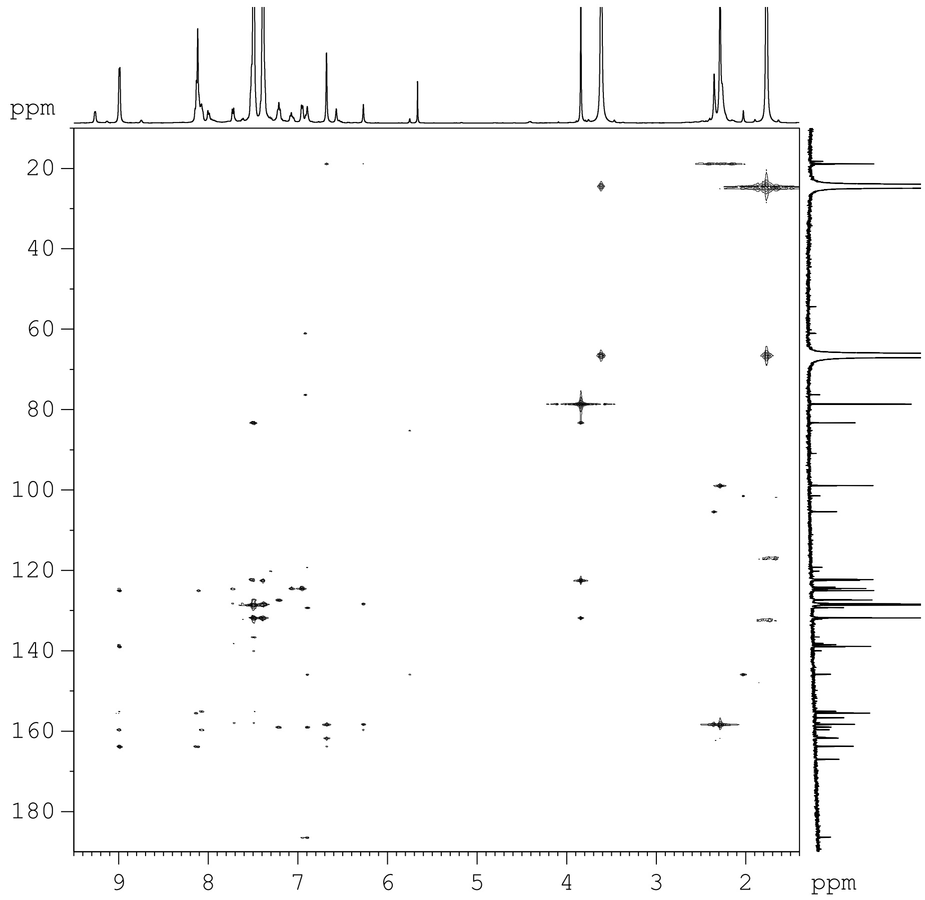

Figure 5 HMBC spectrum of reaction mixture containing 4g.

1.3. Synthesis of 4-(2'-pyridyl)-2-pyrone (1g)

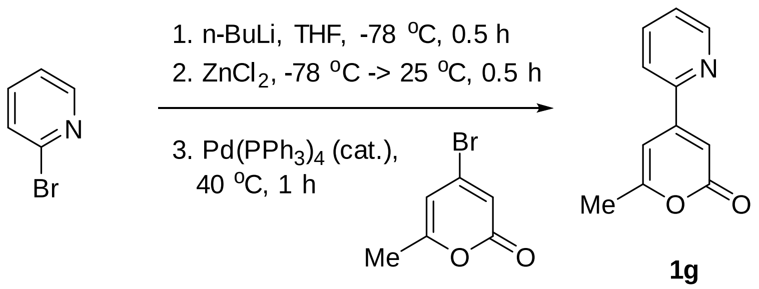

To a flame-dried Schlenk tube under N_2, equipped with a magnetic stirrer bar, was added 2-bromopyridine (2.78 mmol, 440 mg, 1.05 eq.) in dry THF (30 mL). The solution was cooled to -110 °C and then *n*-BuLi (2.78 mmol, 1.2 mL, 1.05 eq.) was added dropwise over 10 min with stirring, which was left to continue stirring for a further 30 min. To a separate, flame-dried Schlenk tube under N_2, equipped with a magnetic stirrer bar, was added high vacuum line-dried $ZnCl_2$ (2.91 mmol, 400 mg, 1.1 eq.; dried to constant weight, *ca.* 12 h at 0.1 mmHg). The lithiated 2-pyridine, generated *in situ*, was transferred *via* cannula at -110 °C to the $ZnCl_2$, over 5 mins. The mixture was allowed to warm to -40 °C, with constant stirring for 30 min. To a separate flame-dried Schlenk tube under N_2, equipped with a magnetic stirrer bar, was added

4-bromo-6-methyl-2-pyrone (2.65 mmol, 500 mg, 1 eq.), $Pd(PPh_3)_4$ (0.13 mmol, 150 mg, 5 mol%) sequentially and dry THF (20 mL). The zincated 2-pyridine was rapidly transferred *via* cannula and the reaction mixture allowed to stir at 22 °C for 12 h. The reaction was monitored by TLC analysis. Upon completion, the reaction was quenched with saturated NH_4Cl (ca. 30 mL), and the mixture filtered through Celite™. The mixture was extracted with EtOAc (2x25 mL). The combined organic extracts were dried ($MgSO_4$), filtered and concentrated *in vacuo*. The crude product was purified by silica gel column chromatography (petroleum ether:EtOAc, 60:40, *v/v*) to afford the product as a creamy solid (485 mg, 98 %). MP 89-90 °C. ^1H NMR (400 MHz, CD_2Cl_2) δ 8.70–8.71 (m, 1H), 7.82 (td, J_{HH} = 7.7, 1.5, 1H), 7.76 (d, J = 7.7, 1H), 7.38 (ddd, J_{HH} = 7.7, 4.8, 1.5, 1H), 6.81 (s, 1H), 6.68 (s, 1H), 2.32 (s, 3H). ^{13}C NMR (101 MHz, CD_2Cl_2) δ 163.8, 162.9, 153.9, 152.81, 150.5, 137.6, 125.4, 121.9, 109.3, 102.5, 20.6. IR (solid-state ATR, cm^{-1}) 3073, 2952, 1702, 1632, 1551, 1432, 785, 842, 874. MS; HRMS (ESI^+) m/z: $[MH]^+$ calcd for $C_{11}H_9NO_2$ 188.0633; Found 188.0685.

1.4. Synthesis of 4-(6'-methoxy-2'-pyridyl)-2-pyrone (1h)

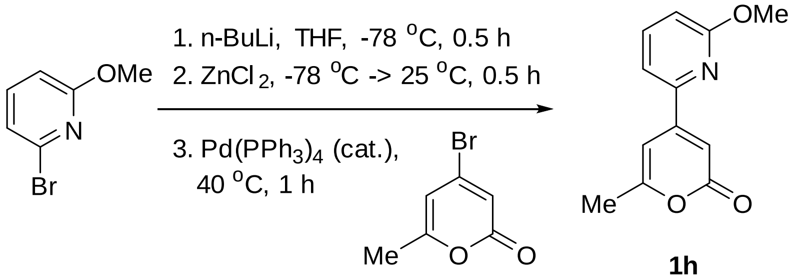

1h

Following a similar procedure to the synthesis of **1g**: 4-bromo-6-methyl-2-pyrone (500 mg, 2.65 mmol) was reacted with 2-bromo-6-methoxypyridine (547 mg, 2.91 mmol) to afford the title compound as a creamy solid (496 mg, 86% yield). MP 80-82 °C. ^1H NMR (400 MHz, CD_2Cl_2) δ 7.67 (dd, J_{HH} = 8.3, 7.3, 1H), 7.32 (d, J_{HH} = 7.3, 1H), 6.84(d, J_{HH} = 8.3, 1H), 6.82 (s, 1H), 6.70 (s, 1H), 4.00 (s, 3H), 2.34 (s, 3H). ^{13}C NMR (101 MHz, CD_2Cl_2) δ 164.0, 162.1, 153.2, 149.6, 139.4, 114.6, 113.2, 108.9, 102.1, 53.6, 20.4; 1 carbon signal not observed. IR (solid-state ATR cm^{-1}) 3110, 2080, 2075, 1750, 1700, 1680, 1550, 1480, 1330, 1291, 1030, 988, 830, 805. MS; HRMS (ESI^+) m/z: $[MH]^+$ calcd for $C_{12}H_{12}NO_3$ 218.0739; Found 218.0772.

1.5. Synthesis of manganese complex (2g)

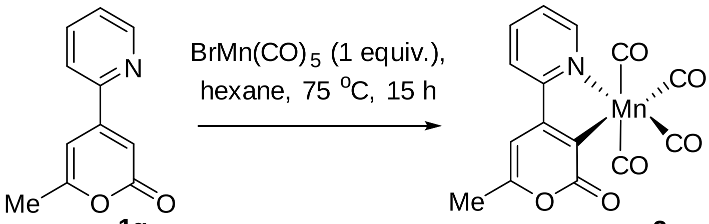

A mixture of **1g** (500 mg, 2.67 mmol, 1 eq.), benzylmanganese(I) pentacarbonyl (764 mg, 2.67 mmol, 1 eq.) in dry deoxygenated hexane (20 mL) was stirred at 75 °C under nitrogen for 18 h. The reaction was allowed to cool to ambient temperature and filtered through Celite™. The solvent was removed *in vacuo* to afford the title compound as a bright yellow crystalline solid (943 mg, quant.). MP 155–156 °C. ^{1}H NMR (400 MHz, DMSO-d_6) δ 8.89 (br s, 1H), 8.15 (br s, 2H), 7.54 (br s, 1H), 6.84 (br s, 1H), 2.26 (s, 3H). ^{13}C NMR (101 MHz, DMSO-d_6) δ 217.7 (Mn-CO), 213.6 (Mn-CO), 212.5 (Mn-CO), 167.6, 162.7, 159.2, 158.3, 157.5, 155.5, 139.5, 125.8, 122.8, 99.9, 19.2. IR (solid-state ATR cm^{-1}) 2081, 1966, 1927, 1681, 1633, 1596, 1259, 1015, 783. MS; HRMS (ESI^{+}) m/z: [MH]$^{+}$ calcd for $C_{15}H_{9}MnNO_{6}$ 353.9766; Found 353.9769.

1.6. Synthesis of manganese complex (2h)

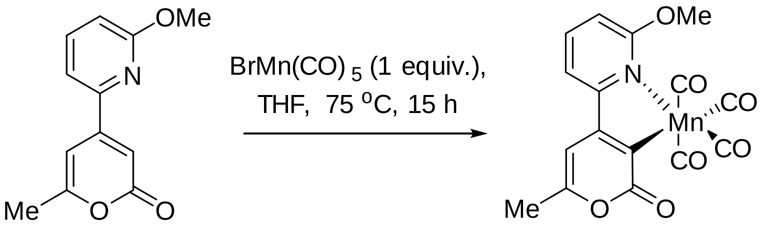

Following a similar procedure to the synthesis of **2g** (note change of solvent): MnBn(CO)$_5$ (395 mg, 1.38 mmol,) was reacted with **1h** (300 mg, 1.38 mmol) in THF (15 mL) to afford the desired product as a sticky yellow solid (520 mg, 98% yield). MP 159–160 °C. ^{1}H NMR (400 MHz, CD_2Cl_2) δ 7.87 (t, J_{HH} = 8.0, 1H), 7.34 (d, J_{HH} = 7.5, 1H), 6.76 (d, J_{HH} = 8.0, 1H), 6.22 (s, 1H), 4.08 (s, 3H), 2.27 (s, 3H). ^{13}C NMR (176 MHz, CD_2Cl_2) δ 218.9 (Mn-CO), 214.8 (Mn-CO), 212.2 (Mn-CO), 169.1, 166.9, 165.1, 163.6, 157.9, 157.0, 141.1, 114.6, 105.1, 99.9, 56.3, 19.9. IR (solid-state ATR cm^{-1}) 3084, 3020, 2957, 2923, 2853, 2073, 1993, 1946, 1685, 1633, 1473, 1597, 1569, 1509, 1425, 1368, 1318, 1286, 1260, 1093, 1049, 1016, 929, 883, 799. MS; HRMS (ESI^{+}) m/z: [MH]$^{+}$ calcd for $C_{16}H_{11}MnNO_{7}$ 382.9838; Found 382.9872.

1.7. Synthesis of complex (5g) formed by alkene insertion and reductive elimination

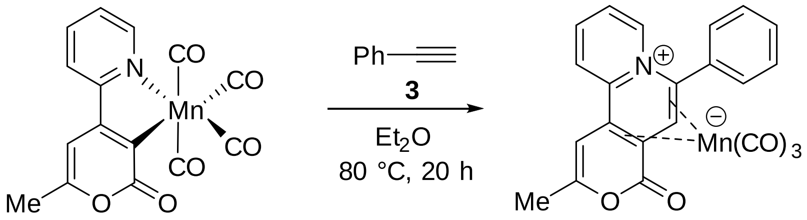

2g **5g**

Complex **2g** (200 mg, 0.57 mmol) and phenyl acetylene (70 mg, 0.68 mmol) in dry deoxygenated diethyl ether (30 mL) was stirred at 80 °C for 18 h. The mixture was allowed to cool to ambient temperature. A precipitate formed, thus CH_2Cl_2 (10 mL) was added to aid solubilisation of all the solids. The solution was filtered through a short pad of silica gel, washed with small portions of ethyl acetate (60 mL). The filtrate was concentrated *in vacuo* and the crude residue purified by flash column chromatography on silica gel (EtOAc/40-60 °C petroleum ether, 3/2 *v/v*) to afford the title compound as a yellow powder (210 mg, 87 %). MP 89-90 °C. ^{1}H NMR (400 MHz, CD_2Cl_2) δ 7.62 (d, J_{HH} = 7.6, 1H), 7.55–7.41 (m, 4H), 7.35-7.38 (m, 1H), 7.26-7.24 (J_{HH} = 8.0, 1H), 6.98 (d, J_{HH} = 8.0, 1H), 6.73 (s, 1H), 6.67–6.63 (m, 1H), 5.46 (s, 1H), 2.03 (s, 3H). ^{13}C NMR (101 MHz, $(CD_3)CO$) 165.3, 158.7, 147.2, 141.4, 139.1, 137.3, 133.3, 130.5, 130.4, 129.5, 129.3, 121.5, 120.5, 102.6, 92.1, 85.9, 77.3, 62.1, 19.1. IR (solid-state ATR, cm^{-1}) 3073, 2952, 2080, 1965, 1702, 1632, 1551, 1432, 1015, 785, 842, 874, 783, 630, 544. IR (CH_2Cl_2, cm^{-1}) 3056 (m), 2958 (w), 1991 (s), 1905 (s), 1726 (m), 1456 (w), 1087 (m). MS; HRMS (ESI$^+$) m/z: $[MH]^+$ calcd for $C_{22}H_{15}MnNO_5$ 428.0325; Found 428.0339.

1.8. H-transfer reactions of manganacycle 2g in neat phenylacetylene (3)

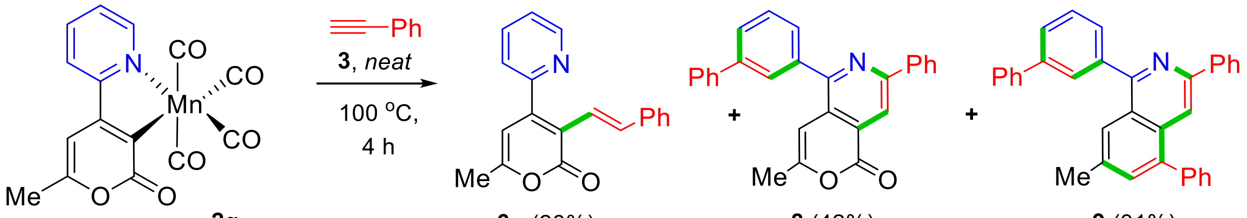

2g **6g** (28%) **8** (42%) **9** (31%)

To a flame-dried Schlenk tube, equipped with a magnetic stir bar, was added manganacycle **2g** (0.28 mmol, 100 mg, 1 eq.) and phenylacetylene **3** (1.5 mL, excess), under nitrogen. The tube was sealed and placed into a pre-heated oil bath at 110 °C and stirred for 4 h. After completion of the reaction (monitored by TLC analysis), the reaction was allowed to cool to room temperature, then filtered through Celite™ and washed with CH_2Cl_2 (30 mL). The filtrate was pre-absorbed on to silica gel and concentrated *in vacuo* (with care). The crude product residue on silica was purified by silica gel column chromatography using gradient elution – petroleum ether/EtOAc/dichloromethane/MeOH = 90:10:0:0 to 0:0:95:5 *v/v*, from which three fractions were isolated and characterized. The first fraction was a light

orange solid (39 mg, 30.8%), identified as compound **9** (X-ray structure obtained). The second fraction was an off-white solid (47 mg, 41.7%), identified as compound **8** (X-ray structure obtained). The third fraction was isolated as a pale orange viscous liquid (23 mg, 28.3%), identified as compound **6g**. All yields are based on the limiting reagent, **2g**. The mass balance is 101%, showing quantitative conversion of **2g** (*within experimental error*). In repeat reactions we noted that another Diels-Alder product was formed in trace amounts, which could not be isolated and fully characterized – see mass spectrum below showing analysis of a crude reaction mixture. We also observed formation of an $Mn(CO)_3$ adduct of compound **8**, which hints at Mn coordination being necessary for the Diels-Alder cycloaddition to the 2-pyridine group.

Data for 6-methyl-3-[(*E*)-2-phenylethenyl]-4-(pyridin-2-yl)-2*H*-pyran-2-one (6g): ^{1}H NMR (400 MHz, CD_2Cl_2) δ 8.76 (ddd, J_{HH} = 4.8, 1.8, 1.0, 1H), 7.87 (d, J_{HH} = 16.1, 1H), 7.81 (td, J_{HH} = 7.7, 1.8, 1H), 7.49 (dt, J_{HH} = 7.7, 1.0, 1H), 7.38–7.25 (br m, ~6H), 6.95 (d, J_{HH} = 16.1, 1H), 6.31 (s, 1H), 2.32 (s, 3H). ^{13}C NMR (101 MHz, CD_2Cl_2) δ 162.4, 159.9, 155.9, 151.0, 150.7, 138.4, 136.9, 134.8, 129.1, 128.4, 127.2, 125.5, 124.2, 121.6, 118.2, 107.0, 20.3. IR (solid-state ATR, cm^{-1}) 340, 288, 269, 2970, 3015, 1739, 1367, 1263, 1216, 836, 795, 687. MS; HRMS (ESI$^+$) m/z: [MH]$^+$ calcd for $C_{19}H_{16}NO_2$ 290.1176; Found 290.1177.

Data for 5-([1,1'-biphenyl]-3-yl)-3-methyl-7-phenyl-1H-pyrano[4,3-c]pyridin-1-one (8): MP 115-116 °C. ^{1}H NMR (400 MHz, CD_2Cl_2) δ 8.50 (d, J_{HH} = 1.0, 1H), 8.22–8.18 (m, 2H), 7.94 (td, J_{HH} = 1.8, 0.6, 1H), 7.78 (dt, J_{HH} = 7.1, 1.8, 1H), 7.71–7.66 (m, 3H), 7.67–7.62 (m, 2H), 7.53–7.37 (m, 5H), 6.53-6.54 (m, 1H), 2.28 (d, J = 1.0, 3H). ^{13}C NMR (176 MHz, CD_2Cl_2) δ 162.4, 157.1, 156.2, 155.2, 142.1, 141.2, 139.5, 138.6, 130.0, 129.5, 129.5, 129.4, 129.1, 128.8, 128.5, 128.2, 127.8, 127.4, 116.7, 101.0, 20.5. IR (solid-state ATR, cm^{-1}) 2081, 1966, 1927, 1681, 1633, 1596, 1259, 1015. MS; HRMS (ESI$^+$) m/z: [MH]$^+$ calcd for $C_{27}H_{20}NO_2$ 390.1494; Found 390.1461.

Data for 1-([1,1'-biphenyl]-3-yl)-3-methyl-7-phenyl-6-phenylisoquinoline (9): MP 150.5–152.0 °C. ^{1}H NMR (700 MHz, CD_2Cl_2) δ 8.23 (d, J_{HH} = 7.7, 2H), 8.12 (s, 1H), 8.06 (s, 1H), 8.04 (s, 1H), 7.83 (s, 1H), 7.81–7.79 (m, 2H), 7.73 (d, J_{HH} = 7.7, 2H), 7.68 (ddd, J_{HH} = 8.0, 7.4, 0.5, 1H), 7.51–7.38 (br m, ~12H), 2.37 (d, J_{HH} = 0.7, 3H). ^{13}C NMR (176 MHz, CD_2Cl_2) δ 159.6, 150.0, 145.4, 141.5, 141.2, 141.1, 140.7, 139.8, 136.6, 135.8, 129.3, 129.2, 129.1, 129.0, 128.8, 128.5, 128.45, 128.0, 127.8, 127.7, 127.6, 127.5, 127.1, 125.4, 123.7, 115.7, 21.7. IR (solid-state ATR, cm^{-1}) 3054, 3029, 2958, 2921, 2853, 2008, 1931, 1553, 1477, 1446, 1378, 1313, 1259, 1073, 1024, 898, 803. MS; HRMS (ESI$^+$) m/z: [M + H]$^+$ calcd for $C_{34}H_{26}N$ 448.2065; Found 448.2035.

Mass spectrum of crude reaction mixture:

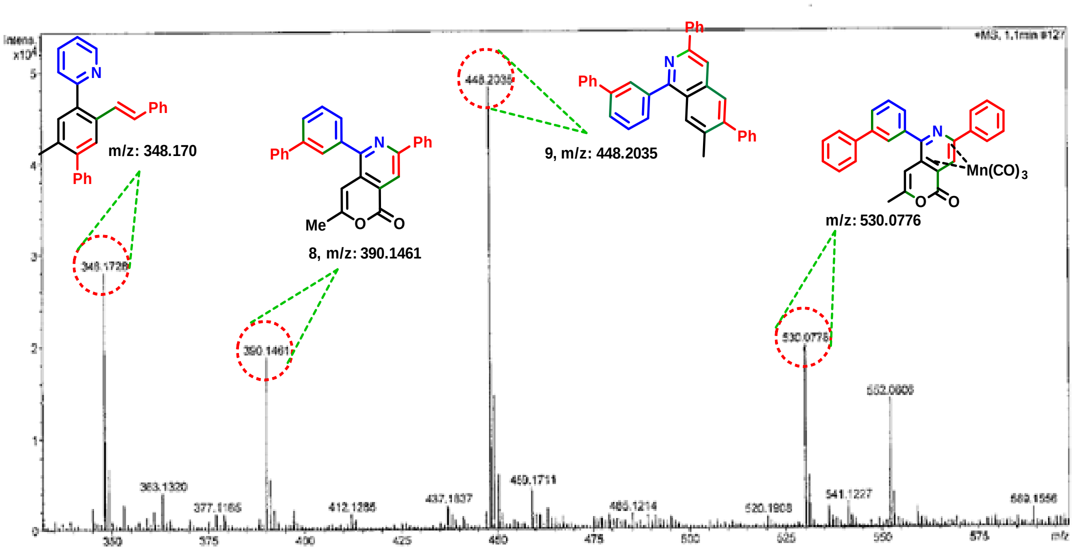

1.9. Synthesis of η^4-tricarbonylmanganese(I) complex (10)

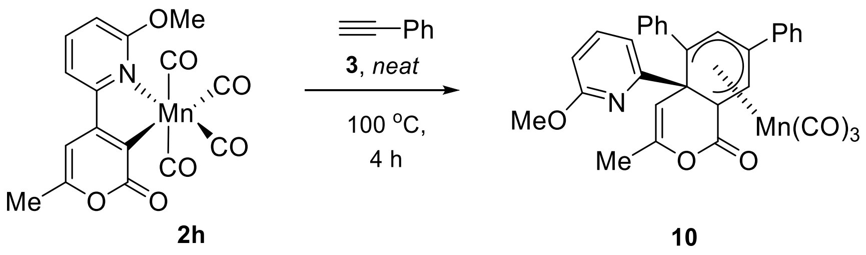

Complex **2h** (100 mg, 0.26 mmol) was reacted with neat phenylacetylene **3** (1.5 mL, in excess) for 4 h. The crude product was purified on silica gel by column chromatography using petroleum ether/EtOAc (90:10, *v*/*v*) as the eluent. Complex **10** was isolated as a pale orange solid (107 mg, 73 %). MP 110.5-111.0 °C. ^{1}H NMR (400 MHz, CD_2Cl_2) δ 7.71 (dd, J_{HH} = 7.9, 1.7, 2H), 7.46 (s, 1H), 7.46–7.29 (m, 9H), 6.61 (d, J_{HH} = 7.5, 1H), 6.51 (d, J_{HH} = 7.5, 1H), 6.49 (s, 1H), 5.93 (d, J_{HH} = 1.1, 1H), 5.47 (s, 1H), 3.71 (s, 3H), 2.03 (s, 3H). ^{13}C NMR (176 MHz, CD_2Cl_2) δ 168.8, 164.0, 162.8, 149.7, 139.7, 137.5, 136.2, 129.9, 129.6, 129.5, 128.9, 128.8, 127.0, 112.0, 109.8, 104.2, 100.3, 95.1, 86.9, 48.7, 44.3, 19.7. IR (solid-state ATR, cm^{-1}) 3087, 3027, 2923, 2853, 2073, 1993, 1946, 1634, 1599, 1261, 1260, 1093, 1049, 782. MS; HRMS (ESI$^+$) m/z: $[M + H]^+$ calcd for $C_{31}H_{24}MnNO_6$ 560.0906; Found 560.0859.

2. Representative NMR spectra of compounds

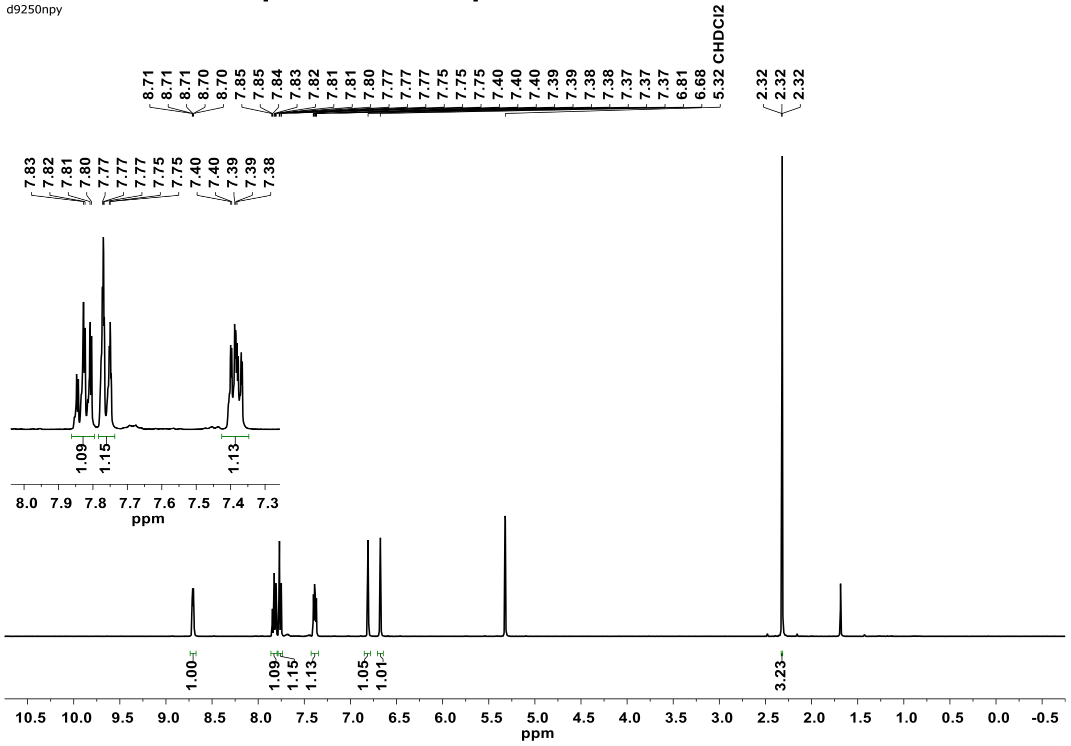

Figure 6 ^{1}H NMR spectrum for compound 1g.

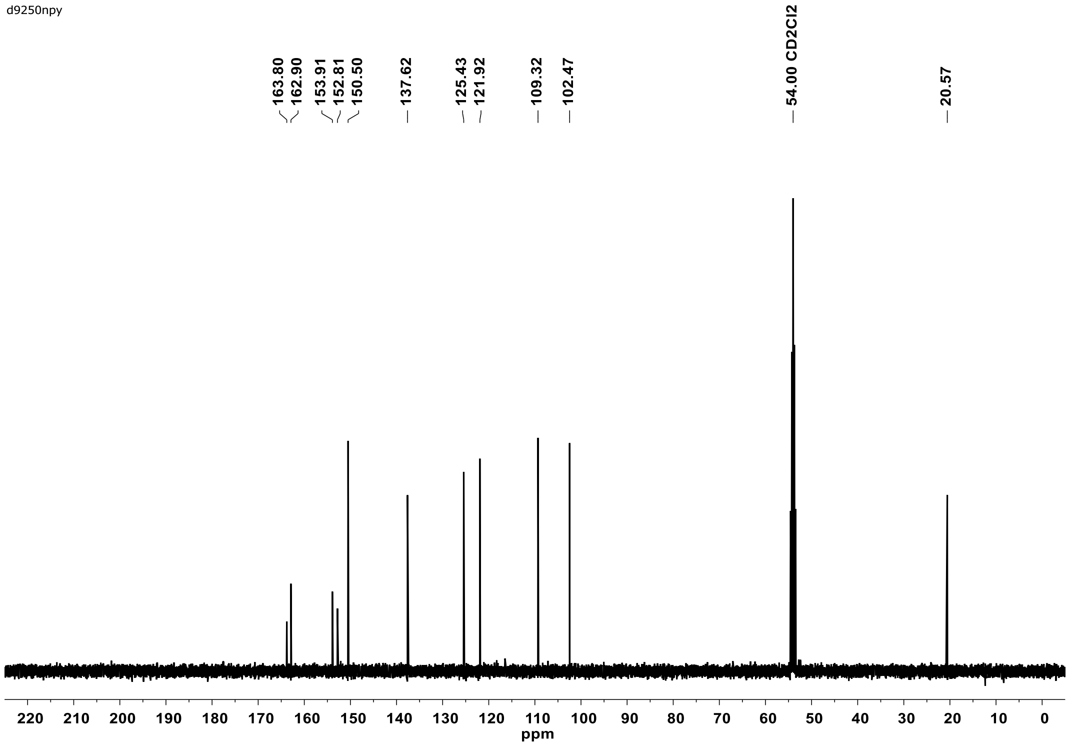

Figure 7 ^{13}C NMR spectrum for compound 1g.

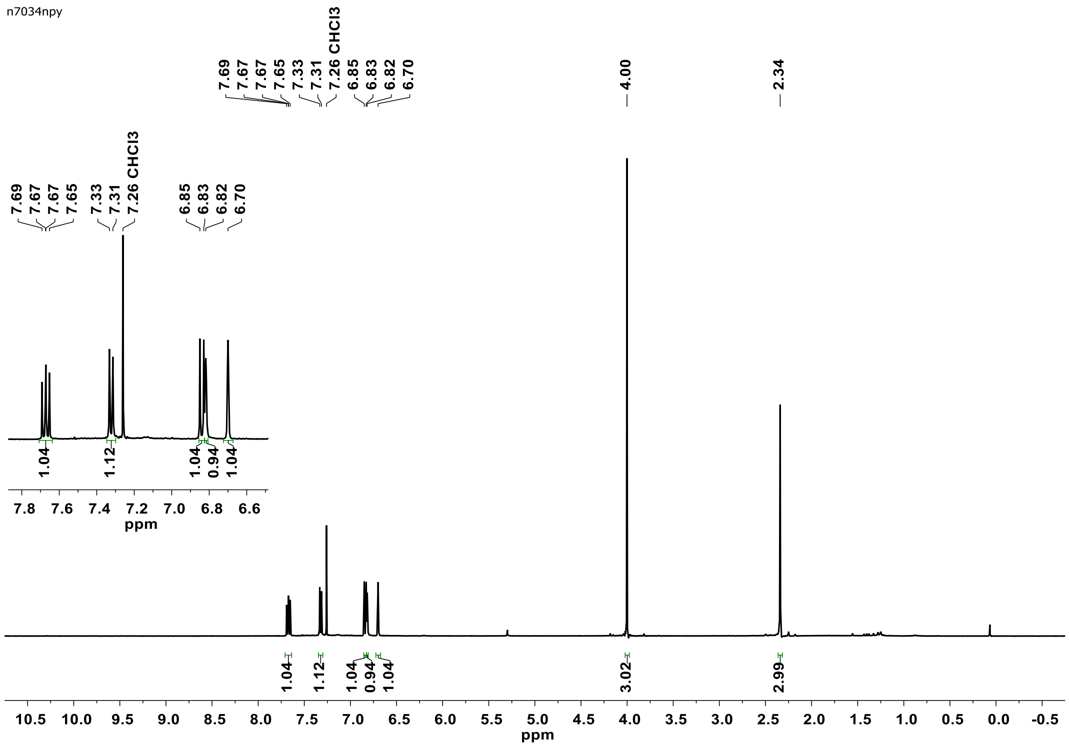

Figure 8 ^{1}H NMR spectrum for compound 1h.

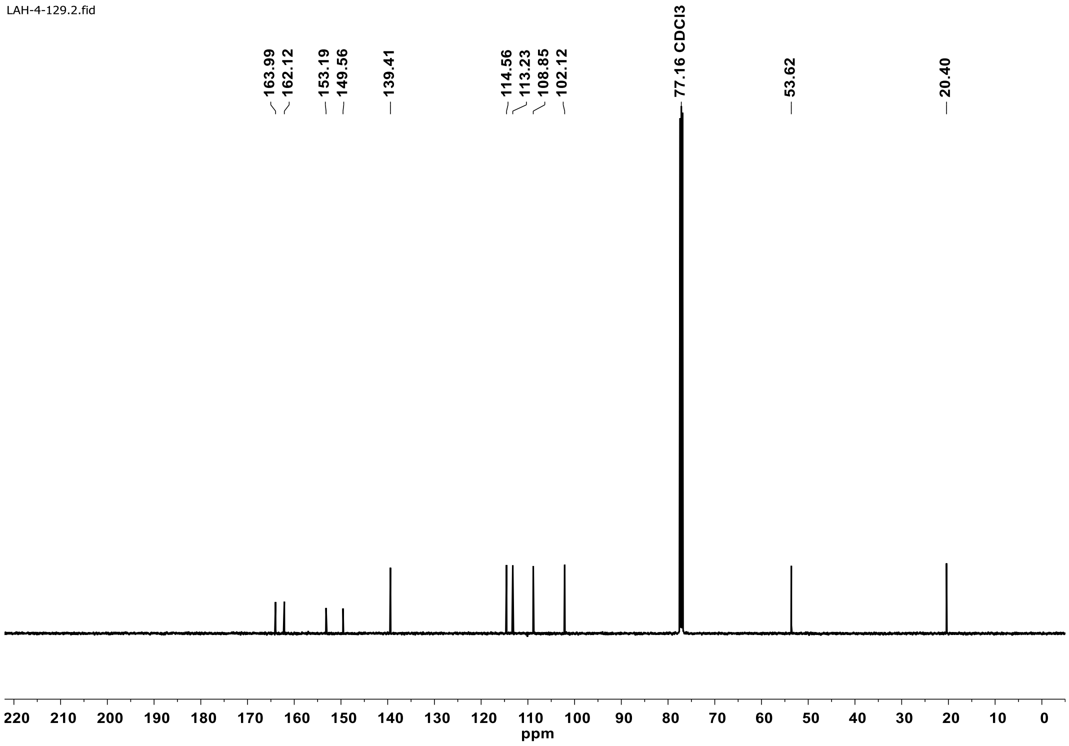

Figure 9 ^{13}C NMR spectrum for compound 1h.

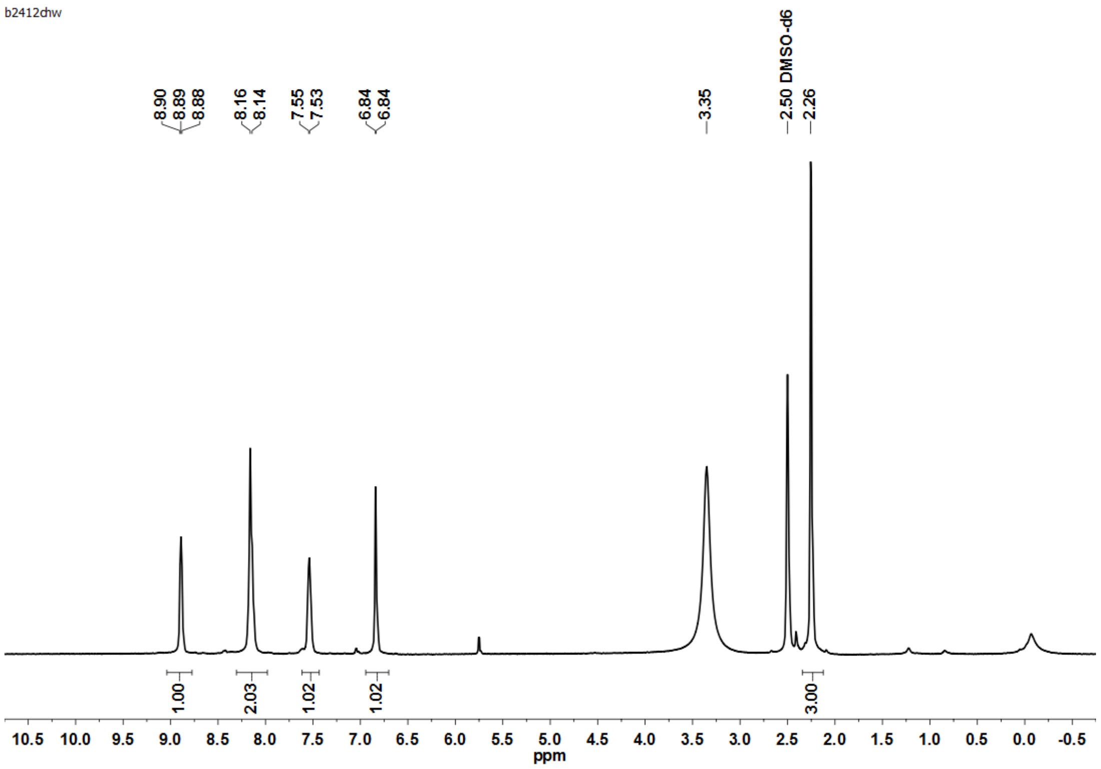

Figure 10 ^{1}H NMR spectrum for compound 2g.

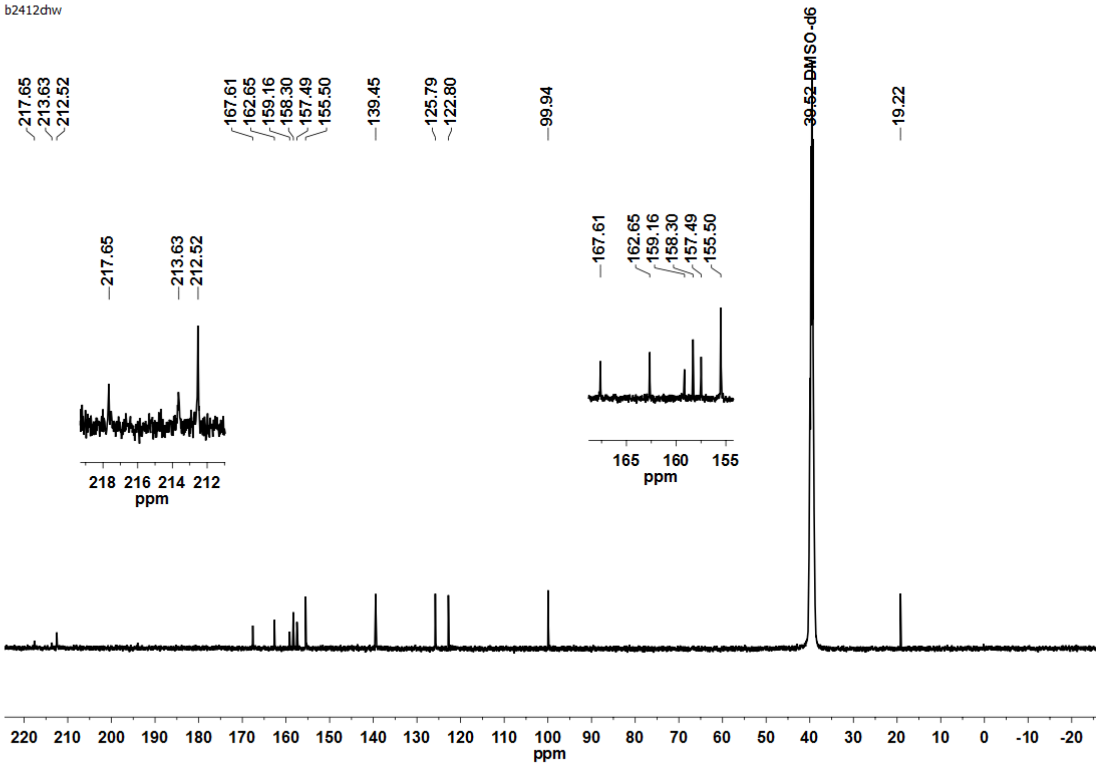

Figure 11 ^{13}C NMR spectrum for compound 2g.

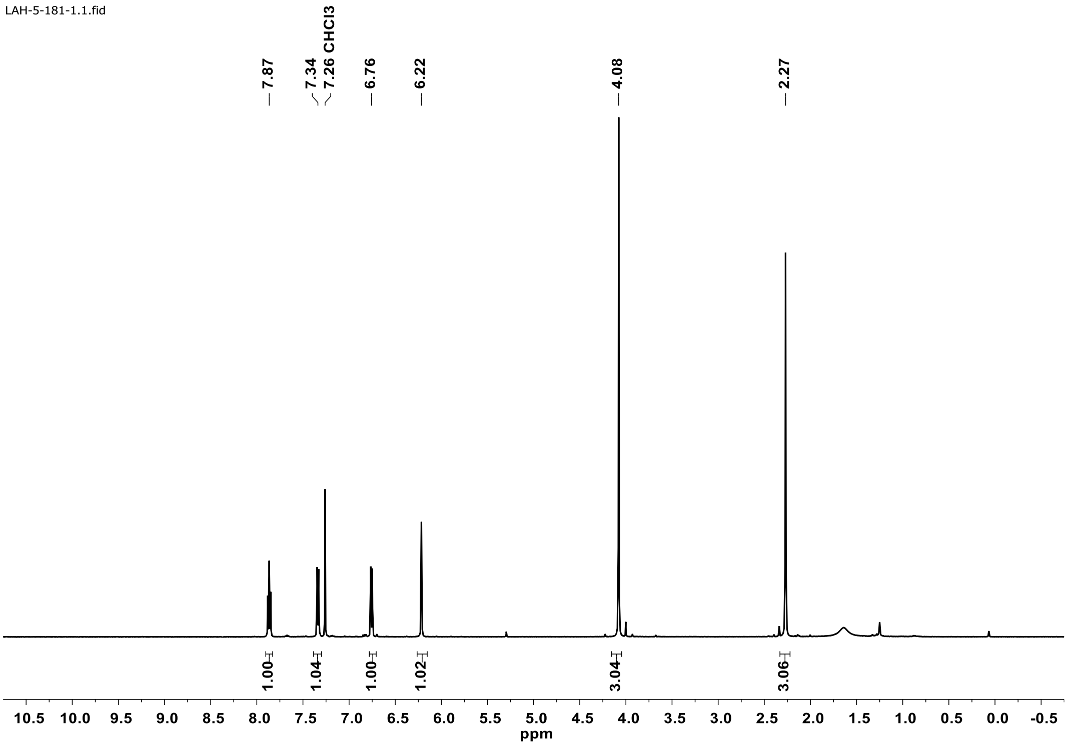

Figure 12 ^{1}H NMR spectrum for compound 2h.

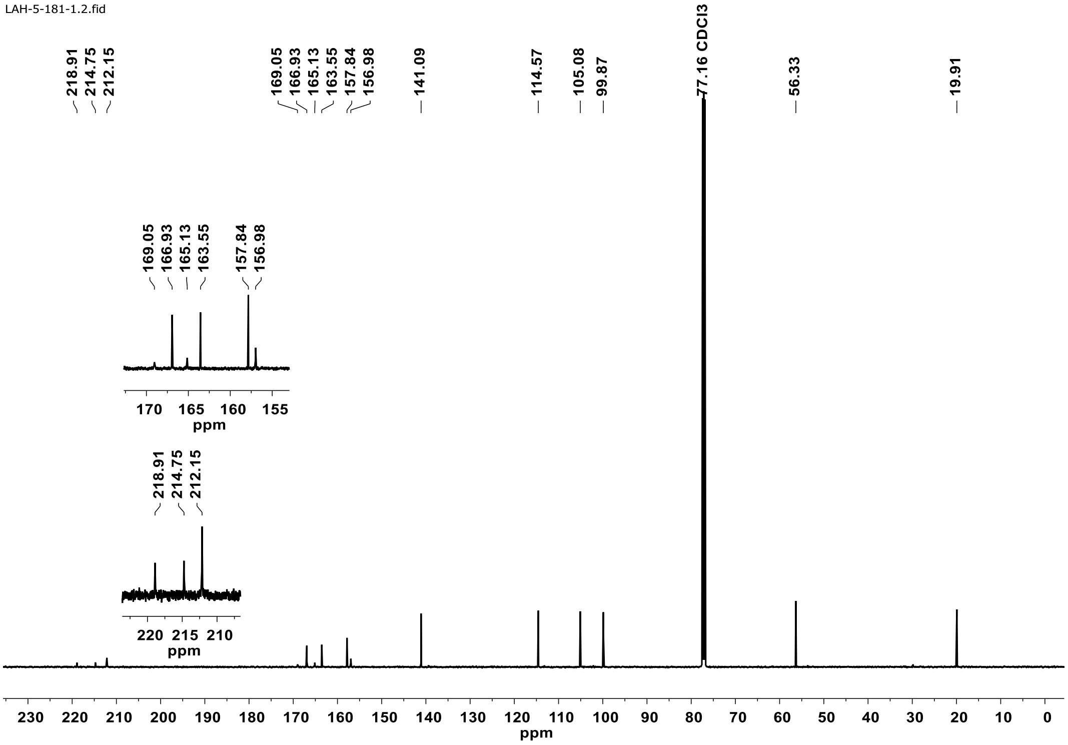

Figure 13 ^{13}C NMR spectrum for compound 2h.

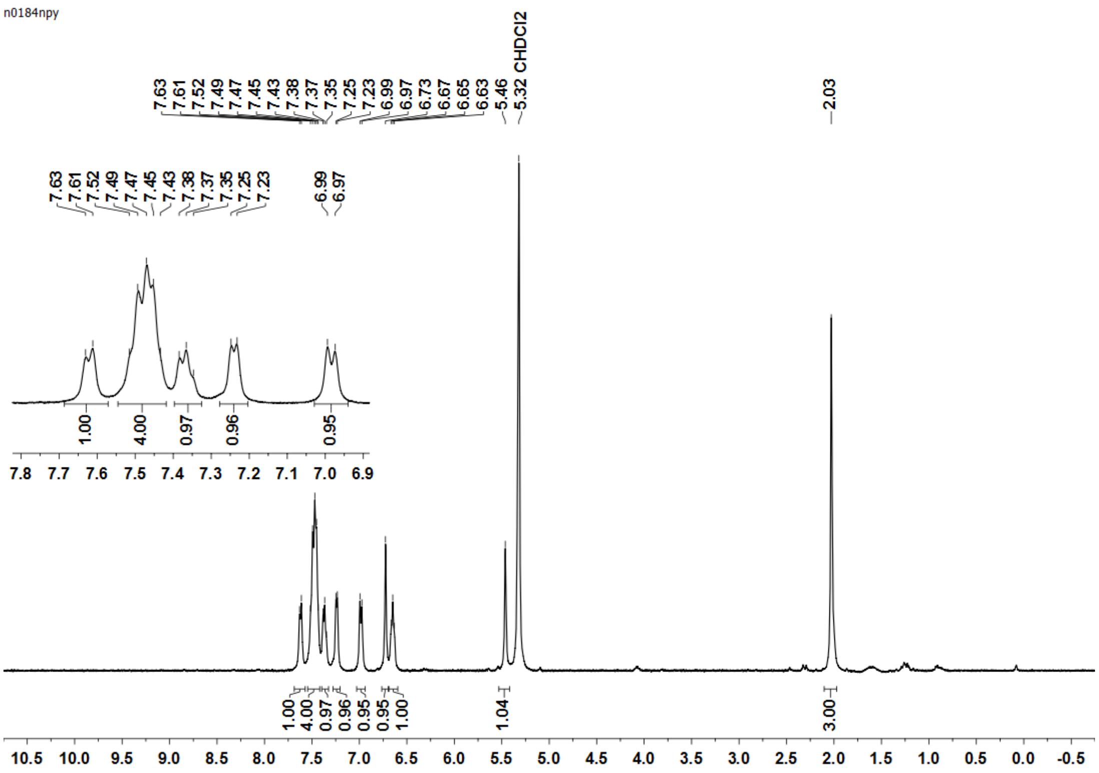

Figure 14 ^{1}H NMR spectrum for compound 5g.

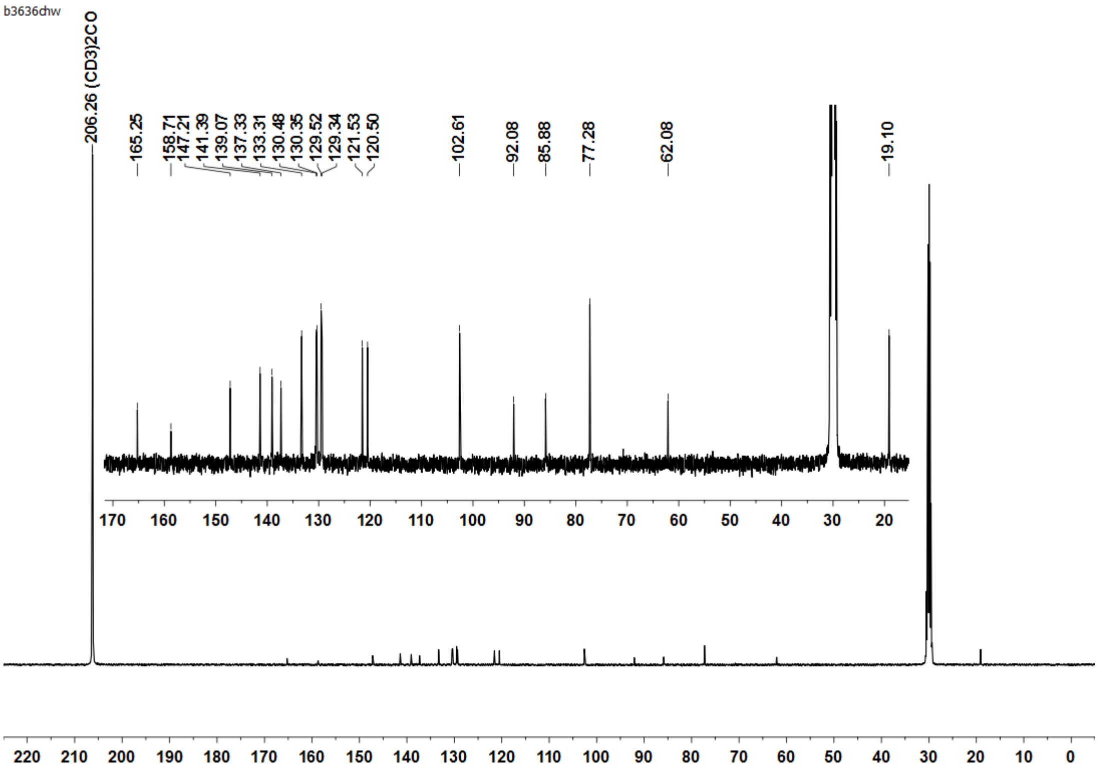

Figure 15 ^{13}C NMR spectrum for compound 5g.

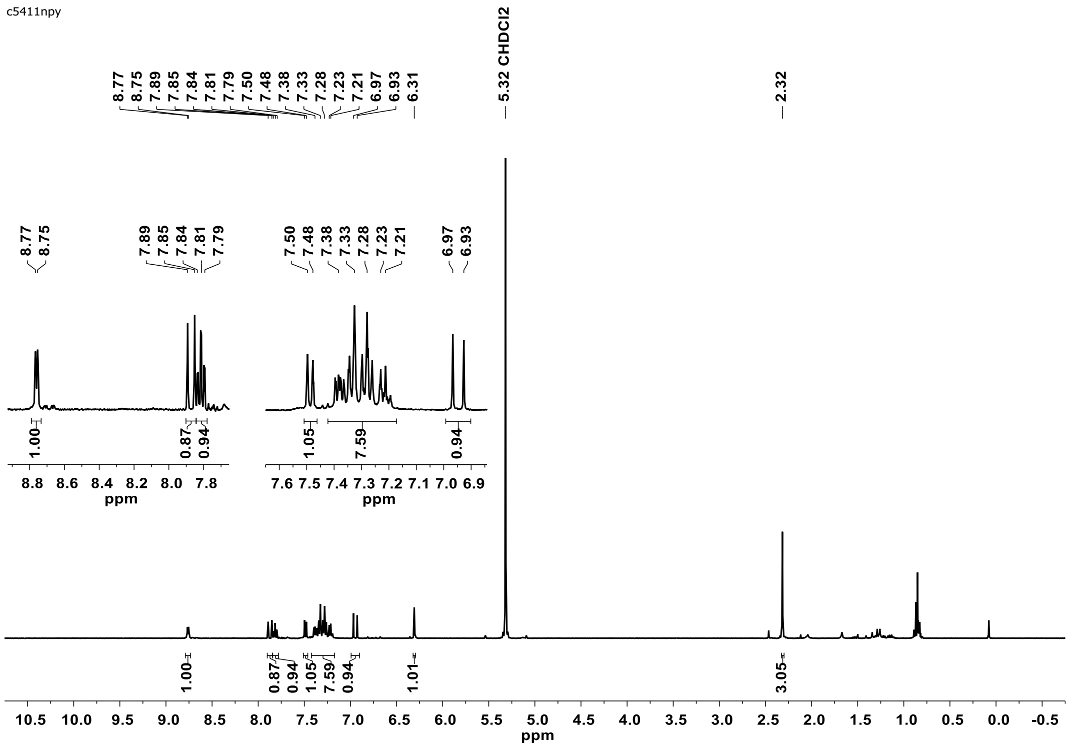

Figure 16 ^{1}H NMR spectrum for compound 6g.

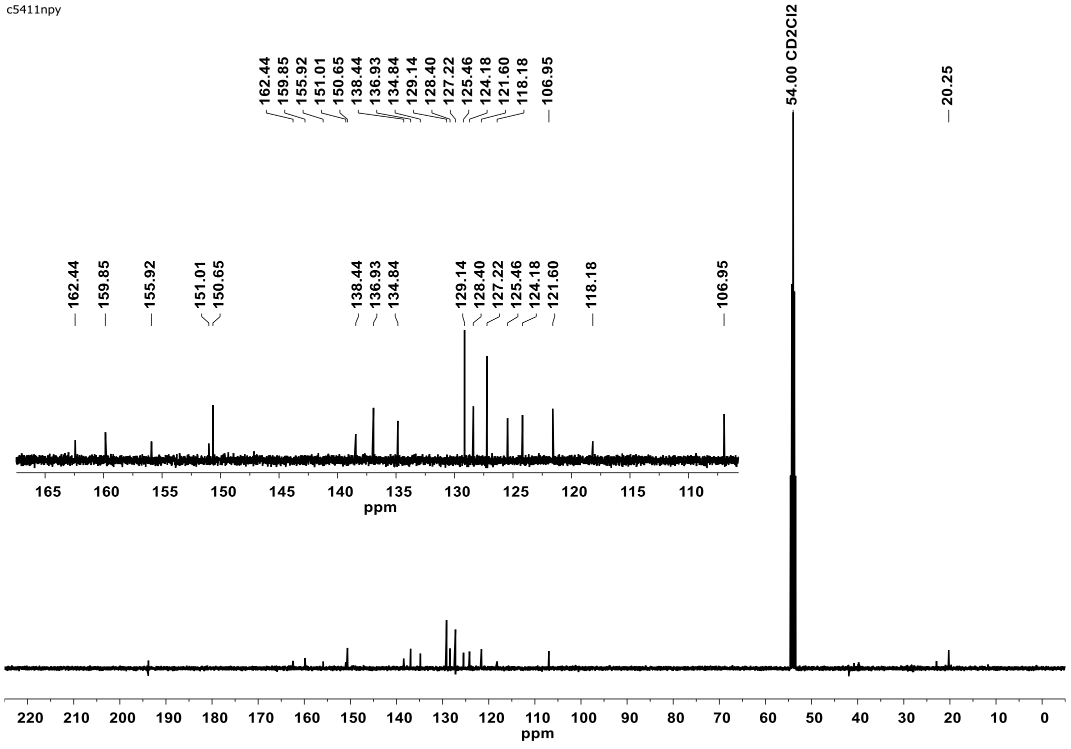

Figure 17 ^{13}C NMR spectrum for compound 6g.

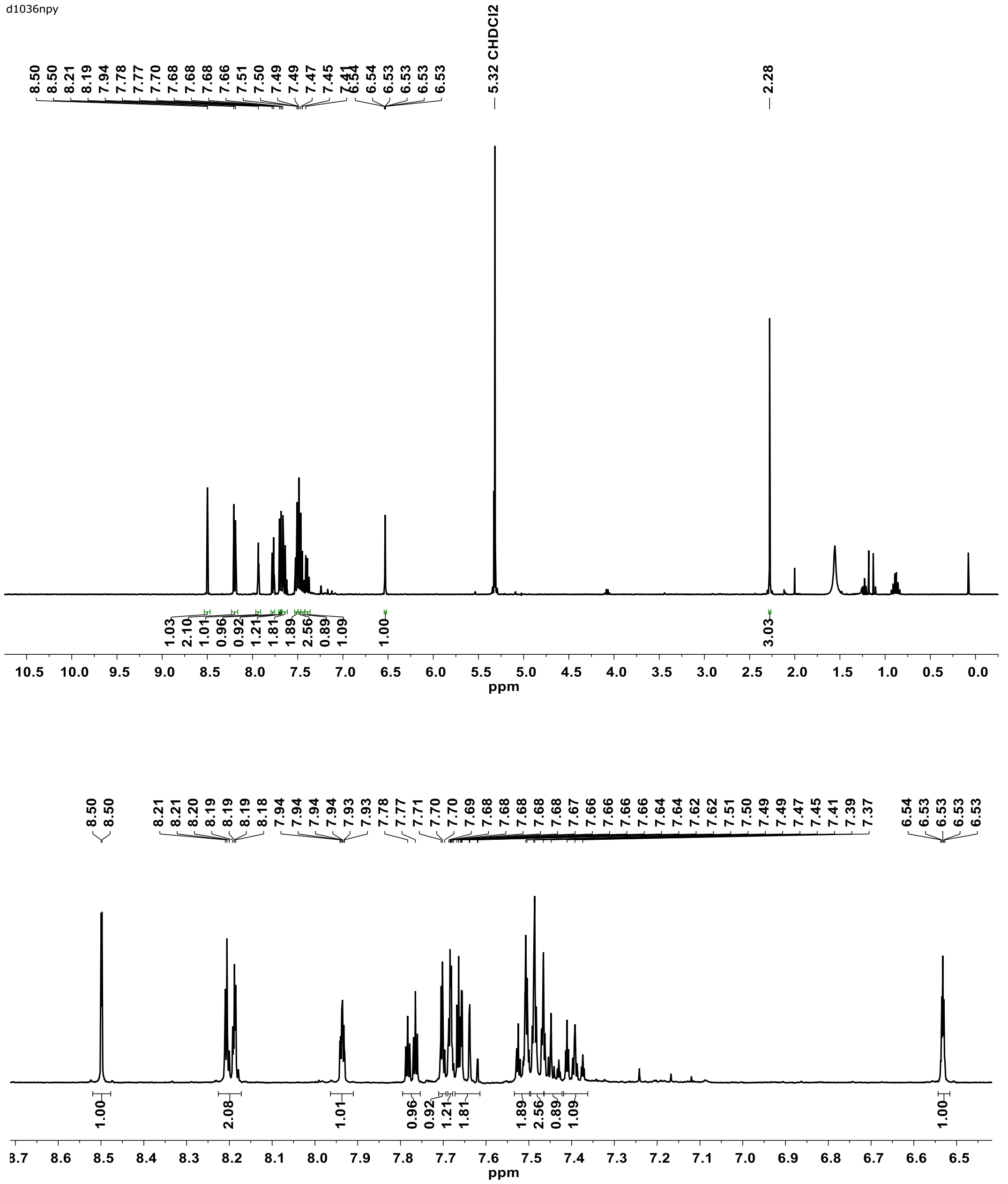

Figure 18 ^{1}H NMR spectrum for compound 8.

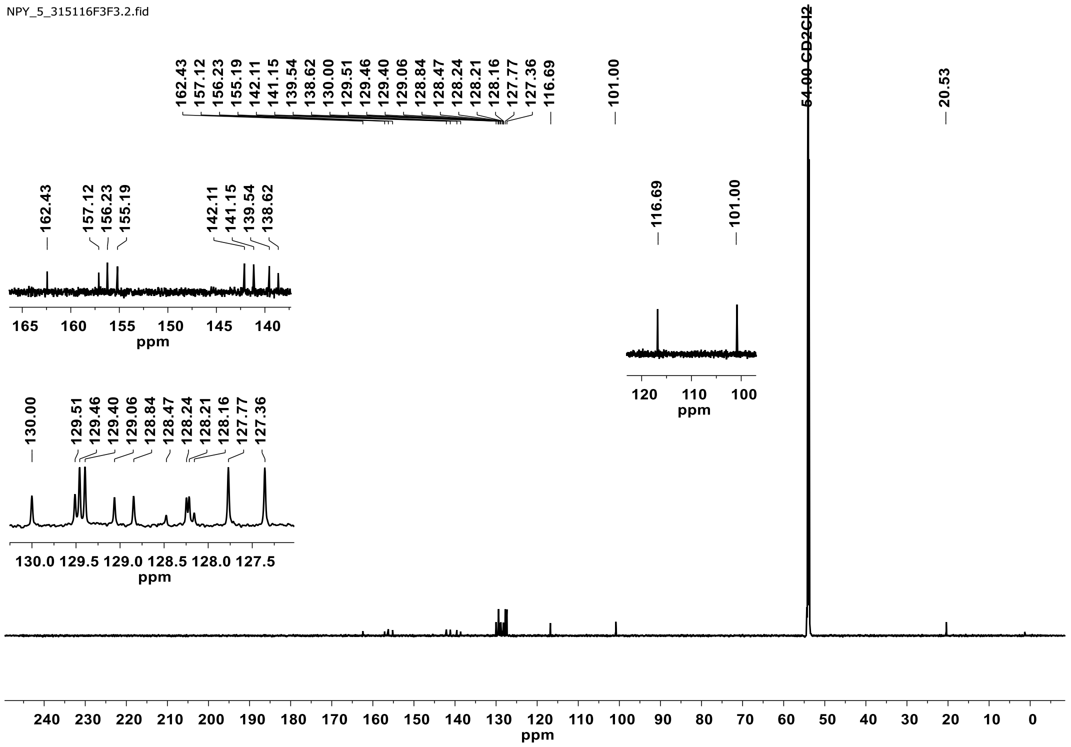

Figure 19 ^{13}C NMR spectrum for compound 8.

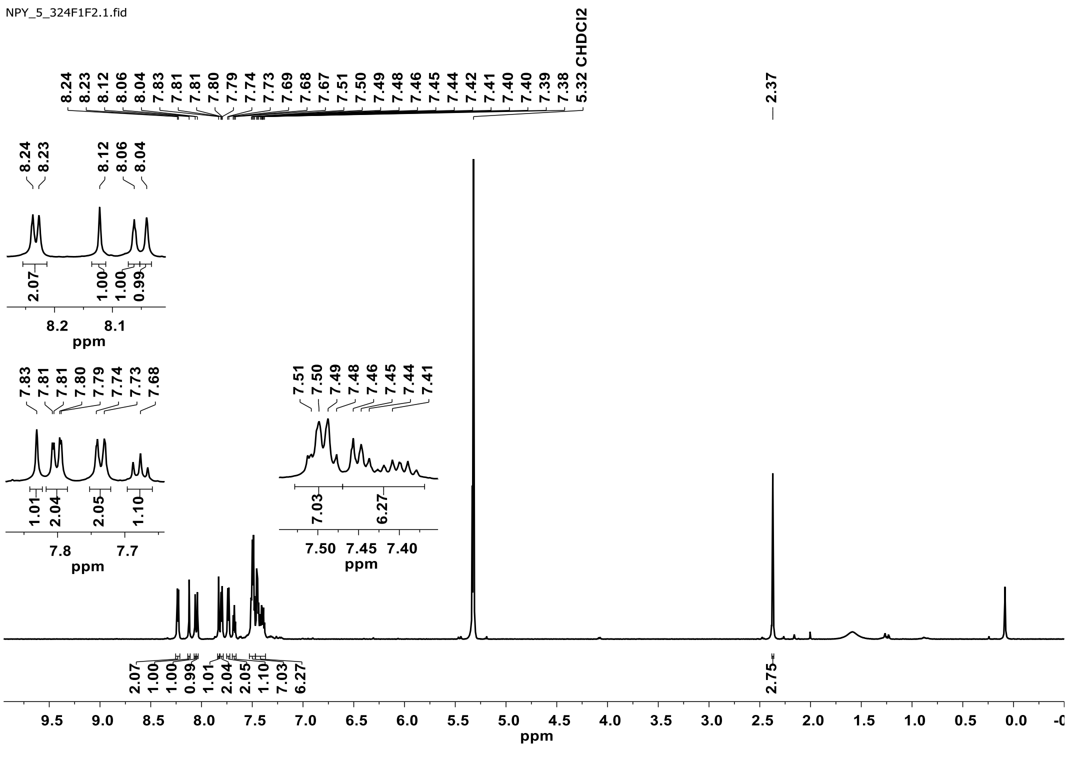

Figure 20 ^{1}H NMR spectrum for compound 9.

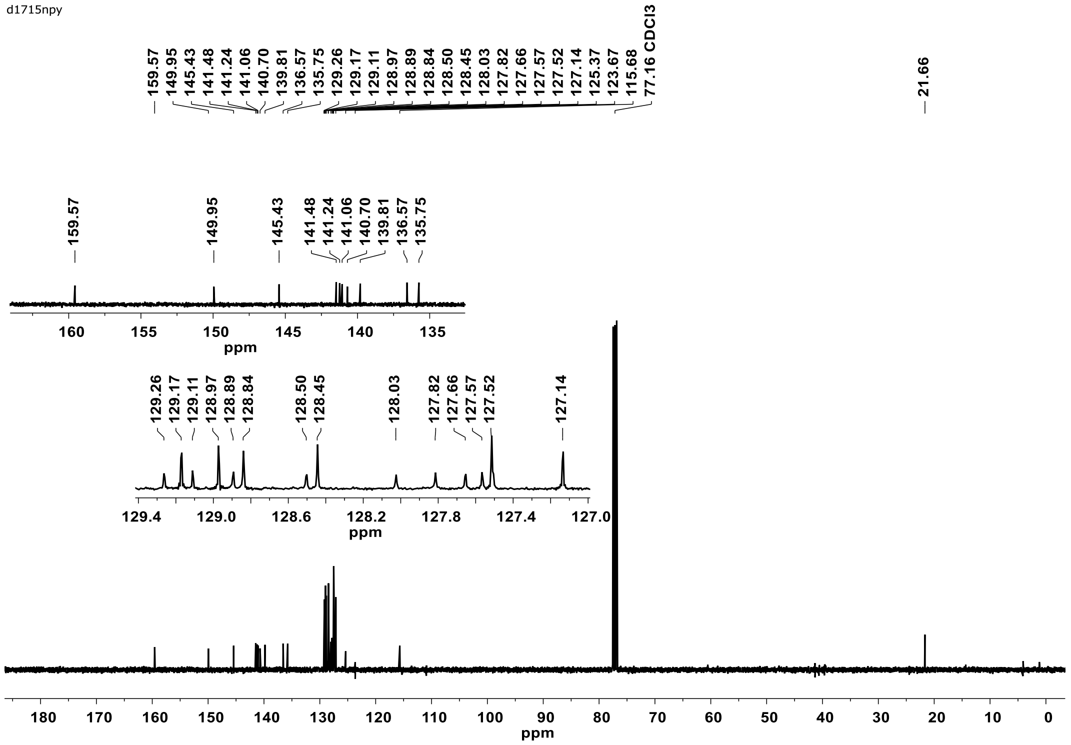

Figure 21 ^{13}C NMR spectrum for compound 9.

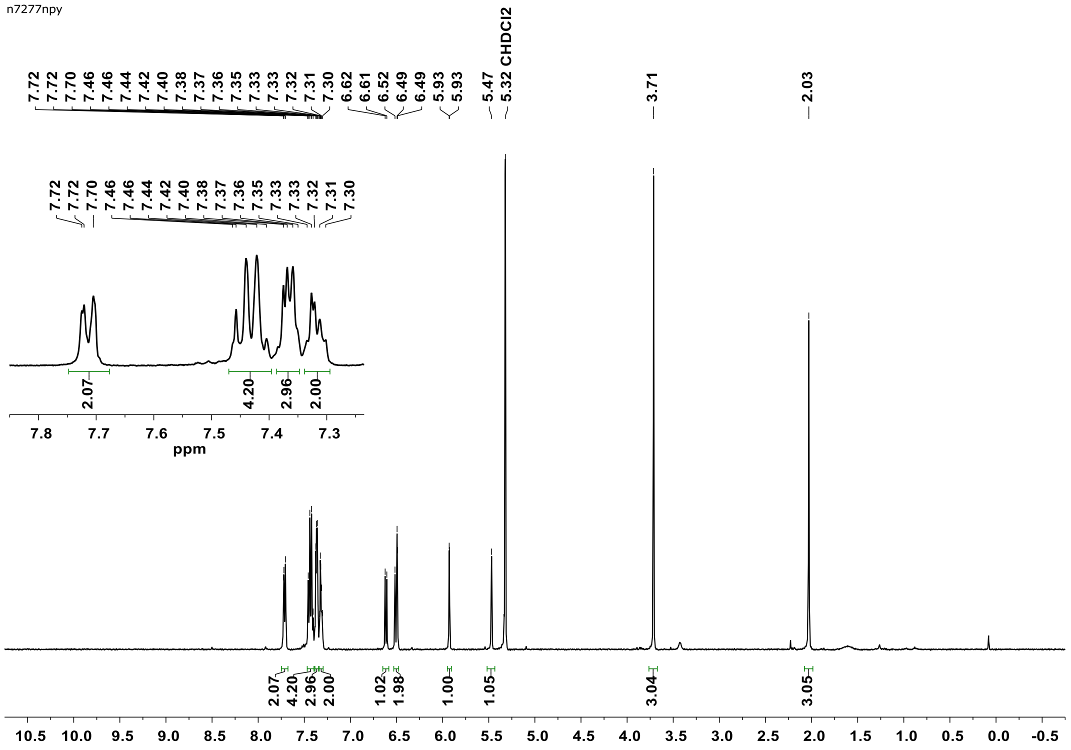

Figure 22 ^{1}H NMR spectrum for compound 10.

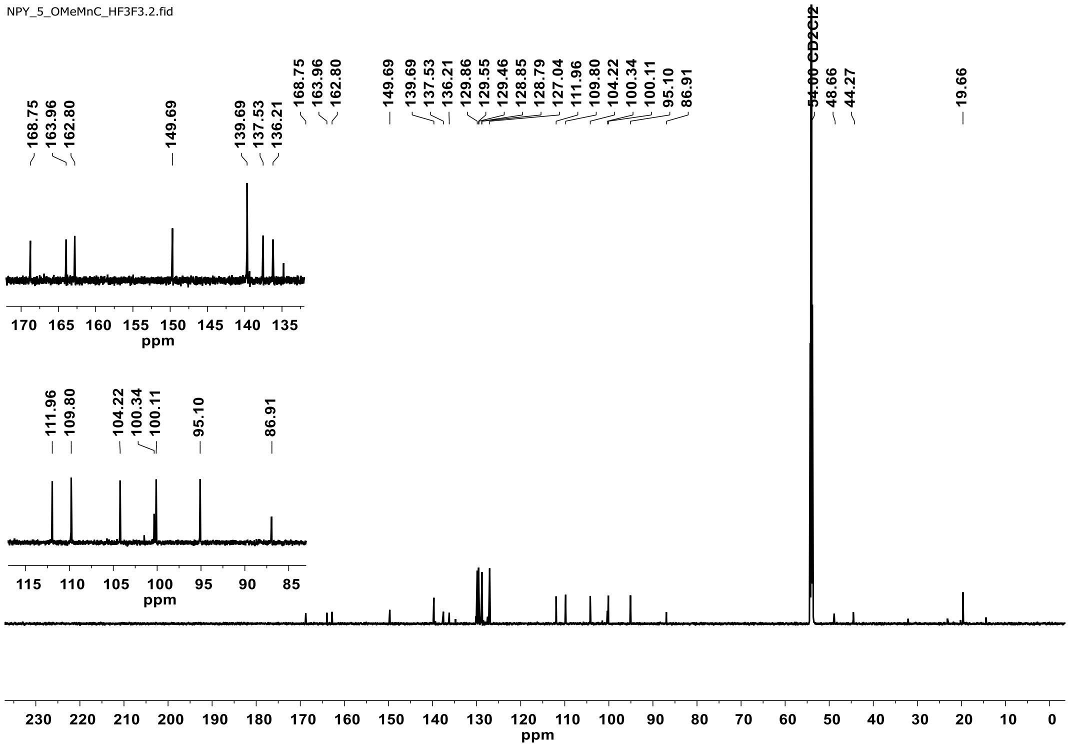

Figure 23 ^{13}C NMR spectrum for compound 10.

3. X-ray structure details

3.1. X-ray structure for complex 2g

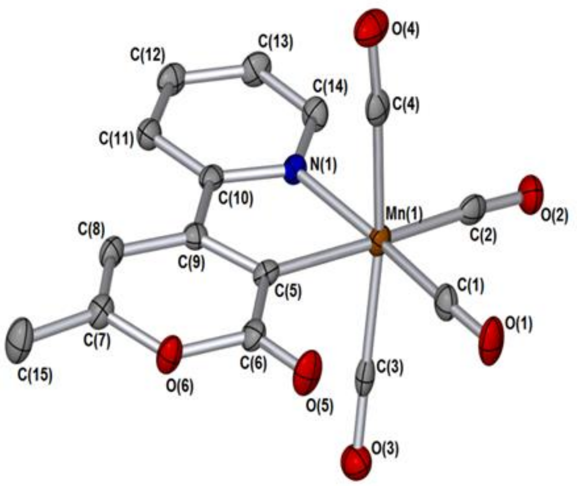

checkCIF/PLATON report

No syntax errors found. CIF dictionary Interpreting this report

Datablock: ijsf1305

Bond precision: C-C = 0.0037 A Wavelength=0.71070

Cell:	a=16.2458(5)	b=13.2007(5)	c=13.3097(7)
	alpha=90	beta=104.685(4)	gamma=90
Temperature:	110 K		

	Calculated	Reported
Volume	2761.1(2)	2761.11(19)
Space group	C 2/c	C 1 2/c 1
Hall group	-C 2yc	-C 2yc
Moiety formula	C15 H8 Mn N O6	C15 H8 Mn N O6
Sum formula	C15 H8 Mn N O6	C15 H8 Mn N O6
Mr	353.16	353.16
Dx,g cm-3	1.699	1.699
Z	8	8
Mu (mm-1)	0.989	0.989
F000	1424.0	1424.0
F000'	1427.43	
h,k,lmax	21,17,17	20,16,17
Nref	3329	2814
Tmin,Tmax	0.930,0.976	0.852,0.943
Tmin'	0.913	

Correction method= ANALYTICAL

Data completeness= 0.845 Theta(max)= 27.990

R(reflections)= 0.0402(2261) wR2(reflections)= 0.0887(2814)

S = 1.064 Npar= 209

Selected bond lengths (Å) for **2g**: C1-Mn1 = 1.817(3), C2-Mn1 = 1.827(3), C3-Mn = 11.842(3), C9-C10 = 1.467(3), C4-Mn1 = 1.866(3), C5-Mn1 = 2.042(2), Mn1-N1 = 2.0651(19). H-atoms omitted for clarity. Thermal ellipsoids set to 50%. Arbitrary numbering system used.

3.2. X-ray structure for complex 2h

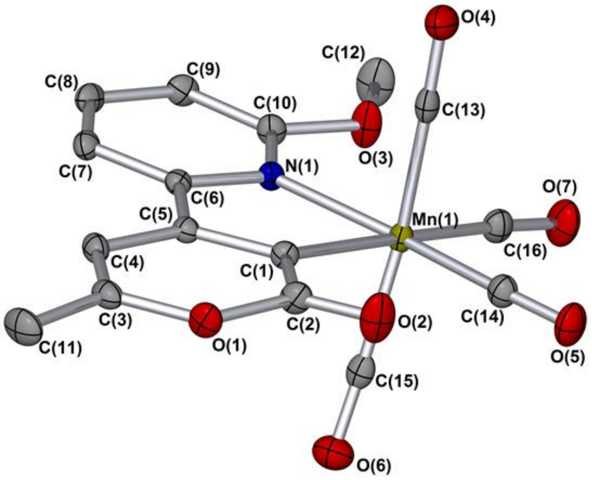

checkCIF/PLATON report

No syntax errors found. CIF dictionary Interpreting this report

Datablock: ijsf1510

Bond precision: C-C = 0.0021 A Wavelength=0.71070

Cell:	a=6.9469(5)	b=10.1635(6)	c=12.3849(9)
	alpha=67.194(6)	beta=83.812(6)	gamma=71.460(7)
Temperature:	110 K		

	Calculated	Reported
Volume	764.12(10)	764.12(10)
Space group	P -1	P -1
Hall group	-P 1	-P 1
Moiety formula	C16 H10 Mn N O7	C16 H10 Mn N O7
Sum formula	C16 H10 Mn N O7	C16 H10 Mn N O7
Mr	383.19	383.19
Dx,g cm-3	1.666	1.665
Z	2	2
Mu (mm-1)	0.905	0.905
F000	388.0	388.0
F000'	388.88	
h,k,lmax	10,15,18	10,15,17
Nref	5351	4850
Tmin,Tmax	0.917,0.946	0.829,0.956
Tmin'	0.743	

Correction method= # Reported T Limits: Tmin=0.829 Tmax=0.956
AbsCorr = ANALYTICAL

Data completeness= 0.906 Theta(max)= 32.075

R(reflections)= 0.0349(4144) wR2(reflections)= 0.0779(4850)

S = 1.056 Npar= 228

Selected bond lengths (Å) for **2h**: C1 -Mn1 = 2.0357(15), C13-Mn1 = 1.8487(17), C14-Mn1 = 1.8097(16), C15-Mn1 = 1.8617(17), C16-Mn1 = 1.8396(17), Mn1-N1 = 2.1032(13). H-atoms omitted for clarity. Thermal ellipsoids set to 50%. Arbitrary numbering system used.

3.3. X-ray structure for complex 5g

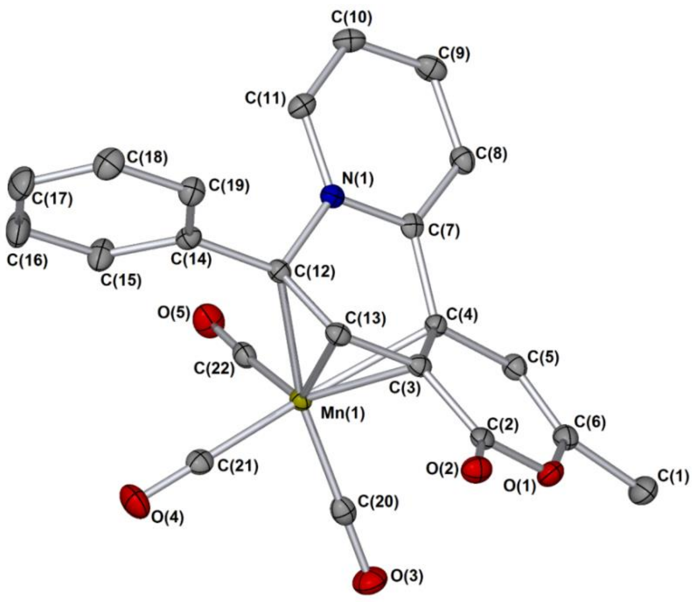

checkCIF/PLATON report

No syntax errors found. CIF dictionary Interpreting this report

Datablock: ijsf1416

Bond precision:	C-C = 0.0028 A		Wavelength=0.71073
Cell:	a=7.4809(3)	b=19.0446(15)	c=12.8808(4)
	alpha=90	beta=93.949(3)	gamma=90
Temperature:	110 K		

	Calculated	Reported
Volume	1830.78(17)	1830.78(17)
Space group	P 21/c	P 1 21/c 1
Hall group	-P 2ybc	-P 2ybc
Moiety formula	C22 H14 Mn N O5	C22 H14 Mn N O5
Sum formula	C22 H14 Mn N O5	C22 H14 Mn N O5
Mr	427.28	427.28
Dx,g cm-3	1.550	1.550
Z	4	4
Mu (mm-1)	0.757	0.757
F000	872.0	872.0
F000'	873.74	
h,k,lmax	10,27,18	10,26,18
Nref	5513	4803
Tmin,Tmax	0.951,0.984	0.924,0.984
Tmin'	0.857	

Correction method= GAUSSIAN

Data completeness= 0.871 Theta(max)= 30.344

R(reflections)= 0.0374(3963) wR2(reflections)= 0.0876(4803)

S = 1.048 Npar= Npar = 263

Selected bond lengths (Å) for **5g**: C3-Mn1 = 2.0769(17), C4-Mn1 = 2.1843(17), C12-Mn1 = 2.1060(18), C13-Mn1 = 2.0908(18), C20-Mn1 = 1.816(2), C21-Mn1 = 1.8034(19), C22-Mn1 = 1.797(2); bond angles (°) C3-C4-C7 = 116.31(15); C13-C12-N1 = 114.38(14); Selected torsion angle (°) C4-C3-C13-C12 = 4.2(2). H-atoms omitted for clarity. Thermal ellipsoids set to 50%. Arbitrary numbering system used. Arbitrary numbering system used.

3.4. X-ray structure for compound 8

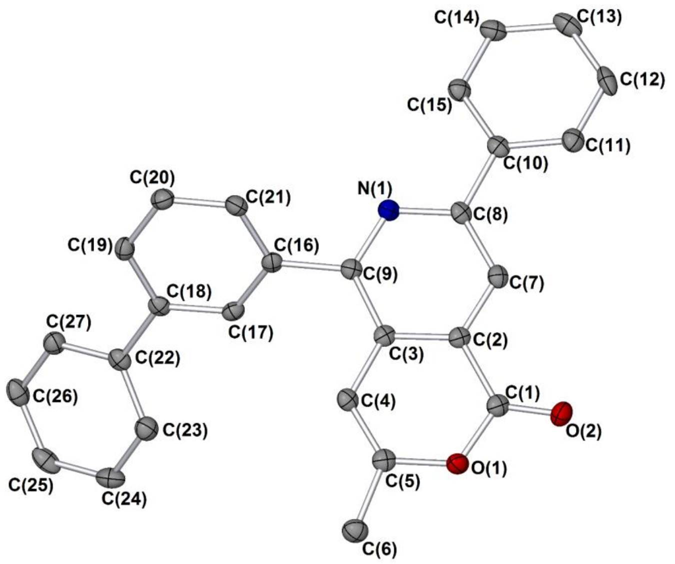

checkCIF/PLATON report

No syntax errors found. CIF dictionary Interpreting this report

Datablock: ijsf1489

No errors found in this datablock

Bond precision: C-C = 0.0020 A Wavelength=1.54184

Cell: a=11.4500(3) b=13.3633(3) c=12.4539(3)
alpha=90 beta=93.005(2) gamma=90

Temperature: 110 K

	Calculated	Reported
Volume	1902.95(8)	1902.96(8)
Space group	P 21/c	P 1 21/c 1
Hall group	-P 2ybc	-P 2ybc
Moiety formula	C27 H19 N O2	C27 H19 N O2
Sum formula	C27 H19 N O2	C27 H19 N O2
Mr	389.43	389.43
Dx,g cm-3	1.359	1.359
Z	4	4
Mu (mm-1)	0.677	0.677
F000	816.0	816.0
F000'	818.36	
h,k,lmax	13,15,14	13,15,14
Nref	3394	3383
Tmin,Tmax	0.899,0.942	0.974,0.988
Tmin'	0.854	

Correction method= # Reported T Limits: Tmin=0.974 Tmax=0.988
AbsCorr = GAUSSIAN

Data completeness= 0.997 Theta(max)= 67.072

R(reflections)= 0.0393(2995) wR2(reflections)= 0.1024(3383)

S = 1.038 Npar= 272

Selected bond lengths (Å) for **8**: C9-C16 = 1.4895(19); N1-C9 = 1.3336(18); O1-C1 = 1.3690(17); Selected torsion angle (°) N1-C8-C10-C15 = -11.90(19); N1-C9-C16-C21 = -48.34(17); C19-C18-C22-C27 = 24.7(2). H-atoms omitted for clarity. Thermal ellipsoids set to 50%. Arbitrary numbering system used.

3.5. X-ray structure for compound 9

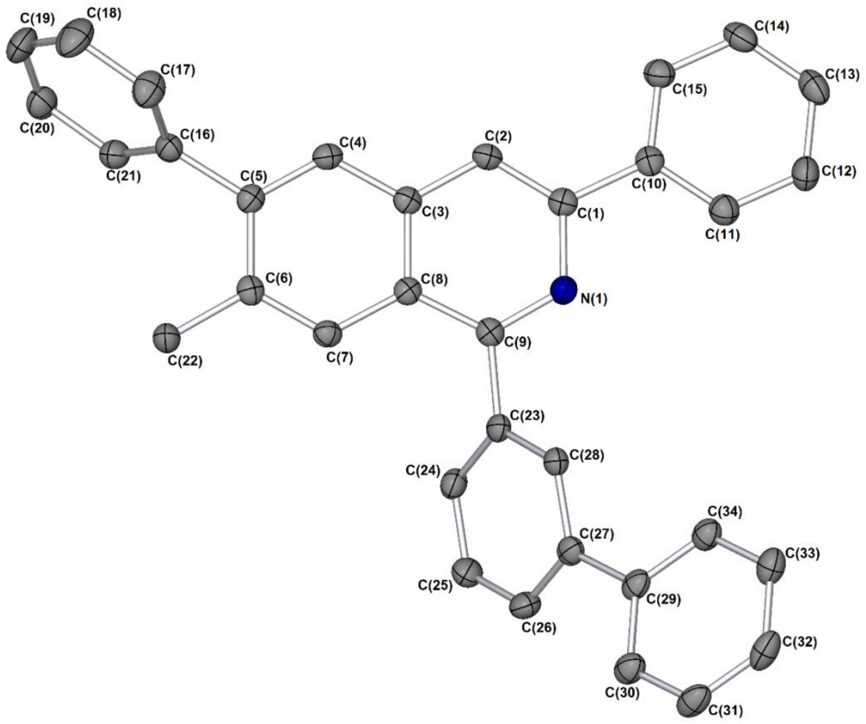

Datablock: ijsf1516a

```
Bond precision:  C-C = 0.0023 A          Wavelength=1.54184

Cell:            a=13.86719(18)     b=13.86719(18)     c=12.4237(3)
                 alpha=90           beta=90            gamma=90
Temperature:     110 K

                 Calculated                 Reported
Volume           2389.07(8)                 2389.07(7)
Space group      P 43                       P 43
Hall group       P 4cw                      P 43
Moiety formula   C34 H25 N                  C34 H25 N
Sum formula      C34 H25 N                  C34 H25 N
Mr               447.55                     447.55
Dx,g cm-3        1.244                      1.244
Z                4                          4
Mu (mm-1)        0.543                      0.543
F000             944.0                      944.0
F000'            946.45
h,k,lmax         17,17,15                   16,16,14
Nref             4605[ 2415]                3277
Tmin,Tmax        0.928,0.962                0.941,0.967
Tmin'            0.926

Correction method= # Reported T Limits: Tmin=0.941 Tmax=0.967
AbsCorr = GAUSSIAN

Data completeness= 1.36/0.71       Theta(max)= 70.990

R(reflections)= 0.0307( 3134)      wR2(reflections)= 0.0801( 3277)

S = 1.016                   Npar= 317
```

Selected bond lengths (Å) for **9**: C1-N1 = 1.375(2); C9-N1 = 1.314(2); Selected torsion angles (°) N1-C1-C10-C11 = -5.1(2); C4-C5-C16-C17 = -69.0(2). H-atoms omitted for clarity. Thermal ellipsoids set to 50%. Arbitrary numbering system used.

3.6. X-ray structure for complex 10

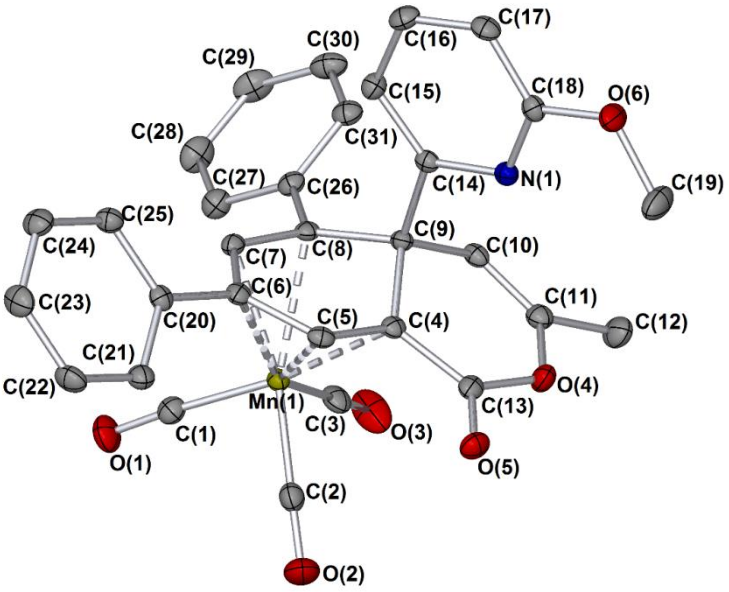

checkCIF/PLATON report

No syntax errors found.   CIF dictionary   Interpreting this report

Datablock: ijsf1512

```
Bond precision:   C-C = 0.0023 A          Wavelength=1.54184

Cell:              a=10.9613(2)     b=19.3911(3)       c=13.0063(3)
                   alpha=90         beta=110.468(3)    gamma=90
Temperature:       110 K

                   Calculated                 Reported
Volume             2589.98(10)                2589.97(10)
Space group        P 21/n                     P 1 21/n 1
Hall group         -P 2yn                     -P 2yn
Moiety formula     C31 H22 Mn N O6            C31 H22 Mn N O6
Sum formula        C31 H22 Mn N O6            C31 H22 Mn N O6
Mr                 559.44                     559.43
Dx,g cm-3          1.435                      1.435
Z                  4                          4
Mu (mm-1)          4.540                      4.540
F000               1152.0                     1152.0
F000'              1153.33
h,k,lmax           13,23,15                   13,23,15
Nref               5004                       4964
Tmin,Tmax          0.750,0.859                0.628,0.863
Tmin'              0.503

Correction method= # Reported T Limits: Tmin=0.628 Tmax=0.863
AbsCorr = GAUSSIAN

Data completeness= 0.992            Theta(max)= 71.014

R(reflections)= 0.0294( 4531)       wR2(reflections)= 0.0770( 4964)

S = 1.047                  Npar= 354
```

Selected bond lengths (Å) for **10**: C1-Mn1 = 1.8102(17); C2-Mn1 = 1.8176(18); C3-Mn1 = 1.8078(18); C4-Mn1 = 2.1401(15); C5-Mn1 = 2.1369(15); C6-Mn1 = 2.1842(15); C7-Mn1 = 2.1580(15); C8-Mn1 = 2.2843(15); C4-C5 = 1.414(2); C5-C6 = 1.406(2); C6-C7 = 1.433(2); C7-C8 = 1.399(2). Selected torsion angles (°) C26-C8-C9-C14 = 88.91(17); C7-C8-C26-C27 = -42.5(2). H-atoms omitted for clarity. Thermal ellipsoids set to 50%. Arbitrary numbering system used.

4. Computational studies (using DFT methods)

4.1. Computational details (general)

Initial optimizations were performed at the (RI-)BP86/SV(P) level, followed by frequency calculations at the same level. Transition states were located by initially performing a constrained minimization (by freezing internal coordinates that change most during the reaction) of a structure close to the anticipated transition state. This was followed by a frequency calculation to identify the transition vector to follow during a subsequent transition state optimization. A final frequency calculation was then performed on the optimized transition-state structure. All minima were confirmed as such by the absence of imaginary frequencies and all transition states were identified by the presence of only one imaginary frequency. Dynamic Reaction Coordinate analysis confirmed that transition states were connected to the appropriate minima. Energies, coordinates and first 50 vibrational modes are given.

Single-point calculations on the (RI-)BP86/SV(P) optimized geometries were performed using the hybrid PBE0 functional and the flexible def2-TZVPP basis set. The (RI-)PBE0/def2-TZVPP SCF energies were corrected for their zero point energies, thermal energies and entropies (obtained from the (RI-)BP86/SV(P)-level frequency calculations). No symmetry constraints were applied during optimisations. Solvent corrections were applied with the COSMO dielectric continuum model[1] and dispersion effects modelled with Grimme's D3 method.[2] All calculations were performed using the TURBOMOLE V6.4 package using the resolution of identity (RI) approximation.[3]

4.2. Collated theoretical data for the 2-pyrone system

Complex	bp86_SVP SCF energy (a.u.)	bp86_SVP Zero Point Energy (a.u.)	bp86_SVP Chem Pot (kJ mol^{-1})	D3-correction (a.u.)	pbe0_TZVVP_sp SCF energy (a.u.)	pbe0_TZVVP_sp SCF energy (a.u.) COSMO	Rel. H_sp (kJ mol-1) pbe0/TZVPP COSMO + D3	Rel. G_sp (kJ mol-1) pbe0/TZVPP COSMO + D3
IIPyr	-2427.867668	0.296291	622.29	-0.063245	-2427.429779	-2427.446217	0	0
TSIIPyr-IIIPyr	-2427.860924	0.295758	627.30	-0.063118	-2427.423481	-2427.438876	18	25
IIIPyr	-2427.897218	0.298154	630.17	-0.063511	-2427.461066	-2427.478036	-79	-76
TSIIIPyr-5g-iso	-2427.884704	0.297566	634.22	-0.063121	-2427.443250	-2427.456967	-25	-16
IVPyr	-2736.064250	0.407167	890.15	-0.088842	-2735.579777	-2735.599012		
Phenylacetylene	-308.169894	0.106495	199.25	-0.012246	-308.123235	-308.128881		
	-2427.894356	0.300672	690.90	-0.076596	-2427.456541	-2427.470131	-86	-29
5g-iso	-2427.903395	0.299612	640.50	-0.064133	-2427.468599	-2427.482434	-89	-79
5g	-2427.926735	0.300715	643.64	-0.064188	-2427.490967	-2427.507878	-153	-143
TSIVPyr-VPyr	-2736.049869	0.403330	877.02	-0.085375	-2735.567013	-2735.585294		
Phenylacetylene	-308.169894	0.106495	199.25	-0.012246	-308.123235	-308.128881		
	-2427.879975	0.296835	677.77	-0.073129	-2427.443778	-2427.456414	-51	3
VPyr	-2736.097341	0.408813	890.60	-0.085716	-2735.617112	-2735.637639		
Phenylacetylene	-308.169894	0.106495	199.25	-0.012246	-308.123235	-308.128881		
	-2427.927447	0.302319	691.35	-0.073471	-2427.493877	-2427.508759	-175	-122
TSIVPyr-VIPyr	-2736.057735	0.407292	896.60	-0.090587	-2735.569084	-2735.587041		
Phenylacetylene	-308.169894	0.106495	199.25	-0.012246	-308.123235	-308.128881		
	-2427.887841	0.300797	697.35	-0.078341	-2427.445849	-2427.458161	-59	4
VIPyr	-2736.163694	0.412209	915.98	-0.091155	-2735.685847	-2735.703075		
Phenylacetylene	-308.169894	0.106495	199.25	-0.012246	-308.123235	-308.128881		
	-2427.993800	0.305715	716.73	-0.078909	-2427.562612	-2427.574195	-352	-283
VIIa-Pyr	-2736.140442	0.411728	908.69	-0.083427	-2735.667640	-2735.687546		
Phenylacetylene	-308.169894	0.106495	199.25	-0.012246	-308.123235	-308.128881		
	-2427.970548	0.305233	709.44	-0.071182	-2427.544405	-2427.558665	-293	-229
VIIb-Pyr	-2736.139438	0.411563	906.29	-0.082841	-2735.666589	-2735.687342		
Phenylacetylene	-308.169894	0.106495	199.25	-0.012246	-308.123235	-308.128881		
	-2427.969544	0.305068	707.04	-0.070595	-2427.543354	-2427.558461	-291	-229
10pyr	-2736.173478	0.412531	914.40	-0.089458	-2735.701231	-2735.718203		
Phenylacetylene	-308.169894	0.106495	199.25	-0.012246	-308.123235	-308.128881		
	-2428.003584	0.306037	715.15	-0.077212	-2427.577995	-2427.589323	-387	-320

4.3. Collated theoretical data for the 2-phenylpyridine system

Complex	bp86_SVP SCF energy (a.u.)	bp86_SVP Zero Point Energy (a.u.)	bp86_SVP Chem Pot (kJ mol-1)	D3-correction (a.u.)	pbe0_TZVVP_sp SCF energy (a.u.)	pbe0_TZVVP_sp SCF energy (a.u.) COSMO	Rel. H_sp (kJ mol-1) pbe0/TZVPP COSMO + D3	Rel. E_sp (kJ mol-1) pbe0/TZVPP COSMO + D3
IIPh	-2277.516193	0.289763	615.48	-0.061216	-2277.070662	-2277.081458	0	0
TSIIPh-IIIPh	-2277.508411	0.289407	617.36	-0.060651	-2277.059172	-2277.069786	31	34
IIIPh	-2277.545461	0.291609	622.45	-0.064858	-2277.104427	-2277.116494	-97	-95
TSIIIPh-5b-iso	-2277.515270	0.290151	622.05	-0.061179	-2277.063594	-2277.073982	21	26
IVPh	-2585.712086	0.400435	882.18	-0.086779	-2585.222286	-2585.236061		
Phenylacetylene	-308.169894	0.106495	199.25	-0.012246	-308.123235	-308.128881		
	-2277.542192	0.293940	682.93	-0.074533	-2277.099050	-2277.107180	-92	-35
5b-iso	-2277.527970	0.291749	627.54	-0.062213	-2277.079674	-2277.089948	-20	-13
5b	-2277.555109	0.292969	631.14	-0.062246	-2277.110831	-2277.124961	-109	-101
TSIVPh-VPh	-2585.696976	0.396710	871.84	-0.083138	-2585.208684	-2585.222642		
Phenylacetylene	-308.169894	0.106495	199.25	-0.012246	-308.123235	-308.128881		
	-2277.527082	0.290215	672.59	-0.070892	-2277.085449	-2277.093762	-57	-1
VPh	-2585.746164	0.402203	882.64	-0.082414	-2585.259939	-2585.276133		
Phenylacetylene	-308.169894	0.106495	199.25	-0.012246	-308.123235	-308.128881		
	-2277.576270	0.295709	683.39	-0.070168	-2277.136703	-2277.147252	-181	-128
TSIVPh-VIPh	-2585.702784	0.400376	886.98	-0.087284	-2585.208439	-2585.208439		
Phenylacetylene	-308.169894	0.106495	199.25	-0.012246	-308.123235	-308.128881		
	-2277.532890	0.293881	687.73	-0.075039	-2277.085204	-2277.079558	-20	41
VIPh	-2585.785747	0.404435	904.30	-0.089202	-2585.296922	-2585.309870		
Phenylacetylene	-308.169894	0.106495	199.25	-0.012246	-308.123235	-308.128881		
	-2277.615853	0.297941	705.05	-0.076957	-2277.173687	-2277.180989	-281	-213
VIIa-Ph	-2585.761501	0.403700	896.52	-0.081777	-2585.277081	-2585.292363		
Phenylacetylene	-308.169894	0.106495	199.25	-0.012246	-308.123235	-308.128881		
	-2277.591607	0.297205	697.27	-0.069532	-2277.153845	-2277.163482	-218	-155
VIIb-Ph	-2585.760175	0.403573	894.01	-0.081005	-2585.275812	-2585.292041		
Phenylacetylene	-308.169894	0.106495	199.25	-0.012246	-308.123235	-308.128881		
	-2277.590281	0.297079	694.76	-0.068760	-2277.152577	-2277.163161	-215	-155
10Ph	-2585.788371	0.404172	899.32	-0.087320	-2585.304991	-2585.318945		
Phenylacetylene	-308.169894	0.106495	199.25	-0.012246	-308.123235	-308.128881		
	-2277.618477	0.297678	700.07	-0.075075	-2277.181756	-2277.190064	-301	-237

4.4. Output files

```
Complex IIPyr

SCF Energy (au) (RI)BP86/SV(P)      -2427.8676677770
SCF Energy (au) PBE0/def2-TZVPP     -2427.429779442
SCF Energy (au) PBE0/def2-TZVPP     -2427.4462170488 (Et2O Correction)
Zero Point Energy (au)              0.2962910
Chemical potential (kJ mol-1)             622.29
Dispersion correction (au) PBE0/def2-TZVPP      -0.06324515

xyz coordinates

43

Mn         1.46446        0.23441        0.59097
C          1.78164        0.44230        2.35075
C          3.15020       -0.37512        0.33314
C          1.99843        1.92979        0.22181
O          2.36015        3.02484        0.03931
O          4.25193       -0.67437        0.11842
O          1.98615        0.55607        3.49095
C         -1.36882       -0.18746        1.35292
C         -1.07313        1.97597        0.49946
C         -2.42542        2.29298        0.68036
C         -3.27740        1.31879        1.22670
C         -2.74158        0.07126        1.56306
C         -0.69651       -1.45218        1.66043
C          0.66383       -1.48522        1.36113
C          1.40812       -2.71559        1.61357
O          0.67061       -3.78957        2.20580
C         -0.65140       -3.72422        2.49559
C         -1.37280       -2.59063        2.23231
H         -0.37706        2.71764        0.07924
H         -2.79283        3.29104        0.39651
H         -4.34687        1.52972        1.38936
H         -3.38405       -0.71180        1.99126
O          2.57668       -2.94806        1.37133
C         -1.17278       -4.98959        3.10700
H         -2.44462       -2.57252        2.47549
N         -0.54559        0.77554        0.82329
C          0.85869        1.27286       -2.61429
C          1.97324        2.01859       -3.07750
C          1.80902        2.99940       -4.06484
C          0.53609        3.26319       -4.60250
C         -0.57635        2.53284       -4.14884
C         -0.42177        1.54669       -3.16341
H          2.97155        1.81351       -2.66102
H          2.68637        3.56582       -4.41811
H          0.41163        4.03814       -5.37665
H         -1.57663        2.73045       -4.56893
H         -1.29043        0.96761       -2.81215
C          1.00376        0.22568       -1.63193
C          0.97150       -0.92810       -1.11520
H          0.89564       -2.01094       -1.21801
H         -1.00973       -5.85221        2.42244
H         -2.25590       -4.90765        3.33029
H         -0.62804       -5.21985        4.05047
```

$vibrational spectrum

# mode	symmetry	wave number	IR intensity	selection rules	
#		cm**(-1)	km/mol	IR	RAMAN
1		0.00	0.00000	-	-
2		0.00	0.00000	-	-
3		0.00	0.00000	-	-
4		0.00	0.00000	-	-
5		0.00	0.00000	-	-
6		0.00	0.00000	-	-
7	a	12.81	0.33923	YES	YES
8	a	28.86	0.53659	YES	YES
9	a	32.34	0.18610	YES	YES
10	a	37.48	1.94999	YES	YES
11	a	60.15	0.05740	YES	YES
12	a	69.86	0.11553	YES	YES
13	a	75.56	0.07600	YES	YES
14	a	81.69	0.39010	YES	YES
15	a	91.77	1.65335	YES	YES
16	a	99.10	0.97354	YES	YES
17	a	109.91	0.13048	YES	YES
18	a	119.90	0.09960	YES	YES
19	a	123.51	1.49405	YES	YES
20	a	137.65	0.14122	YES	YES
21	a	148.66	1.93627	YES	YES
22	a	153.76	1.85356	YES	YES
23	a	159.62	1.33841	YES	YES
24	a	163.43	2.66110	YES	YES
25	a	192.44	1.04421	YES	YES
26	a	219.29	0.63095	YES	YES
27	a	249.37	0.36481	YES	YES
28	a	257.62	1.90206	YES	YES
29	a	294.76	2.37500	YES	YES
30	a	302.80	4.29265	YES	YES
31	a	322.18	10.77878	YES	YES
32	a	330.23	13.31044	YES	YES
33	a	398.33	4.09490	YES	YES
34	a	401.54	0.07919	YES	YES
35	a	414.56	12.50085	YES	YES
36	a	428.97	1.22915	YES	YES
37	a	455.68	0.76732	YES	YES
38	a	457.08	9.02848	YES	YES
39	a	467.25	5.30475	YES	YES
40	a	479.13	10.46195	YES	YES
41	a	486.47	4.00478	YES	YES
42	a	493.32	15.28063	YES	YES
43	a	500.80	11.85191	YES	YES
44	a	521.28	39.87042	YES	YES
45	a	532.34	6.18807	YES	YES
46	a	537.44	1.03557	YES	YES
47	a	544.61	5.84435	YES	YES
48	a	561.67	1.90348	YES	YES
49	a	581.57	3.54607	YES	YES
50	a	590.12	1.37823	YES	YES

TSIIPyr-IIIPyr

SCF Energy (au) (RI)BP86/SV(P) -2427.8609237480
SCF Energy (au) PBE0/def2-TZVPP -2427.423480510
SCF Energy (au) PBE0/def2-TZVPP -2427.4388763271 (Et2O Correction)
Zero Point Energy (au) 0.2957583
Chemical potential (kJ mol-1) 627.30
Dispersion correction (au) PBE0/def2-TZVPP -0.06311845

xyz coordinates

43

Mn	1.64726	0.59398	0.76884
C	2.01881	0.51463	2.52767
C	3.36440	0.21099	0.37051
C	2.05349	2.35298	0.65412
O	2.35172	3.48348	0.61670
O	4.48373	0.03264	0.11081
O	2.24636	0.45033	3.66771
C	-1.14073	-0.18580	1.38172
C	-0.99778	2.13966	1.10068
C	-2.37105	2.30349	1.30295
C	-3.15949	1.16306	1.54396
C	-2.53806	-0.08685	1.58269
C	-0.37685	-1.42689	1.38752
C	0.94890	-1.32986	0.92588
C	1.76917	-2.54818	0.93182
O	1.19526	-3.69805	1.54233
C	-0.08178	-3.75176	1.99859
C	-0.90818	-2.66399	1.88972
O	2.87937	-2.69181	0.45920
C	-0.42282	-5.06461	2.63314
N	-0.38792	0.93318	1.13256
C	0.16383	1.47881	-2.26847
C	0.52424	2.84658	-2.39683
C	0.02730	3.61760	-3.45771
C	-0.85610	3.05873	-4.39828
C	-1.24345	1.71169	-4.26801
C	-0.74563	0.92985	-3.21916
C	0.71226	0.60298	-1.26816
C	1.22026	-0.57302	-1.00816
H	-0.35167	3.00781	0.90210
H	-2.80988	3.31255	1.26641
H	-4.24752	1.25213	1.69520
H	-3.12918	-0.99794	1.75566
H	-1.93363	-2.73395	2.28096
H	1.22329	3.29469	-1.67571
H	0.33993	4.67082	-3.55337
H	-1.24168	3.66923	-5.23145
H	-1.93655	1.26184	-4.99860
H	-1.04232	-0.12678	-3.12311
H	1.73124	-1.45534	-1.40798
H	-1.48136	-5.08063	2.96353
H	0.22846	-5.24765	3.51856
H	-0.25012	-5.90618	1.92680

$vibrational spectrum

# mode	symmetry	wave number cm**(-1)	IR intensity km/mol	selection rules IR	RAMAN
1	a	-251.48	0.00000	YES	YES
2		0.00	0.00000	-	-
3		0.00	0.00000	-	-
4		0.00	0.00000	-	-
5		0.00	0.00000	-	-
6		0.00	0.00000	-	-
7		0.00	0.00000	-	-
8	a	28.22	1.17057	YES	YES
9	a	33.00	0.03669	YES	YES
10	a	37.31	0.25646	YES	YES
11	a	51.11	0.82999	YES	YES
12	a	66.48	0.46800	YES	YES
13	a	76.32	0.34264	YES	YES
14	a	82.58	0.46298	YES	YES
15	a	90.16	0.01570	YES	YES
16	a	94.98	0.39454	YES	YES
17	a	105.90	0.87215	YES	YES
18	a	112.79	0.63459	YES	YES
19	a	126.37	0.36234	YES	YES
20	a	138.43	0.42931	YES	YES
21	a	152.35	0.22464	YES	YES
22	a	157.00	0.16524	YES	YES
23	a	160.73	1.55604	YES	YES
24	a	176.53	0.78545	YES	YES
25	a	182.38	0.36980	YES	YES
26	a	216.93	0.32835	YES	YES
27	a	235.15	0.34588	YES	YES
28	a	252.41	0.60001	YES	YES
29	a	277.55	0.47099	YES	YES
30	a	299.41	1.61710	YES	YES
31	a	327.15	1.30911	YES	YES
32	a	362.89	13.21629	YES	YES
33	a	396.17	7.34509	YES	YES
34	a	401.54	0.18675	YES	YES
35	a	404.33	3.29136	YES	YES
36	a	426.10	2.51043	YES	YES
37	a	445.20	12.17383	YES	YES
38	a	453.41	0.87244	YES	YES
39	a	469.40	8.55667	YES	YES
40	a	482.52	2.58811	YES	YES
41	a	488.32	1.80246	YES	YES
42	a	497.18	2.19032	YES	YES
43	a	521.37	1.27760	YES	YES
44	a	534.47	14.18521	YES	YES
45	a	540.96	1.79099	YES	YES
46	a	543.75	14.00749	YES	YES
47	a	555.00	4.79139	YES	YES
48	a	558.93	13.19272	YES	YES
49	a	574.96	3.86009	YES	YES
50	a	595.67	0.27966	YES	YES

Complex IIIPyr

SCF Energy (au) (RI)BP86/SV(P) -2427.8972184830
SCF Energy (au) PBE0/def2-TZVPP -2427.461065744
SCF Energy (au) PBE0/def2-TZVPP -2427.4780357630 (Et2O Correction)
Zero Point Energy (au) 0.2981535
Chemical potential (kJ mol-1) 630.17
Dispersion correction (au) PBE0/def2-TZVPP -0.06351141

xyz coordinates

43

Mn	1.1809113	0.7160361	0.5661925
C	1.6599539	0.9218201	2.3219125
C	2.8123354	0.1037211	0.0885910
C	1.6282779	2.3716487	0.1742881
O	1.8906134	3.4875925	-0.0648879
O	3.8663191	-0.2681818	-0.2372804
O	1.9974224	1.0637984	3.4300767
C	-1.3640724	0.0005262	1.3597146
C	-1.5345914	2.2324693	0.6749420
C	-2.9000201	2.2631839	0.9893543
C	-3.5043102	1.1128062	1.5226791
C	-2.7224340	-0.0386217	1.7133387
C	-0.4324317	-1.1555155	1.4903419
C	0.2279847	-1.6851839	0.3593969
C	0.9518467	-2.9703330	0.5090206
O	1.0599404	-3.4894956	1.8238075
C	0.4520783	-2.9346854	2.9012808
C	-0.3119855	-1.8038792	2.7700864
H	-1.0106710	3.1112429	0.2665884
H	-3.4764497	3.1857433	0.8190183
H	-4.5749250	1.1093858	1.7832453
H	-3.1549943	-0.9671792	2.1170725
O	1.4403514	-3.6214074	-0.3850047
C	0.7252989	-3.6794327	4.1698190
H	-0.7990394	-1.3708116	3.6556910
N	-0.7870657	1.1260454	0.8684271
C	0.4305926	0.7441444	-2.5882364
C	1.4330985	1.6350667	-3.0503685
C	1.3607510	2.2088936	-4.3280751
C	0.2737338	1.9253415	-5.1742614
C	-0.7384592	1.0548140	-4.7308702
C	-0.6599411	0.4700766	-3.4587716
H	2.2922787	1.8612718	-2.4000015
H	2.1610983	2.8884691	-4.6654704
H	0.2129649	2.3857528	-6.1744208
H	-1.5993220	0.8310096	-5.3833706
H	-1.4627085	-0.2013660	-3.1118387
C	0.5016073	0.1161480	-1.2647488
C	0.1300807	-1.1754710	-1.0246468
H	-0.0775249	-1.9217095	-1.8187820
H	0.1925892	-3.2177868	5.0252863
H	1.8173501	-3.6829787	4.3869423
H	0.4114673	-4.7429690	4.0739221

$vibrational spectrum

# mode	symmetry	wave number	IR intensity	selection rules	
#		cm**(-1)	km/mol	IR	RAMAN
1		0.00	0.00000	-	-
2		0.00	0.00000	-	-
3		0.00	0.00000	-	-
4		0.00	0.00000	-	-
5		0.00	0.00000	-	-
6		0.00	0.00000	-	-
7	a	27.12	0.22935	YES	YES
8	a	31.29	1.16048	YES	YES
9	a	38.64	0.09071	YES	YES
10	a	47.02	3.23047	YES	YES
11	a	51.05	0.03429	YES	YES
12	a	65.82	0.30968	YES	YES
13	a	70.64	0.62343	YES	YES
14	a	83.70	0.73055	YES	YES
15	a	85.74	0.29203	YES	YES
16	a	98.68	0.91363	YES	YES
17	a	100.41	0.85824	YES	YES
18	a	105.59	0.32141	YES	YES
19	a	121.98	0.32566	YES	YES
20	a	142.14	0.20940	YES	YES
21	a	147.66	1.31771	YES	YES
22	a	154.68	0.60493	YES	YES
23	a	164.90	0.62170	YES	YES
24	a	168.58	1.01892	YES	YES
25	a	204.04	1.34280	YES	YES
26	a	234.09	0.82967	YES	YES
27	a	264.90	0.57888	YES	YES
28	a	271.62	0.42112	YES	YES
29	a	287.10	2.49699	YES	YES
30	a	306.14	0.94492	YES	YES
31	a	337.11	1.05493	YES	YES
32	a	370.69	2.97909	YES	YES
33	a	395.16	8.91538	YES	YES
34	a	408.26	0.68248	YES	YES
35	a	432.08	0.64144	YES	YES
36	a	450.93	2.20257	YES	YES
37	a	458.62	0.68395	YES	YES
38	a	471.99	2.82907	YES	YES
39	a	480.60	1.00634	YES	YES
40	a	486.93	0.72458	YES	YES
41	a	495.73	7.17870	YES	YES
42	a	498.30	3.05798	YES	YES
43	a	520.61	3.47501	YES	YES
44	a	534.58	2.34983	YES	YES
45	a	543.15	2.48004	YES	YES
46	a	552.57	3.13271	YES	YES
47	a	564.02	1.10868	YES	YES
48	a	599.28	2.19912	YES	YES
49	a	613.24	1.04429	YES	YES
50	a	616.73	25.68202	YES	YES

Complex TSIIIPyr-5g-iso

SCF Energy (au) (RI)BP86/SV(P) -2427.8847042340
SCF Energy (au) PBE0/def2-TZVPP -2427.443249535
SCF Energy (au) PBE0/def2-TZVPP -2427.4569672510 (Et2O Correction)
Zero Point Energy (au) 0.2975664
Chemical potential (kJ mol-1) 634.22
Dispersion correction (au) PBE0/def2-TZVPP -0.06312078

xyz coordinates

43

C	2.12240	0.16838	2.70822
C	-0.01254	1.43094	2.90018
C	0.59034	1.08733	0.08708
C	-0.10502	-1.20217	2.96829
C	1.11235	-1.35947	0.24079
C	2.29497	-2.25402	0.37532
C	0.93665	-4.09689	-0.46608
C	-0.15982	-3.29834	-0.65365
H	-1.08094	-3.75481	-1.04498
C	-0.14379	-1.92663	-0.23956
C	-1.24091	-1.04334	-0.27172
C	-2.44014	-1.21525	-1.03195
H	-2.52317	-2.09006	-1.69564
C	-3.46605	-0.29655	-0.94237
H	-4.38960	-0.42235	-1.52919
C	-3.30228	0.83577	-0.08009
H	-4.11172	1.57045	0.05147
C	-2.12021	1.01330	0.59913
H	-1.94766	1.88708	1.24475
C	1.03522	-5.56108	-0.75338
H	1.86846	-5.76798	-1.46307
H	0.08971	-5.94568	-1.18597
H	1.25962	-6.12901	0.17863
Mn	0.53328	0.08675	1.86414
N	-1.05900	0.12399	0.50786
O	3.13765	0.24028	3.27363
O	-0.35165	2.30347	3.60252
C	1.46904	0.03152	-0.05637
O	-0.55096	-2.02270	3.66318
O	3.40805	-1.90623	0.70574
O	2.10652	-3.59995	0.05839
H	2.53469	0.22381	-0.27993
C	0.52088	2.40824	-0.50245
C	0.33144	4.96385	-1.71730
C	0.41654	4.85041	-0.31833
C	0.49537	3.58865	0.28570
C	0.44146	2.53904	-1.91877
C	0.34202	3.80251	-2.51506
H	0.25835	5.95821	-2.18766
H	0.42061	5.75665	0.31051
H	0.55860	3.50642	1.38163
H	0.44923	1.63026	-2.54198
H	0.27200	3.88522	-3.61168

```
$vibrational spectrum
#  mode     symmetry     wave number   IR intensity    selection rules
#                         cm**(-1)        km/mol         IR     RAMAN
     1        a           -313.22         0.00000        YES     YES
     2                       0.00         0.00000         -       -
     3                       0.00         0.00000         -       -
     4                       0.00         0.00000         -       -
     5                       0.00         0.00000         -       -
     6                       0.00         0.00000         -       -
     7                       0.00         0.00000         -       -
     8        a             27.61         0.11069        YES     YES
     9        a             38.62         0.41871        YES     YES
    10        a             43.17         0.54374        YES     YES
    11        a             48.78         0.86531        YES     YES
    12        a             64.29         0.13221        YES     YES
    13        a             74.25         0.36216        YES     YES
    14        a             83.34         0.18100        YES     YES
    15        a             89.22         0.03585        YES     YES
    16        a             98.52         0.48637        YES     YES
    17        a            106.84         0.55289        YES     YES
    18        a            108.86         1.00880        YES     YES
    19        a            136.58         0.78659        YES     YES
    20        a            144.70         0.04722        YES     YES
    21        a            150.27         0.29127        YES     YES
    22        a            155.44         0.16389        YES     YES
    23        a            181.27         1.19437        YES     YES
    24        a            202.68         1.02325        YES     YES
    25        a            211.55         0.43202        YES     YES
    26        a            230.55         2.09321        YES     YES
    27        a            235.36         0.65385        YES     YES
    28        a            281.24         0.53096        YES     YES
    29        a            300.84         2.02590        YES     YES
    30        a            310.91         3.68670        YES     YES
    31        a            330.35         2.73179        YES     YES
    32        a            384.22         4.89720        YES     YES
    33        a            401.40         0.20435        YES     YES
    34        a            405.67         3.85013        YES     YES
    35        a            425.87         0.66141        YES     YES
    36        a            439.66         0.70581        YES     YES
    37        a            462.46         5.17513        YES     YES
    38        a            473.46         0.53569        YES     YES
    39        a            476.17         0.19537        YES     YES
    40        a            484.77         1.73829        YES     YES
    41        a            507.36         2.81418        YES     YES
    42        a            511.77         0.97991        YES     YES
    43        a            526.51         8.28956        YES     YES
    44        a            530.73        10.26720        YES     YES
    45        a            540.44         1.15894        YES     YES
    46        a            549.58         0.07862        YES     YES
    47        a            563.84         9.50874        YES     YES
    48        a            592.11         3.71291        YES     YES
    49        a            603.58         4.26946        YES     YES
    50        a            614.29         4.27704        YES     YES
```

Complex IVPyr

SCF Energy (au) (RI)BP86/SV(P) -2736.0642498080
SCF Energy (au) PBE0/def2-TZVPP -2735.579776606
SCF Energy (au) PBE0/def2-TZVPP -2735.5990118420 (Et2O Correction)
Zero Point Energy (au) 0.4071670
Chemical potential (kJ mol-1) 890.15
Dispersion correction (au) PBE0/def2-TZVPP -0.08884194

xyz coordinates

57

Mn	0.48425	1.38796	-0.81986
C	1.32345	2.64097	0.19515
C	-0.55595	2.68993	-1.51244
O	-1.17056	3.57669	-1.94968
O	1.83934	3.51294	0.77338
C	-1.35337	-0.13224	1.36087
C	-1.54754	2.18593	1.28068
C	-2.45252	2.22636	2.34278
C	-2.81794	1.01783	2.95426
C	-2.25682	-0.15833	2.45635
C	-0.89286	-1.45972	0.87628
C	-1.01740	-1.86752	-0.45385
C	-0.86879	-3.30594	-0.77313
O	-0.47324	-4.15709	0.28593
C	-0.26321	-3.72210	1.55223
C	-0.48908	-2.41217	1.88133
H	-1.24149	3.12650	0.80217
H	-2.85370	3.19562	2.67714
H	-3.53334	0.99075	3.79223
H	-2.53831	-1.13363	2.88069
O	-1.04548	-3.82773	-1.85402
C	0.21815	-4.80400	2.46840
H	-0.31321	-2.08397	2.91586
N	-0.99008	1.04894	0.78295
C	-1.36473	0.66588	-3.30776
C	-0.52603	0.99537	-4.40095
C	-1.06325	1.40741	-5.63018
C	-2.45423	1.52536	-5.79724
C	-3.30337	1.21204	-4.72264
C	-2.76608	0.78142	-3.49931
H	0.56501	0.90002	-4.29841
H	-0.38430	1.63855	-6.46812
H	-2.87351	1.86087	-6.76033
H	-4.39703	1.30210	-4.83621
H	-3.43890	0.54029	-2.65951
C	-0.83509	0.18296	-1.99803
C	-1.32232	-1.04533	-1.61748
H	-1.91345	-1.59949	-2.37415
C	2.75603	0.29585	1.56471
C	2.25721	0.89519	2.74854
C	3.06221	0.99054	3.89239
C	4.37960	0.49894	3.87836
C	4.88902	-0.09236	2.70803
C	4.09007	-0.19378	1.56122
H	1.22806	1.28548	2.76490
H	2.65761	1.45925	4.80469
H	5.01153	0.58161	4.77784
H	5.92276	-0.47526	2.68580
H	4.48847	-0.65226	0.64240
C	1.95464	0.11952	0.37754
C	1.52068	-0.45498	-0.66107

```
H          1.44252      -1.29723      -1.34697
C          1.68217       1.63111      -2.11560
O          2.50677       1.79235      -2.92560
H          0.36359      -4.41770       3.49734
H          1.18294      -5.22291       2.10298
H         -0.50888      -5.64677       2.49713

$vibrational spectrum
#  mode     symmetry     wave number   IR intensity    selection rules
#                         cm**(-1)        km/mol          IR     RAMAN
     1                       0.00         0.00000         -        -
     2                       0.00         0.00000         -        -
     3                       0.00         0.00000         -        -
     4                       0.00         0.00000         -        -
     5                       0.00         0.00000         -        -
     6                       0.00         0.00000         -        -
     7         a            15.28         0.08503        YES      YES
     8         a            23.94         0.24908        YES      YES
     9         a            24.79         0.93086        YES      YES
    10         a            29.46         1.14666        YES      YES
    11         a            35.46         0.42024        YES      YES
    12         a            39.76         1.40762        YES      YES
    13         a            54.81         0.25312        YES      YES
    14         a            59.28         0.74567        YES      YES
    15         a            62.84         0.34089        YES      YES
    16         a            78.14         0.08535        YES      YES
    17         a            89.22         0.45727        YES      YES
    18         a           100.65         0.30226        YES      YES
    19         a           101.66         0.75847        YES      YES
    20         a           106.42         0.43450        YES      YES
    21         a           114.13         0.68926        YES      YES
    22         a           124.17         1.12107        YES      YES
    23         a           129.97         0.78231        YES      YES
    24         a           136.58         0.14382        YES      YES
    25         a           142.46         0.92835        YES      YES
    26         a           151.46         0.60214        YES      YES
    27         a           154.43         0.84924        YES      YES
    28         a           163.13         0.61444        YES      YES
    29         a           184.27         2.47388        YES      YES
    30         a           190.06         0.46612        YES      YES
    31         a           202.60         2.66689        YES      YES
    32         a           221.21         1.18430        YES      YES
    33         a           235.81         0.66283        YES      YES
    34         a           255.54         0.40546        YES      YES
    35         a           274.31         0.30423        YES      YES
    36         a           283.33         2.33946        YES      YES
    37         a           310.71         2.09700        YES      YES
    38         a           322.59        18.89716        YES      YES
    39         a           352.00         1.62840        YES      YES
    40         a           397.52         1.62831        YES      YES
    41         a           398.17         1.09791        YES      YES
    42         a           403.17         1.71778        YES      YES
    43         a           404.51         3.00637        YES      YES
    44         a           431.66         0.19756        YES      YES
    45         a           442.39         1.91944        YES      YES
    46         a           461.42         4.52365        YES      YES
    47         a           469.15         0.24575        YES      YES
    48         a           470.13         2.66061        YES      YES
    49         a           477.24         1.57467        YES      YES
    50         a           486.29         3.77694        YES      YES
```

Complex 5g-iso

SCF Energy (au) (RI)BP86/SV(P) -2427.9033946000
SCF Energy (au) PBE0/def2-TZVPP -2427.468599
SCF Energy (au) PBE0/def2-TZVPP -2427.482434(Et2O Correction)
Zero Point Energy (au) 0.2996119
Chemical potential (kJ mol-1) 640.50
Dispersion correction (au) PBE0/def2-TZVPP -0.064133

xyz coordinates

43

C	-0.21734	-0.48354	3.36140
C	-1.71340	1.21180	2.37873
C	0.34445	0.87774	0.32012
C	-2.17172	-1.38215	1.81652
C	0.59214	-1.53233	0.63088
C	1.30189	-2.61087	1.35561
C	0.79486	-4.11051	-0.49978
C	0.20424	-3.12815	-1.24561
H	-0.17451	-3.38721	-2.24526
C	0.00666	-1.81467	-0.70001
C	-0.70193	-0.77483	-1.27510
C	-1.33479	-0.72983	-2.55750
H	-1.13500	-1.56082	-3.25211
C	-2.19819	0.28152	-2.90802
H	-2.68717	0.29119	-3.89397
C	-2.47531	1.33026	-1.94893
H	-3.22612	2.10511	-2.16839
C	-1.81456	1.38720	-0.76228
H	-1.96277	2.18411	-0.02184
C	0.99029	-5.53470	-0.91354
H	2.07037	-5.80516	-0.88671
H	0.59968	-5.71156	-1.93603
H	0.47074	-6.22067	-0.20620
Mn	-0.81664	-0.17972	1.69783
N	-0.84541	0.40004	-0.38698
O	0.15577	-0.66438	4.44945
O	-2.29425	2.11312	2.84426
C	1.09063	-0.18734	0.86495
O	-3.04042	-2.15326	1.87565
O	1.91773	-2.48667	2.39197
O	1.28133	-3.88279	0.76763
H	2.01987	-0.00561	1.42499
C	0.79483	2.29046	0.24432
C	1.76809	4.93993	0.07971
C	1.71531	4.28197	1.32184
C	1.22614	2.97029	1.40580
C	0.85673	2.95646	-1.00240
C	1.33680	4.27372	-1.07986
H	2.14389	5.97439	0.01695
H	2.04581	4.79944	2.23736
H	1.15753	2.46460	2.38280
H	0.54309	2.43332	-1.91999
H	1.38064	4.78007	-2.05827

$vibrational spectrum

# mode	symmetry	wave number cm**(-1)	IR intensity km/mol	selection rules IR	selection rules RAMAN
1		0.00	0.00000	-	-
2		0.00	0.00000	-	-
3		0.00	0.00000	-	-
4		0.00	0.00000	-	-
5		0.00	0.00000	-	-
6		0.00	0.00000	-	-
7	a	38.34	1.09833	YES	YES
8	a	43.28	0.14448	YES	YES
9	a	43.44	0.13144	YES	YES
10	a	50.94	0.22010	YES	YES
11	a	58.19	0.26832	YES	YES
12	a	72.39	0.38384	YES	YES
13	a	86.10	0.37624	YES	YES
14	a	86.92	0.27807	YES	YES
15	a	96.11	0.99971	YES	YES
16	a	107.58	0.33120	YES	YES
17	a	110.57	0.76486	YES	YES
18	a	138.89	0.18969	YES	YES
19	a	141.27	1.11699	YES	YES
20	a	152.20	0.25306	YES	YES
21	a	162.06	0.40674	YES	YES
22	a	172.34	0.81685	YES	YES
23	a	201.88	0.54105	YES	YES
24	a	216.95	0.53601	YES	YES
25	a	231.60	0.86341	YES	YES
26	a	243.48	0.13934	YES	YES
27	a	275.06	3.59804	YES	YES
28	a	307.64	1.10648	YES	YES
29	a	325.63	1.92340	YES	YES
30	a	335.89	0.82541	YES	YES
31	a	395.53	0.99810	YES	YES
32	a	403.98	0.39787	YES	YES
33	a	414.94	6.05032	YES	YES
34	a	426.60	1.99447	YES	YES
35	a	438.02	1.18876	YES	YES
36	a	458.62	8.25551	YES	YES
37	a	466.74	2.62942	YES	YES
38	a	470.47	3.41281	YES	YES
39	a	475.96	0.38939	YES	YES
40	a	486.74	9.29193	YES	YES
41	a	512.15	5.98080	YES	YES
42	a	519.24	11.46393	YES	YES
43	a	525.26	5.23306	YES	YES
44	a	533.01	8.77495	YES	YES
45	a	540.42	5.80861	YES	YES
46	a	563.56	0.42122	YES	YES
47	a	593.55	2.45888	YES	YES
48	a	598.67	39.26678	YES	YES
49	a	606.32	2.81781	YES	YES
50	a	613.58	3.49195	YES	YES

Complex 5g

SCF Energy (au) (RI)BP86/SV(P) -2427.9267352960
SCF Energy (au) PBE0/def2-TZVPP -2427.490966955
SCF Energy (au) PBE0/def2-TZVPP -2427.5078781002 (Et2O Correction)
Zero Point Energy (au) 0.3007148
Chemical potential (kJ mol-1) 643.64
Dispersion correction (au) PBE0/def2-TZVPP -0.06418794

xyz coordinates

43

C	2.13012	1.43496	1.57423
C	-0.25647	0.55327	2.42890
C	0.05814	0.88022	-0.45591
C	1.98462	-0.91722	2.44079
C	1.25372	-1.21993	-0.36403
C	2.41011	-2.12279	-0.57687
C	1.23393	-3.77094	0.79068
C	0.11205	-3.01154	0.91153
H	-0.71428	-3.39032	1.53210
C	0.03194	-1.68253	0.30716
C	-1.22681	-1.17313	-0.22049
C	-2.42835	-1.90520	-0.33589
H	-2.42998	-2.95379	-0.00761
C	-3.57948	-1.31306	-0.86524
H	-4.51817	-1.88696	-0.92683
C	-3.51868	0.01155	-1.33498
H	-4.39313	0.51742	-1.76979
C	-2.30971	0.70096	-1.23833
H	-2.18526	1.73698	-1.58295
C	1.48706	-5.09951	1.43181
H	1.75217	-5.86264	0.66600
H	0.59511	-5.44452	1.99290
H	2.34681	-5.03155	2.13657
Mn	1.02393	0.05994	1.26969
N	-1.21371	0.13649	-0.66847
O	2.84767	2.33576	1.77064
O	-1.11155	0.88750	3.15386
C	1.25632	0.12701	-0.79914
O	2.58888	-1.55531	3.21033
O	3.39370	-1.86203	-1.23442
O	2.32163	-3.36801	0.03138
H	2.13031	0.56145	-1.30562
C	-0.01916	2.34680	-0.75707
C	-0.15588	5.12324	-1.32540
C	0.48989	4.25160	-2.21704
C	0.55009	2.87505	-1.93890
C	-0.66417	3.23875	0.13291
C	-0.73690	4.61044	-0.15117
H	-0.20156	6.20359	-1.54060
H	0.95087	4.64294	-3.13903
H	1.04816	2.19904	-2.65341
H	-1.09871	2.84738	1.06670
H	-1.23527	5.28865	0.56101

$vibrational spectrum

# mode	symmetry	wave number	IR intensity	selection rules	
#		cm**(-1)	km/mol	IR	RAMAN
1		0.00	0.00000	-	-
2		0.00	0.00000	-	-
3		0.00	0.00000	-	-
4		0.00	0.00000	-	-
5		0.00	0.00000	-	-
6		0.00	0.00000	-	-
7	a	30.49	0.37368	YES	YES
8	a	41.17	0.05719	YES	YES
9	a	50.34	0.92509	YES	YES
10	a	57.34	0.68510	YES	YES
11	a	63.97	1.56357	YES	YES
12	a	70.93	0.14406	YES	YES
13	a	72.84	0.43898	YES	YES
14	a	87.73	0.44337	YES	YES
15	a	90.68	0.58659	YES	YES
16	a	103.45	0.11898	YES	YES
17	a	105.31	0.44689	YES	YES
18	a	118.96	0.91408	YES	YES
19	a	147.07	0.78987	YES	YES
20	a	152.08	3.11613	YES	YES
21	a	173.84	0.57882	YES	YES
22	a	188.11	3.01119	YES	YES
23	a	219.29	1.00079	YES	YES
24	a	221.90	0.71666	YES	YES
25	a	232.64	1.41315	YES	YES
26	a	253.63	0.93085	YES	YES
27	a	275.58	0.31810	YES	YES
28	a	306.90	2.10092	YES	YES
29	a	324.40	1.94840	YES	YES
30	a	400.74	6.67914	YES	YES
31	a	404.26	0.17224	YES	YES
32	a	414.33	3.44676	YES	YES
33	a	423.47	6.91811	YES	YES
34	a	425.29	0.50748	YES	YES
35	a	446.33	1.99700	YES	YES
36	a	463.66	5.00880	YES	YES
37	a	473.78	5.34276	YES	YES
38	a	480.07	4.04470	YES	YES
39	a	492.65	0.47106	YES	YES
40	a	499.36	0.96342	YES	YES
41	a	509.02	7.28792	YES	YES
42	a	517.91	2.90610	YES	YES
43	a	529.92	7.57256	YES	YES
44	a	537.67	5.09718	YES	YES
45	a	549.19	3.32295	YES	YES
46	a	566.39	3.79022	YES	YES
47	a	587.15	6.07669	YES	YES
48	a	597.50	47.02940	YES	YES
49	a	608.98	4.63128	YES	YES
50	a	616.67	44.44609	YES	YES

Complex TSIVPyr-VPyr

SCF Energy (au) (RI)BP86/SV(P) -2736.0498688870
SCF Energy (au) PBE0/def2-TZVPP -2735.567012846
SCF Energy (au) PBE0/def2-TZVPP -2735.5852941514 (Et2O Correction)
Zero Point Energy (au) 0.4033298
Chemical potential (kJ mol-1) 877.02
Dispersion correction (au) PBE0/def2-TZVPP -0.08537481

xyz coordinates

57

Mn	0.11831	2.12227	-0.41495
C	1.13309	3.10106	0.72404
C	-0.93817	3.49169	-0.95149
O	-1.57635	4.39094	-1.32810
O	1.80825	3.69030	1.46618
C	-1.52363	0.54934	1.85835
C	-1.72906	2.86630	1.84109
C	-2.55417	2.88101	2.96629
C	-2.88155	1.65933	3.57085
C	-2.35359	0.49670	3.00890
C	-1.07981	-0.77808	1.34441
C	-1.33545	-1.22421	0.04783
C	-1.22895	-2.66880	-0.25124
O	-0.70851	-3.48941	0.77642
C	-0.36585	-3.02083	1.99969
C	-0.57138	-1.70587	2.32402
H	-1.45671	3.81766	1.36482
H	-2.92515	3.84293	3.35262
H	-3.53774	1.61139	4.45499
H	-2.59875	-0.48858	3.43250
O	-1.54396	-3.21759	-1.28577
C	0.23002	-4.07311	2.88136
H	-0.29574	-1.35263	3.32784
N	-1.21236	1.74157	1.27398
C	-1.52601	1.23112	-2.89031
C	-0.61792	1.26641	-3.97545
C	-1.03574	1.67871	-5.25009
C	-2.36639	2.07670	-5.46925
C	-3.27908	2.04713	-4.40152
C	-2.86530	1.62387	-3.12811
H	0.42422	0.94286	-3.82434
H	-0.31198	1.68530	-6.08209
H	-2.69052	2.40754	-6.46978
H	-4.32637	2.35544	-4.55923
H	-3.58530	1.60390	-2.29314
C	-1.12580	0.74843	-1.53966
C	-1.70143	-0.42417	-1.11680
H	-2.35782	-0.95685	-1.83234
C	3.20804	-1.24260	0.58103
C	3.76623	-1.27172	1.88918
C	4.77264	-2.19099	2.21443
C	5.24077	-3.10482	1.25228
C	4.69479	-3.08935	-0.04429
C	3.69313	-2.16932	-0.38227
H	3.40171	-0.55423	2.64139
H	5.19922	-2.19386	3.23152
H	6.03277	-3.82661	1.51165
H	5.05700	-3.80125	-0.80454
H	3.26934	-2.15505	-1.39874
C	2.18918	-0.30293	0.24378
C	1.29176	0.51817	-0.01802

H	0.26178	0.49618	-0.87150
C	1.24321	2.55030	-1.74221
O	2.00598	2.87385	-2.56086
H	0.44924	-3.66895	3.88998
H	1.17626	-4.45589	2.43714
H	-0.46040	-4.94067	2.97751

$vibrational spectrum

# mode	symmetry	wave number	IR intensity	selection rules	
#		cm**(-1)	km/mol	IR	RAMAN
1	a	-711.52	0.00000	YES	YES
2		0.00	0.00000	-	-
3		0.00	0.00000	-	-
4		0.00	0.00000	-	-
5		0.00	0.00000	-	-
6		0.00	0.00000	-	-
7		0.00	0.00000	-	-
8	a	14.45	0.03892	YES	YES
9	a	17.00	0.08140	YES	YES
10	a	20.04	0.62579	YES	YES
11	a	24.48	1.08358	YES	YES
12	a	32.43	0.99382	YES	YES
13	a	39.15	1.97367	YES	YES
14	a	52.32	0.20032	YES	YES
15	a	57.17	1.15702	YES	YES
16	a	63.57	0.57024	YES	YES
17	a	68.83	0.12564	YES	YES
18	a	83.57	0.07608	YES	YES
19	a	90.49	0.96283	YES	YES
20	a	96.46	1.13427	YES	YES
21	a	99.12	0.03013	YES	YES
22	a	109.00	0.46587	YES	YES
23	a	111.33	0.53683	YES	YES
24	a	118.97	0.29369	YES	YES
25	a	132.60	2.36029	YES	YES
26	a	138.86	0.42275	YES	YES
27	a	150.83	0.62600	YES	YES
28	a	161.01	3.14800	YES	YES
29	a	168.23	2.41929	YES	YES
30	a	189.65	0.36157	YES	YES
31	a	198.90	5.31614	YES	YES
32	a	216.51	0.34879	YES	YES
33	a	222.59	5.69584	YES	YES
34	a	255.46	0.30743	YES	YES
35	a	274.58	0.12852	YES	YES
36	a	280.55	5.70704	YES	YES
37	a	282.31	10.18983	YES	YES
38	a	304.52	12.47405	YES	YES
39	a	312.49	0.46789	YES	YES
40	a	355.39	10.05891	YES	YES
41	a	398.24	1.74557	YES	YES
42	a	399.59	4.02823	YES	YES
43	a	400.99	0.24438	YES	YES
44	a	403.25	11.13414	YES	YES
45	a	434.02	4.27028	YES	YES
46	a	452.76	8.09673	YES	YES
47	a	469.59	4.15181	YES	YES
48	a	475.63	13.57671	YES	YES
49	a	476.07	0.59738	YES	YES
50	a	480.82	1.28670	YES	YES

Complex VPyr

SCF Energy (au) (RI)BP86/SV(P) -2736.0973407470
SCF Energy (au) PBE0/def2-TZVPP -2735.617112111
SCF Energy (au) PBE0/def2-TZVPP -2735.6376391368 (Et2O Correction)
Zero Point Energy (au) 0.4088133
Chemical potential (kJ mol-1) 890.60
Dispersion correction (au) PBE0/def2-TZVPP -0.08571642

xyz coordinates

57

Mn	0.05988	1.89438	-0.29883
C	1.08198	2.96501	0.69842
C	-1.04758	3.23725	-0.83574
O	-1.68695	4.16286	-1.15227
O	1.76896	3.64468	1.34837
C	-1.68456	0.41128	1.97077
C	-1.58337	2.73196	2.14772
C	-2.41819	2.75128	3.27096
C	-2.93041	1.53634	3.74583
C	-2.55431	0.36301	3.08538
C	-1.25758	-0.85501	1.32981
C	-1.06368	-0.92194	-0.04734
C	-0.51228	-2.15086	-0.63842
O	-0.31558	-3.22818	0.26021
C	-0.49709	-3.14384	1.59581
C	-0.97944	-1.99011	2.16077
H	-1.18676	3.67635	1.74786
H	-2.66111	3.71270	3.74924
H	-3.61164	1.50157	4.61146
H	-2.94723	-0.61166	3.41178
O	-0.21797	-2.35698	-1.79792
C	-0.09482	-4.38933	2.31996
H	-1.06643	-1.92599	3.25411
N	-1.21680	1.60520	1.49189
C	-1.39465	1.18586	-3.29464
C	-0.63426	1.29089	-4.48650
C	-1.14225	1.95329	-5.61320
C	-2.42633	2.52288	-5.57497
C	-3.19838	2.42085	-4.40232
C	-2.69041	1.76022	-3.27667
H	0.37254	0.84419	-4.52061
H	-0.53169	2.02297	-6.52831
H	-2.82992	3.04262	-6.45965
H	-4.20971	2.85845	-4.36807
H	-3.31708	1.68133	-2.37428
C	-0.82003	0.43752	-2.16264
C	-1.41731	0.20242	-0.92849
H	-2.38378	0.68781	-0.70346
C	3.24041	-1.24520	0.98114
C	4.05363	-1.05408	2.13331
C	5.02859	-1.99620	2.49010
C	5.22265	-3.15278	1.71216
C	4.42858	-3.35658	0.56819
C	3.45089	-2.41966	0.20394
H	3.90721	-0.14709	2.74152
H	5.64928	-1.82445	3.38590
H	5.99297	-3.89001	1.99340
H	4.57594	-4.25611	-0.05332
H	2.83348	-2.57937	-0.69451
C	2.24448	-0.28937	0.61063
C	1.34452	0.50356	0.27795

```
H          0.06863       -0.16381       -2.40550
C          1.19049        2.15441       -1.66095
O          1.96247        2.33972       -2.51308
H         -0.30568       -4.30486        3.40485
H          0.99339       -4.57799        2.17828
H         -0.63576       -5.27140        1.91000

$vibrational spectrum
#  mode     symmetry     wave number   IR intensity    selection rules
#                         cm**(-1)        km/mol         IR     RAMAN
     1                        0.00         0.00000         -       -
     2                        0.00         0.00000         -       -
     3                        0.00         0.00000         -       -
     4                        0.00         0.00000         -       -
     5                        0.00         0.00000         -       -
     6                        0.00         0.00000         -       -
     7         a             10.60         0.12707        YES     YES
     8         a             18.77         0.06926        YES     YES
     9         a             22.73         0.01302        YES     YES
    10         a             27.65         1.50588        YES     YES
    11         a             36.53         0.09112        YES     YES
    12         a             39.83         3.37804        YES     YES
    13         a             48.74         0.34111        YES     YES
    14         a             53.54         0.73119        YES     YES
    15         a             64.55         0.21252        YES     YES
    16         a             69.69         0.59823        YES     YES
    17         a             83.07         0.31250        YES     YES
    18         a             90.85         1.55997        YES     YES
    19         a             96.22         0.11657        YES     YES
    20         a            103.38         0.31201        YES     YES
    21         a            104.41         1.47409        YES     YES
    22         a            106.84         0.66816        YES     YES
    23         a            121.59         2.22071        YES     YES
    24         a            124.39         1.86083        YES     YES
    25         a            145.46         0.52090        YES     YES
    26         a            148.57         0.60446        YES     YES
    27         a            156.10         0.69356        YES     YES
    28         a            163.80         2.59192        YES     YES
    29         a            165.79         0.88762        YES     YES
    30         a            177.22         0.76187        YES     YES
    31         a            216.32         1.12605        YES     YES
    32         a            226.70         0.37322        YES     YES
    33         a            250.73         0.56391        YES     YES
    34         a            267.86         3.80032        YES     YES
    35         a            270.94         1.92585        YES     YES
    36         a            292.80         0.91674        YES     YES
    37         a            304.24         3.90073        YES     YES
    38         a            313.54         0.96272        YES     YES
    39         a            345.65         8.33874        YES     YES
    40         a            364.81         1.91105        YES     YES
    41         a            396.25         0.41374        YES     YES
    42         a            400.82         0.02306        YES     YES
    43         a            403.36         0.00668        YES     YES
    44         a            423.45         2.55893        YES     YES
    45         a            439.30         4.76361        YES     YES
    46         a            447.38         0.83549        YES     YES
    47         a            465.34         1.13082        YES     YES
    48         a            476.14         1.83319        YES     YES
    49         a            480.49         0.25021        YES     YES
    50         a            489.34         2.18610        YES     YES
```

Complex TSIVPyr-VIPyr

SCF Energy (au) (RI)BP86/SV(P) -2736.0577348460
SCF Energy (au) PBE0/def2-TZVPP -2735.569083961
SCF Energy (au) PBE0/def2-TZVPP -2735.5870414927 (Et2O Correction)
Zero Point Energy (au) 0.4072919
Chemical potential (kJ mol-1) 896.60
Dispersion correction (au) PBE0/def2-TZVPP -0.09058672

xyz coordinates

57

C	1.16508	-0.13221	-1.28688
C	-0.81539	0.62006	-1.72791
Mn	0.54358	1.75039	-0.59536
C	1.59126	2.63497	0.60807
C	-0.46492	3.17686	-1.06709
O	-1.07832	4.10870	-1.40407
O	2.26587	3.23550	1.34419
C	-1.20806	0.27921	1.73110
C	-1.23326	2.60509	1.74188
C	-1.95454	2.67399	2.93533
C	-2.30728	1.47348	3.56848
C	-1.92933	0.27827	2.95607
C	-0.98167	-1.05591	1.11908
C	-1.38707	-1.35596	-0.18699
C	-1.63669	-2.77435	-0.54524
O	-1.25308	-3.74560	0.40339
C	-0.73444	-3.43626	1.61891
C	-0.62452	-2.13117	2.01293
H	-0.93325	3.53733	1.24375
H	-2.22172	3.65704	3.35285
H	-2.87849	1.46701	4.51116
H	-2.22390	-0.68724	3.39293
O	-2.13485	-3.18297	-1.57381
C	-0.33990	-4.64544	2.40770
H	-0.21417	-1.90653	3.00822
N	-0.84711	1.45003	1.13851
C	-1.14282	1.10651	-3.10185
C	-0.18529	1.21729	-4.13946
C	-0.55396	1.64631	-5.42369
C	-1.88549	1.99761	-5.70420
C	-2.84755	1.90453	-4.68497
C	-2.48296	1.45752	-3.40550
H	0.86632	0.95170	-3.95437
H	0.21278	1.70752	-6.21391
H	-2.17043	2.34438	-6.71121
H	-3.89605	2.18259	-4.88496
H	-3.24485	1.39328	-2.61127
C	-1.57673	-0.46005	-1.30873
H	-2.27260	-0.87850	-2.06115
C	2.47930	-0.60289	0.92935
C	2.53808	-0.10296	2.25482
C	3.42279	-0.65241	3.19500
C	4.26241	-1.72244	2.84012
C	4.21049	-2.23829	1.53120
C	3.33399	-1.68826	0.58798
H	1.88282	0.73406	2.54379
H	3.45703	-0.23816	4.21671
H	4.95947	-2.15046	3.57889
H	4.86890	-3.07239	1.24010
H	3.30492	-2.08830	-0.43832
C	1.58430	-0.06250	-0.07220

```
H          1.24892       -0.61040       -2.26524
C          1.69415        2.27749       -1.84314
O          2.48126        2.66216       -2.61533
H          0.06072       -4.35733        3.40046
H          0.43757       -5.22812        1.86370
H         -1.21129       -5.32379        2.55017

$vibrational spectrum
#  mode     symmetry     wave number   IR intensity    selection rules
#                         cm**(-1)        km/mol         IR     RAMAN
     1         a           -126.09         0.00000       YES     YES
     2                        0.00         0.00000        -       -
     3                        0.00         0.00000        -       -
     4                        0.00         0.00000        -       -
     5                        0.00         0.00000        -       -
     6                        0.00         0.00000        -       -
     7                        0.00         0.00000        -       -
     8         a             20.75         1.33190       YES     YES
     9         a             25.14         0.90576       YES     YES
    10         a             33.77         0.90974       YES     YES
    11         a             37.71         0.28903       YES     YES
    12         a             41.27         0.36760       YES     YES
    13         a             47.81         0.84262       YES     YES
    14         a             59.72         0.60188       YES     YES
    15         a             63.72         0.90920       YES     YES
    16         a             66.99         0.42839       YES     YES
    17         a             84.27         0.10213       YES     YES
    18         a             92.77         0.29021       YES     YES
    19         a             98.63         0.87071       YES     YES
    20         a            103.90         0.20573       YES     YES
    21         a            111.97         1.82587       YES     YES
    22         a            118.06         0.07214       YES     YES
    23         a            126.40         0.91078       YES     YES
    24         a            129.13         0.57487       YES     YES
    25         a            143.86         0.15287       YES     YES
    26         a            149.93         0.21838       YES     YES
    27         a            154.27         0.72833       YES     YES
    28         a            173.02         0.20286       YES     YES
    29         a            182.15         0.45225       YES     YES
    30         a            187.29         0.01856       YES     YES
    31         a            208.71         1.29654       YES     YES
    32         a            223.29         0.45165       YES     YES
    33         a            249.01         1.36406       YES     YES
    34         a            253.88         1.26543       YES     YES
    35         a            277.95         1.29286       YES     YES
    36         a            296.35         2.01391       YES     YES
    37         a            317.50         0.21890       YES     YES
    38         a            352.24         1.60591       YES     YES
    39         a            362.57         3.08701       YES     YES
    40         a            394.51        11.26856       YES     YES
    41         a            403.22         0.55837       YES     YES
    42         a            405.07         0.23267       YES     YES
    43         a            416.75        11.31451       YES     YES
    44         a            427.86         0.19529       YES     YES
    45         a            441.52         7.04775       YES     YES
    46         a            447.02         0.49481       YES     YES
    47         a            467.98         2.43716       YES     YES
    48         a            470.64         0.73833       YES     YES
    49         a            471.59         0.89960       YES     YES
    50         a            485.49         4.18052       YES     YES
```

Complex VIPyr

SCF Energy (au) (RI)BP86/SV(P) -2736.1636935130
SCF Energy (au) PBE0/def2-TZVPP -2735.685847324
SCF Energy (au) PBE0/def2-TZVPP -2735.7030750593 (Et2O Correction)
Zero Point Energy (au) 0.4122093
Chemical potential (kJ mol-1) 915.98
Dispersion correction (au) PBE0/def2-TZVPP -0.09115484

xyz coordinates

57

C	0.96337	-0.71215	-0.69271
C	0.03209	-0.59406	-1.78901
Mn	0.86850	1.38787	-0.90404
C	1.66785	2.61019	0.14678
C	0.49859	2.39569	-2.35303
O	0.24917	3.01927	-3.30640
O	2.20764	3.40441	0.80874
C	-1.37439	1.25245	1.05837
C	-1.39698	3.27246	-0.11268
C	-2.37541	3.80905	0.72800
C	-2.83317	3.03758	1.80947
C	-2.32955	1.74125	1.96798
C	-0.90539	-0.20915	1.00082
C	-1.79848	-0.72741	-0.12741
C	-3.17503	-1.16771	0.16126
O	-3.42972	-1.54962	1.48556
C	-2.41778	-1.60384	2.42636
C	-1.22269	-0.99303	2.25613
H	-0.99049	3.86186	-0.94862
H	-2.75649	4.82422	0.53833
H	-3.58008	3.43857	2.51387
H	-2.66273	1.08869	2.78870
O	-4.08309	-1.27585	-0.63733
C	-2.83965	-2.39658	3.62772
H	-0.47248	-1.06481	3.05611
N	-0.88452	2.02747	0.05318
C	0.42744	-0.90974	-3.19774
C	1.58102	-1.66792	-3.50880
C	1.91715	-1.96662	-4.83889
C	1.10665	-1.52016	-5.89544
C	-0.04570	-0.76927	-5.60478
C	-0.37837	-0.46854	-4.27610
H	2.22659	-2.05389	-2.70397
H	2.82084	-2.56292	-5.04809
H	1.37158	-1.75519	-6.93936
H	-0.69039	-0.40514	-6.42187
H	-1.27208	0.14382	-4.07175
C	-1.36773	-0.72430	-1.42856
H	-2.10460	-0.97248	-2.21023
C	1.61197	-0.39998	1.72343
C	1.64991	0.53918	2.78295
C	2.57161	0.41383	3.83267
C	3.48268	-0.65717	3.85328
C	3.45299	-1.60433	2.81709
C	2.52419	-1.48057	1.77071
H	0.95556	1.39590	2.77575
H	2.58564	1.16718	4.63788
H	4.21061	-0.75339	4.67567
H	4.15227	-2.45707	2.82518
H	2.49370	-2.25443	0.98633
C	0.61884	-0.26898	0.60883

```
H          1.96052       -1.14319       -0.86565
C          2.49512        1.10532       -1.60234
O          3.56882        0.97627       -2.03956
H         -3.76140       -1.96415        4.07773
H         -2.03899       -2.41676        4.39480
H         -3.08554       -3.44212        3.33471

$vibrational spectrum
#  mode     symmetry     wave number   IR intensity    selection rules
#                         cm**(-1)        km/mol         IR     RAMAN
     1                        0.00         0.00000        -       -
     2                        0.00         0.00000        -       -
     3                        0.00         0.00000        -       -
     4                        0.00         0.00000        -       -
     5                        0.00         0.00000        -       -
     6                        0.00         0.00000        -       -
     7         a             28.53         0.68977       YES     YES
     8         a             35.84         0.54891       YES     YES
     9         a             43.11         0.61740       YES     YES
    10         a             49.02         0.42889       YES     YES
    11         a             52.72         0.16039       YES     YES
    12         a             58.49         0.48260       YES     YES
    13         a             62.99         0.54657       YES     YES
    14         a             67.85         2.08399       YES     YES
    15         a             74.34         0.36529       YES     YES
    16         a             77.08         0.30519       YES     YES
    17         a             86.55         0.37155       YES     YES
    18         a             96.76         0.83481       YES     YES
    19         a            102.56         0.26502       YES     YES
    20         a            110.04         0.11188       YES     YES
    21         a            112.00         0.25477       YES     YES
    22         a            133.80         0.99958       YES     YES
    23         a            148.99         0.53513       YES     YES
    24         a            169.10         4.81315       YES     YES
    25         a            175.84         0.08170       YES     YES
    26         a            181.83         1.24683       YES     YES
    27         a            186.88         0.60170       YES     YES
    28         a            206.69         0.64735       YES     YES
    29         a            210.80         1.05197       YES     YES
    30         a            236.05         0.35115       YES     YES
    31         a            241.37         0.10681       YES     YES
    32         a            254.51         1.80755       YES     YES
    33         a            265.78         0.83141       YES     YES
    34         a            291.99         0.78548       YES     YES
    35         a            309.89         0.51414       YES     YES
    36         a            313.25         2.27064       YES     YES
    37         a            372.41         0.87082       YES     YES
    38         a            378.52         1.31233       YES     YES
    39         a            403.80         0.09440       YES     YES
    40         a            405.87         0.18208       YES     YES
    41         a            418.56         2.85922       YES     YES
    42         a            427.52         3.57559       YES     YES
    43         a            436.26         0.61482       YES     YES
    44         a            451.03        12.67021       YES     YES
    45         a            463.21         5.31219       YES     YES
    46         a            474.22         4.31501       YES     YES
    47         a            476.10         2.99469       YES     YES
    48         a            488.34         4.24030       YES     YES
    49         a            496.47         0.59449       YES     YES
    50         a            500.58         3.79121       YES     YES
```

Complex VIIaPyr

SCF Energy (au) (RI)BP86/SV(P) -2736.1404415700
SCF Energy (au) PBE0/def2-TZVPP -2735.667640088
SCF Energy (au) PBE0/def2-TZVPP -2735.6875460277 (Et2O Correction)
Zero Point Energy (au) 0.4117276
Chemical potential (kJ mol-1) 908.69
Dispersion correction (au) PBE0/def2-TZVPP -0.08342747

xyz coordinates

57

C	0.84852	-0.79654	-0.08949
C	-0.00130	-0.79931	-1.27136
Mn	1.36425	0.48213	-4.71697
C	0.58814	1.17064	-6.17984
C	2.92594	1.17665	-5.25507
O	3.94849	1.61079	-5.60695
O	0.08007	1.60438	-7.13425
C	-1.21171	1.33119	1.51246
C	-0.71398	3.34105	0.48483
C	-1.41338	4.08606	1.45259
C	-2.04064	3.38450	2.49243
C	-1.94297	1.98300	2.52585
C	-1.08351	-0.23724	1.43422
C	-1.94173	-0.65143	0.23770
C	-3.39979	-0.84072	0.40854
O	-3.84047	-1.09213	1.70806
C	-2.93954	-1.30236	2.74126
C	-1.64550	-0.92193	2.66542
H	-0.21030	3.84487	-0.36138
H	-1.46564	5.18490	1.38580
H	-2.60513	3.92043	3.27419
H	-2.42175	1.39893	3.32632
O	-4.23276	-0.83490	-0.47668
C	-3.60343	-1.97651	3.90506
H	-0.98718	-1.11797	3.52412
N	-0.61628	2.00549	0.51503
C	0.51413	-0.85796	-2.57001
C	1.93949	-0.77870	-2.90851
C	2.44686	-1.29136	-4.12726
C	1.56913	-1.59944	-5.21279
C	0.18240	-1.30573	-5.02504
C	-0.29230	-0.79415	-3.79328
H	2.66718	-0.47196	-2.14228
H	3.53668	-1.37914	-4.26625
H	1.94853	-2.00068	-6.16317
H	-0.51373	-1.40335	-5.87374
H	-1.35108	-0.50073	-3.73856
C	-1.42312	-0.80572	-1.01816
H	-2.14052	-1.01373	-1.82791
C	1.34412	-0.67884	2.32723
C	1.31179	0.24235	3.40528
C	2.24619	0.17060	4.44995
C	3.23631	-0.82683	4.45408
C	3.27819	-1.75624	3.40058
C	2.34340	-1.68539	2.35715
H	0.55775	1.04518	3.41503
H	2.20271	0.90892	5.26834
H	3.96692	-0.88383	5.27800
H	4.03811	-2.55583	3.39784
H	2.36294	-2.44422	1.55745
C	0.39471	-0.60168	1.18666

H	1.92755	-0.97563	-0.22325
C	0.94750	1.91196	-3.68476
O	0.68541	2.83891	-3.03518
H	-4.46414	-1.37102	4.26923
H	-2.88884	-2.12316	4.74018
H	-4.01269	-2.96657	3.60128

$vibrational spectrum

# mode	symmetry	wave number cm**(-1)	IR intensity km/mol	selection rules IR	RAMAN
1		0.00	0.00000	-	-
2		0.00	0.00000	-	-
3		0.00	0.00000	-	-
4		0.00	0.00000	-	-
5		0.00	0.00000	-	-
6		0.00	0.00000	-	-
7	a	22.19	0.45218	YES	YES
8	a	23.40	0.72428	YES	YES
9	a	32.61	0.40062	YES	YES
10	a	35.57	0.73030	YES	YES
11	a	43.31	1.03754	YES	YES
12	a	49.76	0.59836	YES	YES
13	a	51.50	0.37238	YES	YES
14	a	59.31	0.44382	YES	YES
15	a	67.82	1.76076	YES	YES
16	a	73.45	0.76073	YES	YES
17	a	88.04	0.28993	YES	YES
18	a	90.24	0.73803	YES	YES
19	a	94.19	0.70474	YES	YES
20	a	109.19	0.76453	YES	YES
21	a	125.04	1.21787	YES	YES
22	a	128.42	0.49591	YES	YES
23	a	134.76	0.79013	YES	YES
24	a	158.38	1.29042	YES	YES
25	a	166.42	4.79271	YES	YES
26	a	185.34	0.46261	YES	YES
27	a	189.93	0.57862	YES	YES
28	a	202.24	12.17296	YES	YES
29	a	227.34	1.14306	YES	YES
30	a	245.87	1.59119	YES	YES
31	a	253.04	1.66329	YES	YES
32	a	274.41	3.41708	YES	YES
33	a	283.78	1.10579	YES	YES
34	a	288.61	15.80083	YES	YES
35	a	300.32	0.41936	YES	YES
36	a	308.36	5.61591	YES	YES
37	a	358.74	10.41136	YES	YES
38	a	368.45	13.55502	YES	YES
39	a	398.62	1.88338	YES	YES
40	a	405.67	1.02586	YES	YES
41	a	412.44	5.79259	YES	YES
42	a	416.22	0.47712	YES	YES
43	a	429.64	4.95936	YES	YES
44	a	446.25	1.06380	YES	YES
45	a	458.16	2.67218	YES	YES
46	a	464.77	10.49925	YES	YES
47	a	476.97	3.05662	YES	YES
48	a	484.54	0.04239	YES	YES
49	a	490.84	11.15675	YES	YES
50	a	498.63	0.88253	YES	YES

Complex VIIbPyr

SCF Energy (au) (RI)BP86/SV(P) -2736.1394375500
SCF Energy (au) PBE0/def2-TZVPP -2735.666589029
SCF Energy (au) PBE0/def2-TZVPP -2735.6873418509 (Et2O Correction)
Zero Point Energy (au) 0.4115630
Chemical potential (kJ mol-1) 906.29
Dispersion correction (au) PBE0/def2-TZVPP -0.08284095

xyz coordinates

57

C	-0.47793	0.23153	-0.73972
C	0.96388	0.12013	-0.90366
Mn	2.64560	1.24371	-4.26860
C	2.01418	1.92814	-5.79984
C	4.27660	1.85468	-4.69758
O	5.34242	2.23512	-4.97351
O	1.59581	2.35743	-6.79903
C	-0.43526	-1.63141	2.09175
C	-0.25493	-3.78262	1.26918
C	-0.46361	-4.35967	2.53523
C	-0.66379	-3.49590	3.62203
C	-0.65032	-2.10878	3.40102
C	-0.33983	-0.09540	1.75995
C	1.15098	0.17447	1.55167
C	2.02353	0.40013	2.72576
O	1.38780	0.84210	3.88658
C	0.04430	1.18775	3.87937
C	-0.80518	0.77296	2.91427
H	-0.09164	-4.42418	0.38194
H	-0.46603	-5.45464	2.66098
H	-0.82967	-3.89495	4.63690
H	-0.81096	-1.40138	4.22836
O	3.23222	0.27756	2.75723
C	-0.30019	2.03910	5.06500
H	-1.85990	1.07976	2.96968
N	-0.23827	-2.46087	1.05256
C	1.57451	0.01456	-2.15931
C	3.02206	0.00457	-2.38688
C	3.59426	-0.56258	-3.55047
C	2.79006	-0.85623	-4.69557
C	1.40966	-0.49291	-4.62313
C	0.86986	0.07624	-3.44380
H	3.70680	0.27596	-1.56972
H	4.68537	-0.70893	-3.59895
H	3.22111	-1.30293	-5.60261
H	0.77473	-0.58393	-5.51893
H	-0.17784	0.40914	-3.47883
C	1.73531	0.17450	0.31535
H	2.83157	0.27901	0.28977
C	-2.60092	0.35643	0.51658
C	-3.42332	-0.42298	1.37027
C	-4.81891	-0.27315	1.37129
C	-5.43566	0.66478	0.52550
C	-4.63747	1.45689	-0.31818
C	-3.24266	1.30798	-0.31790
H	-2.96569	-1.17748	2.02900
H	-5.43146	-0.90293	2.03812
H	-6.53178	0.78417	0.53035
H	-5.10465	2.21137	-0.97317
H	-2.62654	1.96455	-0.95388
C	-1.12522	0.19010	0.46531

H	-1.09915	0.33755	-1.64283
C	2.21583	2.70868	-3.29428
O	1.93526	3.64732	-2.66948
H	-0.04537	1.51214	6.01239
H	-1.38023	2.29062	5.07333
H	0.29063	2.98270	5.05313

$vibrational spectrum

# mode	symmetry	wave number cm**(-1)	IR intensity km/mol	selection rules IR	selection rules RAMAN
1		0.00	0.00000	-	-
2		0.00	0.00000	-	-
3		0.00	0.00000	-	-
4		0.00	0.00000	-	-
5		0.00	0.00000	-	-
6		0.00	0.00000	-	-
7	a	17.24	0.29641	YES	YES
8	a	19.72	0.56863	YES	YES
9	a	30.85	0.39960	YES	YES
10	a	34.08	0.43154	YES	YES
11	a	36.92	1.93324	YES	YES
12	a	48.03	0.72010	YES	YES
13	a	51.54	0.84607	YES	YES
14	a	55.97	0.11676	YES	YES
15	a	69.56	0.86234	YES	YES
16	a	74.59	0.18048	YES	YES
17	a	87.46	0.35852	YES	YES
18	a	89.89	0.74218	YES	YES
19	a	96.78	1.11758	YES	YES
20	a	107.63	0.85561	YES	YES
21	a	124.92	0.08034	YES	YES
22	a	129.23	2.54816	YES	YES
23	a	132.68	2.10952	YES	YES
24	a	159.38	7.05120	YES	YES
25	a	163.08	0.75508	YES	YES
26	a	178.92	1.15557	YES	YES
27	a	184.04	1.14819	YES	YES
28	a	213.17	3.34324	YES	YES
29	a	226.81	0.53736	YES	YES
30	a	248.01	1.24711	YES	YES
31	a	256.47	1.90596	YES	YES
32	a	265.96	4.09162	YES	YES
33	a	278.59	4.21844	YES	YES
34	a	288.76	16.86762	YES	YES
35	a	297.65	0.72810	YES	YES
36	a	306.99	7.88163	YES	YES
37	a	357.41	13.15441	YES	YES
38	a	370.04	8.68079	YES	YES
39	a	397.16	3.48921	YES	YES
40	a	405.96	0.02860	YES	YES
41	a	410.45	0.20325	YES	YES
42	a	415.15	0.74217	YES	YES
43	a	426.10	2.74909	YES	YES
44	a	445.62	1.08232	YES	YES
45	a	455.96	6.90865	YES	YES
46	a	460.42	14.05439	YES	YES
47	a	481.58	1.51755	YES	YES
48	a	484.31	0.36260	YES	YES
49	a	492.18	10.83211	YES	YES
50	a	498.46	0.67623	YES	YES

Complex 10Pyr

SCF Energy (au) (RI)BP86/SV(P) -2736.1734779040
SCF Energy (au) PBE0/def2-TZVPP -2735.701230530
SCF Energy (au) PBE0/def2-TZVPP -2735.7182033021 (Et2O Correction)
Zero Point Energy (au) 0.4125312
Chemical potential (kJ mol-1) 914.40
Dispersion correction (au) PBE0/def2-TZVPP -0.08945789

xyz coordinates

57

C	-2.81574	-1.36747	1.31387
C	-1.17582	-1.43824	3.20210
C	-0.64360	-2.85048	1.05749
C	1.03291	-0.45144	1.32830
C	0.19482	0.54317	1.90867
H	0.39699	0.86490	2.94203
C	-0.93430	1.03433	1.18431
C	-1.18179	0.39169	-0.08736
H	-2.14363	0.58885	-0.58903
C	-0.28774	-0.53811	-0.67624
C	1.18310	-0.51066	-0.19845
C	1.95138	-1.77300	-0.53802
H	1.92649	-2.15932	-1.56565
C	2.71916	-2.42886	0.35827
C	3.53697	-3.65404	0.08201
H	4.60830	-3.46498	0.31823
H	3.45084	-3.95950	-0.98028
H	3.20302	-4.49737	0.72746
C	2.05851	-1.05097	2.22212
C	1.99811	0.72251	-0.67402
C	1.54163	1.65701	-1.62370
H	0.55091	1.54853	-2.09177
C	2.37415	2.73792	-1.96331
H	2.04385	3.48068	-2.70883
C	3.62203	2.85874	-1.33571
H	4.30459	3.69213	-1.56731
C	3.97849	1.88283	-0.38545
C	-1.82459	2.10842	1.70170
C	-2.07157	2.26496	3.08616
H	-1.63648	1.55257	3.80564
C	-2.89549	3.29739	3.55541
H	-3.08245	3.39390	4.63755
C	-3.49230	4.19640	2.65283
H	-4.14303	5.00538	3.02354
C	-3.25431	4.05408	1.27630
H	-3.71087	4.75629	0.55928
C	-2.42715	3.02122	0.80576
H	-2.22709	2.93826	-0.27511
C	-0.70188	-1.19297	-1.95657
C	-1.98043	-1.78514	-2.09758
H	-2.65033	-1.84417	-1.22621
C	-2.40484	-2.32296	-3.32147
H	-3.40379	-2.78388	-3.39336
C	-1.55816	-2.29111	-4.44278
H	-1.88727	-2.72134	-5.40288
C	-0.28531	-1.71013	-4.32383
H	0.39019	-1.67170	-5.19445
C	0.13847	-1.16982	-3.09870
H	1.13198	-0.69858	-3.04446
Mn	-1.04000	-1.13040	1.43595
N	3.19443	0.84546	-0.06662

```
O         -3.97108       -1.51830        1.25056
O         -1.28590       -1.62771        4.34496
O         -0.42558       -3.97041        0.83053
O          2.84700       -2.04499        1.68217
O          2.21778       -0.75350        3.38892
H          4.94643        1.94395        0.14699

$vibrational spectrum
#  mode     symmetry     wave number   IR intensity    selection rules
#                         cm**(-1)        km/mol         IR     RAMAN
     1                       0.00         0.00000        -       -
     2                       0.00         0.00000        -       -
     3                       0.00         0.00000        -       -
     4                       0.00         0.00000        -       -
     5                       0.00         0.00000        -       -
     6                       0.00         0.00000        -       -
     7         a            30.37         0.30743       YES     YES
     8         a            38.60         0.26953       YES     YES
     9         a            41.61         0.14421       YES     YES
    10         a            43.29         0.34238       YES     YES
    11         a            48.08         0.23305       YES     YES
    12         a            50.87         0.08415       YES     YES
    13         a            59.85         0.01919       YES     YES
    14         a            62.59         1.26855       YES     YES
    15         a            69.95         0.27641       YES     YES
    16         a            72.58         1.23333       YES     YES
    17         a            81.21         0.53748       YES     YES
    18         a            88.54         0.11153       YES     YES
    19         a            92.71         0.12698       YES     YES
    20         a            98.85         0.35985       YES     YES
    21         a           110.77         0.18075       YES     YES
    22         a           123.42         0.05897       YES     YES
    23         a           145.36         0.37683       YES     YES
    24         a           150.78         1.13323       YES     YES
    25         a           167.88         1.40564       YES     YES
    26         a           179.77         2.11708       YES     YES
    27         a           188.22         1.21751       YES     YES
    28         a           191.24         1.36356       YES     YES
    29         a           204.47         0.65811       YES     YES
    30         a           225.72         3.59492       YES     YES
    31         a           238.47         0.51891       YES     YES
    32         a           254.90         0.84707       YES     YES
    33         a           271.23         3.18787       YES     YES
    34         a           292.02         1.33207       YES     YES
    35         a           309.72         0.64846       YES     YES
    36         a           334.75         2.52795       YES     YES
    37         a           370.85         1.21983       YES     YES
    38         a           383.70         3.99708       YES     YES
    39         a           399.43         1.78093       YES     YES
    40         a           402.21         0.40040       YES     YES
    41         a           409.81         0.65514       YES     YES
    42         a           420.24         2.54045       YES     YES
    43         a           432.55         4.77996       YES     YES
    44         a           442.22         2.43238       YES     YES
    45         a           450.49         6.72644       YES     YES
    46         a           471.97         3.35908       YES     YES
    47         a           480.90         0.76913       YES     YES
    48         a           484.04         0.37070       YES     YES
    49         a           498.96         1.55128       YES     YES
    50         a           510.03        12.89882       YES     YES
```

Complex IIPh

SCF Energy (au) (RI)BP86/SV(P) -2277.5161934120
SCF Energy (au) PBE0/def2-TZVPP -2277.070662383
SCF Energy (au) PBE0/def2-TZVPP -2277.0814583946 (Et2O Correction)
Zero Point Energy (au) 0.2897634
Chemical potential (kJ mol-1) 615.48
Dispersion correction (au) PBE0/def2-TZVPP -0.06121589

xyz coordinates

41

Mn	1.30245	-0.02169	0.74661
C	1.62450	0.20050	2.50505
C	2.95518	-0.65910	0.47892
C	1.80850	1.68376	0.37645
O	2.16975	2.77915	0.19703
O	4.02418	-1.09454	0.30725
O	1.83742	0.33700	3.64134
C	-1.50058	-0.52608	1.56873
C	-1.27856	1.65238	0.71772
C	-2.63581	1.93251	0.90926
C	-3.45321	0.92907	1.45802
C	-2.88049	-0.30226	1.78637
C	-0.79093	-1.77234	1.87664
C	0.60334	-1.78573	1.57636
C	1.32783	-2.95859	1.87556
C	0.69738	-4.08534	2.43189
C	-0.68244	-4.06455	2.71326
C	-1.42254	-2.90964	2.43720
H	-0.60440	2.41196	0.29343
H	-3.03434	2.92083	0.63358
H	-4.52759	1.10675	1.62928
H	-3.49983	-1.10273	2.21649
H	2.41075	-3.00739	1.67158
H	1.29065	-4.98956	2.65166
H	-1.17666	-4.94690	3.15149
H	-2.50135	-2.89703	2.66437
N	-0.71744	0.46477	1.03412
C	0.73330	1.04190	-2.46476
C	1.87490	1.75765	-2.90758
C	1.75933	2.72560	-3.91464
C	0.50805	3.00721	-4.49220
C	-0.63158	2.30703	-4.05887
C	-0.52486	1.33316	-3.05520
H	2.85681	1.54056	-2.45926
H	2.65784	3.26873	-4.25112
H	0.42142	3.77294	-5.28063
H	-1.61579	2.51924	-4.50883
H	-1.41520	0.77891	-2.71836
C	0.82108	0.00599	-1.46154
C	0.69860	-1.14191	-0.94251
H	0.51034	-2.21220	-1.03415

```
$vibrational spectrum
#  mode     symmetry     wave number   IR intensity    selection rules
#                         cm**(-1)        km/mol         IR     RAMAN
     1                       0.00         0.00000         -       -
     2                       0.00         0.00000         -       -
     3                       0.00         0.00000         -       -
     4                       0.00         0.00000         -       -
     5                       0.00         0.00000         -       -
     6                       0.00         0.00000         -       -
     7         a            16.92         0.18196        YES     YES
     8         a            29.12         0.18730        YES     YES
     9         a            39.44         0.04922        YES     YES
    10         a            57.29         0.28357        YES     YES
    11         a            69.45         0.06837        YES     YES
    12         a            74.51         0.10817        YES     YES
    13         a            85.75         1.34343        YES     YES
    14         a            90.69         0.27811        YES     YES
    15         a            99.33         0.44510        YES     YES
    16         a           109.87         0.12107        YES     YES
    17         a           113.33         0.10699        YES     YES
    18         a           118.28         0.53725        YES     YES
    19         a           129.86         0.33981        YES     YES
    20         a           162.72         1.55487        YES     YES
    21         a           164.97         5.40992        YES     YES
    22         a           189.65         1.22846        YES     YES
    23         a           222.86         0.69322        YES     YES
    24         a           242.13         2.15168        YES     YES
    25         a           262.03         0.56601        YES     YES
    26         a           283.38         1.93927        YES     YES
    27         a           320.56        23.66758        YES     YES
    28         a           358.49         1.90009        YES     YES
    29         a           401.23         0.01985        YES     YES
    30         a           410.51         1.59782        YES     YES
    31         a           420.06        11.11831        YES     YES
    32         a           437.09         2.13531        YES     YES
    33         a           454.94        15.46878        YES     YES
    34         a           456.15         2.06895        YES     YES
    35         a           471.44         3.94462        YES     YES
    36         a           480.07         5.73117        YES     YES
    37         a           486.53         2.17161        YES     YES
    38         a           492.67         5.76252        YES     YES
    39         a           501.93         9.24113        YES     YES
    40         a           508.11        12.94047        YES     YES
    41         a           524.80        34.54409        YES     YES
    42         a           539.50        10.09634        YES     YES
    43         a           550.69         5.86095        YES     YES
    44         a           556.63         1.71211        YES     YES
    45         a           609.36        20.54643        YES     YES
    46         a           614.44         8.33273        YES     YES
    47         a           623.38         6.75777        YES     YES
    48         a           625.77        13.21340        YES     YES
    49         a           643.19         9.46298        YES     YES
    50         a           654.08        72.32014        YES     YES
```

TSIIPh-IIIPh

SCF Energy (au) (RI)BP86/SV(P) -2277.5084109160
SCF Energy (au) PBE0/def2-TZVPP -2277.059171549
SCF Energy (au) PBE0/def2-TZVPP -2277.0697855917 (Et2O Correction)
Zero Point Energy (au) 0.2894069
Chemical potential (kJ mol-1) 617.36
Dispersion correction (au) PBE0/def2-TZVPP -0.06065131

xyz coordinates

41

Mn	1.54771	0.37033	0.77601
C	1.90922	0.62895	2.52872
C	3.24140	-0.11505	0.46337
C	1.95376	2.07699	0.34740
O	2.25077	3.17945	0.09864
O	4.34482	-0.44157	0.26518
O	2.13108	0.78743	3.66028
C	-1.25257	-0.25182	1.59649
C	-1.09401	1.97436	0.86730
C	-2.45843	2.20000	1.06765
C	-3.25128	1.13811	1.54032
C	-2.64471	-0.09211	1.79905
C	-0.50252	-1.47892	1.85703
C	0.85104	-1.50084	1.39103
C	1.63475	-2.64377	1.70773
C	1.10095	-3.71846	2.42376
C	-0.24841	-3.70067	2.84500
C	-1.04014	-2.58582	2.56068
H	-0.44310	2.77510	0.48495
H	-2.88633	3.19025	0.84886
H	-4.33388	1.27053	1.70011
H	-3.24405	-0.94267	2.15554
H	2.68380	-2.69141	1.36963
H	1.74052	-4.58496	2.66285
H	-0.66924	-4.55134	3.40486
H	-2.08268	-2.56067	2.91786
N	-0.49743	0.78851	1.12252
C	0.34801	0.66034	-2.37847
C	0.95629	1.84675	-2.86602
C	0.54552	2.41442	-4.08065
C	-0.49223	1.83066	-4.82884
C	-1.11497	0.66209	-4.35225
C	-0.70219	0.08143	-3.14640
H	1.76593	2.31854	-2.29063
H	1.04080	3.32980	-4.44524
H	-0.81766	2.28588	-5.77877
H	-1.93161	0.19565	-4.92854
H	-1.19149	-0.83284	-2.77347
C	0.75854	0.00615	-1.15816
C	0.94124	-1.13956	-0.58432
H	1.15278	-2.17924	-0.85105

$vibrational spectrum

# mode	symmetry	wave number cm**(-1)	IR intensity km/mol	selection rules IR	RAMAN
1	a	-229.92	0.00000	YES	YES
2		0.00	0.00000	-	-
3		0.00	0.00000	-	-
4		0.00	0.00000	-	-
5		0.00	0.00000	-	-
6		0.00	0.00000	-	-
7		0.00	0.00000	-	-
8	a	17.41	0.06220	YES	YES
9	a	25.16	0.19062	YES	YES
10	a	45.64	0.05440	YES	YES
11	a	58.62	0.10755	YES	YES
12	a	71.56	0.08061	YES	YES
13	a	82.91	0.01235	YES	YES
14	a	89.59	0.27702	YES	YES
15	a	92.91	1.10365	YES	YES
16	a	100.44	0.45908	YES	YES
17	a	110.09	0.06448	YES	YES
18	a	116.49	0.03755	YES	YES
19	a	132.59	0.36059	YES	YES
20	a	162.30	1.18698	YES	YES
21	a	181.99	0.55163	YES	YES
22	a	187.34	0.53494	YES	YES
23	a	217.21	0.99851	YES	YES
24	a	233.62	0.81122	YES	YES
25	a	259.66	0.64011	YES	YES
26	a	278.88	0.12099	YES	YES
27	a	343.64	8.47045	YES	YES
28	a	359.48	1.38284	YES	YES
29	a	385.75	2.26724	YES	YES
30	a	402.48	0.07176	YES	YES
31	a	415.90	3.19141	YES	YES
32	a	430.60	1.17126	YES	YES
33	a	447.49	0.40574	YES	YES
34	a	453.68	8.55989	YES	YES
35	a	463.23	5.74678	YES	YES
36	a	474.04	0.97570	YES	YES
37	a	488.66	0.39341	YES	YES
38	a	496.54	7.45279	YES	YES
39	a	503.72	5.28052	YES	YES
40	a	523.08	0.38776	YES	YES
41	a	544.07	14.03546	YES	YES
42	a	546.45	13.87922	YES	YES
43	a	553.17	0.60215	YES	YES
44	a	583.23	24.53609	YES	YES
45	a	610.37	10.84840	YES	YES
46	a	614.29	13.13794	YES	YES
47	a	620.34	1.05032	YES	YES
48	a	625.79	33.77338	YES	YES
49	a	639.14	0.32541	YES	YES
50	a	656.22	9.58836	YES	YES

Complex IIIPh

```
SCF Energy (au) (RI)BP86/SV(P)      -2277.5454614300
SCF Energy (au) PBE0/def2-TZVPP     -2277.104426951
SCF Energy (au) PBE0/def2-TZVPP     -2277.1164940469 (Et2O Correction)
Zero Point Energy (au)              0.2916092
Chemical potential (kJ mol-1)             622.45
Dispersion correction (au) PBE0/def2-TZVPP      -0.06485843
```

xyz coordinates

```
41

Mn    1.2036008    0.6106628    0.8522765
C     1.6744594    0.8952513    2.5996280
C     2.8473914    0.0459172    0.3776595
C     1.5911029    2.2600181    0.4058252
O     1.8151640    3.3749548    0.1224248
O     3.9082732   -0.3104898    0.0510345
O     2.0053518    1.0917446    3.7007502
C    -1.3227856   -0.2379977    1.6400043
C    -1.5435172    2.0145716    1.0272904
C    -2.9079081    2.0118246    1.3452868
C    -3.4880860    0.8331982    1.8413069
C    -2.6826598   -0.3075575    1.9890141
C    -0.3858537   -1.3933135    1.7625586
C     0.2684015   -1.9505773    0.6109429
C     1.0046730   -3.1537928    0.7947670
C     1.1266907   -3.7632905    2.0454927
C     0.5022348   -3.1927154    3.1746089
C    -0.2553715   -2.0286898    3.0253299
H    -1.0385621    2.9154598    0.6440124
H    -3.5010334    2.9288071    1.2049194
H    -4.5580140    0.7990083    2.1030172
H    -3.0974468   -1.2587283    2.3570313
H     1.4894566   -3.6036832   -0.0875235
H     1.7149517   -4.6900600    2.1489986
H     0.6023407   -3.6634756    4.1659099
H    -0.7646556   -1.5866450    3.8977602
N    -0.7721961    0.9179128    1.1840420
C     0.4618969    0.5684594   -2.2842737
C     1.4956238    1.4053105   -2.7767161
C     1.4172409    1.9808854   -4.0534696
C     0.2931146    1.7554251   -4.8682955
C    -0.7507065    0.9415658   -4.3932670
C    -0.6663964    0.3544298   -3.1217575
H     2.3830739    1.5885554   -2.1508602
H     2.2421910    2.6177027   -4.4145169
H     0.2279000    2.2188777   -5.8668102
H    -1.6422056    0.7651665   -5.0188331
H    -1.4944769   -0.2707101   -2.7486092
C     0.5386977   -0.0685731   -0.9603080
C     0.1585599   -1.3605615   -0.7492654
H    -0.1005158   -2.0548483   -1.5773864
```

$vibrational spectrum

# mode	symmetry	wave number	IR intensity	selection rules	
#		cm**(-1)	km/mol	IR	RAMAN
1		0.00	0.00000	-	-
2		0.00	0.00000	-	-
3		0.00	0.00000	-	-
4		0.00	0.00000	-	-
5		0.00	0.00000	-	-
6		0.00	0.00000	-	-
7	a	30.30	0.17649	YES	YES
8	a	36.56	0.28182	YES	YES
9	a	45.26	0.15260	YES	YES
10	a	53.87	0.01646	YES	YES
11	a	59.64	0.18618	YES	YES
12	a	71.73	0.36926	YES	YES
13	a	75.92	0.28896	YES	YES
14	a	85.62	0.08104	YES	YES
15	a	95.10	1.35102	YES	YES
16	a	100.25	0.88608	YES	YES
17	a	106.07	0.11247	YES	YES
18	a	109.83	0.57407	YES	YES
19	a	145.93	0.39566	YES	YES
20	a	164.65	1.78214	YES	YES
21	a	176.36	0.65196	YES	YES
22	a	201.48	0.39951	YES	YES
23	a	225.56	0.91647	YES	YES
24	a	241.41	1.04958	YES	YES
25	a	290.50	0.85078	YES	YES
26	a	305.47	0.62366	YES	YES
27	a	340.84	2.00198	YES	YES
28	a	359.20	1.66114	YES	YES
29	a	407.29	0.27575	YES	YES
30	a	411.66	2.09249	YES	YES
31	a	430.33	2.45302	YES	YES
32	a	448.21	0.79307	YES	YES
33	a	458.90	0.39480	YES	YES
34	a	476.58	0.31871	YES	YES
35	a	479.07	5.09467	YES	YES
36	a	491.65	1.11579	YES	YES
37	a	505.52	8.07195	YES	YES
38	a	505.80	6.51906	YES	YES
39	a	535.03	0.56159	YES	YES
40	a	540.90	5.74542	YES	YES
41	a	548.35	4.66086	YES	YES
42	a	555.33	0.93242	YES	YES
43	a	564.16	0.93933	YES	YES
44	a	613.21	0.87378	YES	YES
45	a	621.74	35.33440	YES	YES
46	a	622.91	1.39939	YES	YES
47	a	630.52	26.30650	YES	YES
48	a	633.59	22.26462	YES	YES
49	a	644.97	6.16755	YES	YES
50	a	696.00	24.05472	YES	YES

Complex TSIIIPh-5b-iso

SCF Energy (au) (RI)BP86/SV(P) -2277.5152698420
SCF Energy (au) PBE0/def2-TZVPP -2277.063594120
SCF Energy (au) PBE0/def2-TZVPP -2277.0739820475 (Et2O Correction)
Zero Point Energy (au) 0.2901506
Chemical potential (kJ mol-1) 622.05
Dispersion correction (au) PBE0/def2-TZVPP -0.06117935

xyz coordinates

41

C	2.13062	-0.13639	2.64741
C	-0.01201	1.08548	2.88500
C	0.55785	0.73144	0.05408
C	-0.06920	-1.57224	2.91070
C	1.17578	-1.70836	0.11918
C	2.28150	-2.63539	0.26517
C	0.90983	-4.47087	-0.57712
C	-0.17989	-3.62644	-0.72307
H	-1.14108	-4.03308	-1.07719
C	-0.10668	-2.25495	-0.33982
C	-1.20261	-1.35462	-0.36593
C	-2.40481	-1.51193	-1.11198
H	-2.49419	-2.37574	-1.78917
C	-3.44192	-0.60392	-0.98755
H	-4.37160	-0.72501	-1.56518
C	-3.27636	0.50507	-0.09916
H	-4.08999	1.22910	0.06327
C	-2.08589	0.68241	0.56272
H	-1.90627	1.54603	1.21910
Mn	0.54097	-0.23865	1.83350
N	-1.00989	-0.19505	0.42834
O	3.18378	-0.08842	3.15322
O	-0.35081	1.94662	3.60353
C	1.47758	-0.29365	-0.11618
O	-0.50274	-2.40464	3.59996
C	2.14755	-3.96836	-0.05890
H	2.51725	-0.03088	-0.37784
C	0.50683	2.05634	-0.54851
C	0.36846	4.60026	-1.79227
C	0.35517	4.49677	-0.38863
C	0.41156	3.23909	0.22815
C	0.51880	2.17685	-1.96587
C	0.44619	3.43510	-2.57804
H	0.31543	5.59038	-2.27458
H	0.30329	5.40566	0.23168
H	0.40172	3.16320	1.32618
H	0.57822	1.26347	-2.57953
H	0.45213	3.50999	-3.67791
H	0.81846	-5.53758	-0.83767
H	3.00135	-4.65341	0.07150
H	3.24563	-2.24366	0.62945

$vibrational spectrum

# mode	symmetry	wave number cm**(-1)	IR intensity km/mol	selection rules IR	RAMAN
1	a	-375.68	0.00000	YES	YES
2		0.00	0.00000	-	-
3		0.00	0.00000	-	-
4		0.00	0.00000	-	-
5		0.00	0.00000	-	-
6		0.00	0.00000	-	-
7		0.00	0.00000	-	-
8	a	24.73	0.07562	YES	YES
9	a	39.04	0.01211	YES	YES
10	a	49.50	0.08305	YES	YES
11	a	56.39	0.42235	YES	YES
12	a	64.74	0.04942	YES	YES
13	a	78.81	0.26565	YES	YES
14	a	87.66	0.01769	YES	YES
15	a	94.35	0.64672	YES	YES
16	a	100.92	1.80641	YES	YES
17	a	108.27	0.03835	YES	YES
18	a	123.20	1.45140	YES	YES
19	a	138.31	1.93404	YES	YES
20	a	152.49	1.42015	YES	YES
21	a	189.87	1.47018	YES	YES
22	a	212.44	2.83311	YES	YES
23	a	228.73	0.09515	YES	YES
24	a	254.23	0.39767	YES	YES
25	a	267.59	1.01387	YES	YES
26	a	292.14	2.24154	YES	YES
27	a	364.19	6.19346	YES	YES
28	a	374.72	0.17344	YES	YES
29	a	402.92	0.33476	YES	YES
30	a	413.71	5.16361	YES	YES
31	a	418.27	2.47638	YES	YES
32	a	436.42	2.34749	YES	YES
33	a	459.64	1.11727	YES	YES
34	a	467.63	3.99259	YES	YES
35	a	479.17	3.23969	YES	YES
36	a	485.07	1.46773	YES	YES
37	a	488.11	3.60391	YES	YES
38	a	517.21	6.91137	YES	YES
39	a	526.75	1.86171	YES	YES
40	a	537.20	20.33485	YES	YES
41	a	545.51	2.46971	YES	YES
42	a	548.80	10.02944	YES	YES
43	a	569.56	3.71402	YES	YES
44	a	601.09	18.49537	YES	YES
45	a	605.97	7.56066	YES	YES
46	a	617.33	17.18411	YES	YES
47	a	627.79	3.96100	YES	YES
48	a	632.90	11.10528	YES	YES
49	a	648.27	21.55982	YES	YES
50	a	677.61	8.97962	YES	YES

Complex IVPh

SCF Energy (au) (RI)BP86/SV(P) -2585.7120861790
SCF Energy (au) PBE0/def2-TZVPP -2585.222285558
SCF Energy (au) PBE0/def2-TZVPP -2585.2360610185 (Et2O Correction)
Zero Point Energy (au) 0.4004348
Chemical potential (kJ mol-1) 882.18
Dispersion correction (au) PBE0/def2-TZVPP -0.08677851

xyz coordinates

55

Mn	0.47599	1.17442	-0.62969
C	1.31857	2.43262	0.37135
C	-0.59765	2.46948	-1.28481
O	-1.23951	3.35150	-1.69136
O	1.83716	3.31149	0.93752
C	-1.38400	-0.39149	1.50387
C	-1.50879	1.93498	1.52669
C	-2.43384	1.95709	2.57210
C	-2.85712	0.73386	3.11131
C	-2.32460	-0.43568	2.56769
C	-0.90505	-1.71097	1.00664
C	-1.02091	-2.11982	-0.35406
C	-0.81530	-3.49461	-0.64744
C	-0.41746	-4.41738	0.32595
C	-0.23297	-3.98950	1.65335
C	-0.50288	-2.65595	1.98307
H	-1.16393	2.88629	1.09812
H	-2.80964	2.92278	2.94429
H	-3.59579	0.69078	3.92836
H	-2.65168	-1.42021	2.93345
H	-0.95104	-3.82630	-1.69098
H	-0.24341	-5.47041	0.04901
H	0.10250	-4.69446	2.43133
H	-0.38699	-2.32429	3.02810
N	-0.97846	0.80485	0.98649
C	-1.33083	0.48812	-3.15884
C	-0.47478	0.70276	-4.26652
C	-0.98134	1.13426	-5.50270
C	-2.35628	1.38007	-5.66380
C	-3.22095	1.17841	-4.57501
C	-2.71524	0.73351	-3.34260
H	0.60338	0.50647	-4.16634
H	-0.29104	1.27882	-6.35090
H	-2.75071	1.72868	-6.63277
H	-4.30218	1.36938	-4.68363
H	-3.39891	0.58099	-2.49084
C	-0.83300	-0.01931	-1.84314
C	-1.33993	-1.24055	-1.49092
H	-1.97822	-1.74383	-2.24954
C	2.80441	0.15007	1.72207
C	2.31373	0.72454	2.92147
C	3.14648	0.85405	4.04166
C	4.48380	0.42256	3.98758
C	4.98481	-0.14382	2.80155
C	4.15783	-0.27961	1.67821
H	1.26840	1.06661	2.96809
H	2.74777	1.30149	4.96712
H	5.13737	0.53211	4.86857
H	6.03344	-0.48058	2.74873
H	4.54887	-0.71921	0.74707
C	1.97402	-0.05769	0.55995

C	1.51200	-0.66021	-0.45103
H	1.42922	-1.53501	-1.09392
C	1.65177	1.43071	-1.94377
O	2.46291	1.59715	-2.76615

$vibrational spectrum

# mode	symmetry	wave number cm**(-1)	IR intensity km/mol	selection rules IR	RAMAN
1		0.00	0.00000	-	-
2		0.00	0.00000	-	-
3		0.00	0.00000	-	-
4		0.00	0.00000	-	-
5		0.00	0.00000	-	-
6		0.00	0.00000	-	-
7	a	15.82	0.03519	YES	YES
8	a	23.00	0.22563	YES	YES
9	a	27.88	0.41776	YES	YES
10	a	33.18	0.28278	YES	YES
11	a	36.95	0.00657	YES	YES
12	a	51.08	0.11682	YES	YES
13	a	61.76	0.23887	YES	YES
14	a	68.22	0.20227	YES	YES
15	a	79.15	0.03369	YES	YES
16	a	88.73	0.35772	YES	YES
17	a	97.17	0.18178	YES	YES
18	a	101.80	0.34931	YES	YES
19	a	109.97	0.68153	YES	YES
20	a	113.52	0.48190	YES	YES
21	a	121.90	0.52662	YES	YES
22	a	129.47	0.35119	YES	YES
23	a	137.53	0.12192	YES	YES
24	a	146.80	2.99958	YES	YES
25	a	156.96	0.73343	YES	YES
26	a	172.92	0.25159	YES	YES
27	a	196.27	3.02614	YES	YES
28	a	198.58	2.21502	YES	YES
29	a	220.79	0.52067	YES	YES
30	a	235.83	1.14163	YES	YES
31	a	245.22	0.31159	YES	YES
32	a	284.88	0.40943	YES	YES
33	a	301.62	0.84880	YES	YES
34	a	320.28	22.97572	YES	YES
35	a	350.52	1.02195	YES	YES
36	a	372.12	1.28637	YES	YES
37	a	399.00	1.27625	YES	YES
38	a	402.75	0.45898	YES	YES
39	a	404.75	3.50659	YES	YES
40	a	432.33	0.96303	YES	YES
41	a	440.62	2.09758	YES	YES
42	a	462.99	3.80011	YES	YES
43	a	469.93	0.57574	YES	YES
44	a	472.91	4.84551	YES	YES
45	a	486.06	3.28811	YES	YES
46	a	492.19	27.96598	YES	YES
47	a	495.50	22.18241	YES	YES
48	a	512.97	4.46311	YES	YES
49	a	515.69	9.32231	YES	YES
50	a	527.11	6.29771	YES	YES

Complex 5b-iso

SCF Energy (au) (RI)BP86/SV(P) -2277.5279704610
SCF Energy (au) PBE0/def2-TZVPP -2277.079674398
SCF Energy (au) PBE0/def2-TZVPP -2277.0899479355 (Et2O Correction)
Zero Point Energy (au) 0.2917488
Chemical potential (kJ mol-1) 627.54
Dispersion correction (au) PBE0/def2-TZVPP -0.06221297

xyz coordinates

41

C	0.11506	-1.04987	3.18138
C	-1.68297	0.45180	2.43257
C	0.31260	0.57413	0.27310
C	-1.76216	-2.15299	1.64126
C	0.94879	-1.80162	0.31960
C	1.76034	-2.86925	0.88414
C	1.38624	-4.23726	-1.10464
C	0.60048	-3.26183	-1.68617
H	0.14836	-3.44426	-2.67454
C	0.30797	-2.04059	-0.99567
C	-0.57325	-1.05158	-1.44162
C	-1.26645	-0.97664	-2.68050
H	-0.99258	-1.70203	-3.46262
C	-2.28428	-0.06818	-2.89947
H	-2.81642	-0.03737	-3.86213
C	-2.65410	0.82982	-1.83348
H	-3.51039	1.51282	-1.94626
C	-1.94850	0.86450	-0.66902
H	-2.16299	1.56174	0.15142
Mn	-0.62872	-0.72563	1.59418
N	-0.83937	-0.01135	-0.42963
O	0.63751	-1.28326	4.20177
O	-2.37178	1.20690	3.00169
C	1.22756	-0.42029	0.67577
O	-2.49985	-3.05191	1.67499
C	1.95914	-4.04590	0.20352
H	2.14796	-0.13225	1.20634
C	0.56900	2.03534	0.29445
C	1.16792	4.79965	0.31461
C	1.26649	4.04592	1.49866
C	0.96133	2.67743	1.49129
C	0.47767	2.79885	-0.89348
C	0.77121	4.17235	-0.87830
H	1.39778	5.87775	0.32383
H	1.56998	4.53125	2.44082
H	1.00509	2.09287	2.42499
H	0.19284	2.30994	-1.83878
H	0.69645	4.75463	-1.81160
H	1.56699	-5.18307	-1.64113
H	2.57072	-4.84681	0.64980
H	2.22836	-2.70377	1.86882

$vibrational spectrum

# mode	symmetry	wave number	IR intensity	selection rules	
#		cm**(-1)	km/mol	IR	RAMAN
1		0.00	0.00000	-	-
2		0.00	0.00000	-	-
3		0.00	0.00000	-	-
4		0.00	0.00000	-	-
5		0.00	0.00000	-	-
6		0.00	0.00000	-	-
7	a	42.29	0.06425	YES	YES
8	a	43.48	0.33674	YES	YES
9	a	46.29	0.13526	YES	YES
10	a	60.05	0.09926	YES	YES
11	a	62.05	0.07407	YES	YES
12	a	78.97	0.03583	YES	YES
13	a	85.71	0.13077	YES	YES
14	a	91.86	0.32603	YES	YES
15	a	103.36	0.53113	YES	YES
16	a	110.74	0.83272	YES	YES
17	a	126.78	1.10937	YES	YES
18	a	137.77	0.93380	YES	YES
19	a	156.55	1.89229	YES	YES
20	a	180.23	0.61820	YES	YES
21	a	217.19	0.67051	YES	YES
22	a	233.50	0.58195	YES	YES
23	a	248.73	0.13804	YES	YES
24	a	275.88	0.68066	YES	YES
25	a	286.15	2.56865	YES	YES
26	a	381.92	0.29874	YES	YES
27	a	398.21	1.38131	YES	YES
28	a	403.64	4.93022	YES	YES
29	a	407.23	1.25098	YES	YES
30	a	417.28	6.92016	YES	YES
31	a	428.34	4.42897	YES	YES
32	a	457.59	3.33767	YES	YES
33	a	458.71	5.10217	YES	YES
34	a	471.10	2.94826	YES	YES
35	a	479.48	3.18296	YES	YES
36	a	487.64	7.41628	YES	YES
37	a	491.33	3.65691	YES	YES
38	a	507.64	5.88200	YES	YES
39	a	522.66	25.16936	YES	YES
40	a	539.35	8.21135	YES	YES
41	a	555.45	3.25448	YES	YES
42	a	560.12	8.86594	YES	YES
43	a	586.56	25.68173	YES	YES
44	a	599.04	8.41344	YES	YES
45	a	603.64	7.86430	YES	YES
46	a	620.56	13.89848	YES	YES
47	a	637.99	50.84300	YES	YES
48	a	648.56	25.86957	YES	YES
49	a	667.20	21.13953	YES	YES
50	a	674.67	28.95069	YES	YES

Complex 5b

SCF Energy (au) (RI)BP86/SV(P) -2277.5551094000
SCF Energy (au) PBE0/def2-TZVPP -2277.110830801
SCF Energy (au) PBE0/def2-TZVPP -2277.1249607333 (Et2O Correction)
Zero Point Energy (au) 0.2929691
Chemical potential (kJ mol-1) 631.14
Dispersion correction (au) PBE0/def2-TZVPP -0.06224613

xyz coordinates

41

C	2.06993	1.20710	1.74734
C	-0.24244	0.16899	2.59891
C	0.11731	0.57227	-0.31161
C	2.09491	-1.16547	2.53138
C	1.33899	-1.54057	-0.25526
C	2.43327	-2.47350	-0.43277
C	1.24124	-4.13285	0.91873
C	0.13474	-3.32223	1.02191
H	-0.74467	-3.66412	1.59315
C	0.09940	-2.00867	0.40338
C	-1.16322	-1.48090	-0.11384
C	-2.36380	-2.20975	-0.23855
H	-2.36729	-3.26400	0.07038
C	-3.52140	-1.60810	-0.74796
H	-4.46288	-2.17793	-0.80315
C	-3.45827	-0.28171	-1.20513
H	-4.33388	0.23251	-1.62824
C	-2.24375	0.40078	-1.11581
H	-2.11669	1.43743	-1.45697
Mn	1.05043	-0.21457	1.41487
N	-1.14634	-0.16988	-0.55244
O	2.73776	2.14313	1.96705
O	-1.10199	0.45775	3.34078
C	1.33277	-0.17514	-0.64773
O	2.76411	-1.79779	3.25203
C	2.38315	-3.72189	0.13929
H	2.18805	0.28620	-1.16490
C	0.03785	2.03684	-0.62851
C	-0.11048	4.80697	-1.22938
C	0.49732	3.92083	-2.13356
C	0.56291	2.54798	-1.83828
C	-0.56878	2.94366	0.27290
C	-0.64745	4.31202	-0.02682
H	-0.16074	5.88460	-1.45737
H	0.92336	4.29833	-3.07808
H	1.02901	1.85883	-2.56214
H	-0.96767	2.56334	1.22702
H	-1.11534	5.00218	0.69459
H	1.25467	-5.11220	1.42328
H	3.23231	-4.41473	0.02100
H	3.31358	-2.14574	-1.00947

$vibrational spectrum

# mode	symmetry	wave number cm**(-1)	IR intensity km/mol	selection rules IR	RAMAN
1		0.00	0.00000	-	-
2		0.00	0.00000	-	-
3		0.00	0.00000	-	-
4		0.00	0.00000	-	-
5		0.00	0.00000	-	-
6		0.00	0.00000	-	-
7	a	28.13	0.60216	YES	YES
8	a	39.25	0.01045	YES	YES
9	a	52.87	1.08833	YES	YES
10	a	59.61	0.22659	YES	YES
11	a	72.98	0.24207	YES	YES
12	a	84.65	0.29468	YES	YES
13	a	85.94	0.94284	YES	YES
14	a	92.28	0.94778	YES	YES
15	a	99.48	1.10366	YES	YES
16	a	105.74	0.41481	YES	YES
17	a	116.67	0.82436	YES	YES
18	a	151.02	2.06371	YES	YES
19	a	167.14	0.74529	YES	YES
20	a	198.60	1.61552	YES	YES
21	a	225.88	0.81183	YES	YES
22	a	246.96	0.85082	YES	YES
23	a	267.11	0.62271	YES	YES
24	a	267.82	0.90253	YES	YES
25	a	317.37	2.20862	YES	YES
26	a	369.31	2.80034	YES	YES
27	a	404.98	0.02501	YES	YES
28	a	410.56	0.31641	YES	YES
29	a	424.84	2.74180	YES	YES
30	a	435.90	1.17912	YES	YES
31	a	451.00	0.31561	YES	YES
32	a	464.12	4.68165	YES	YES
33	a	472.09	6.39151	YES	YES
34	a	482.43	6.75717	YES	YES
35	a	485.09	0.60171	YES	YES
36	a	494.14	3.06781	YES	YES
37	a	509.57	3.92698	YES	YES
38	a	512.72	1.49118	YES	YES
39	a	523.18	4.62750	YES	YES
40	a	536.23	8.31751	YES	YES
41	a	561.24	3.02389	YES	YES
42	a	577.86	4.73094	YES	YES
43	a	603.79	20.75221	YES	YES
44	a	610.60	41.90090	YES	YES
45	a	616.00	34.23985	YES	YES
46	a	634.28	1.44896	YES	YES
47	a	641.16	15.27507	YES	YES
48	a	657.60	35.51284	YES	YES
49	a	672.08	63.58324	YES	YES
50	a	679.70	3.30883	YES	YES

Complex TSIVPh-VPh

SCF Energy (au) (RI)BP86/SV(P) -2585.6969763850
SCF Energy (au) PBE0/def2-TZVPP -2585.208683995
SCF Energy (au) PBE0/def2-TZVPP -2585.2226422105 (Et2O Correction)
Zero Point Energy (au) 0.3967098
Chemical potential (kJ mol-1) 871.84
Dispersion correction (au) PBE0/def2-TZVPP -0.08313758

xyz coordinates

55

Mn	0.16710	1.92451	-0.18383
C	1.19924	2.88928	0.94856
C	-0.86580	3.30158	-0.74661
O	-1.48952	4.20367	-1.14190
O	1.88844	3.47104	1.68370
C	-1.64071	0.45829	2.07144
C	-1.65156	2.78578	2.04951
C	-2.53345	2.87482	3.12716
C	-3.00933	1.68679	3.69785
C	-2.55371	0.48305	3.16015
C	-1.22362	-0.90306	1.62627
C	-1.41377	-1.40056	0.30574
C	-1.28765	-2.79843	0.09329
C	-0.90426	-3.67517	1.11268
C	-0.65000	-3.16937	2.40063
C	-0.83121	-1.80386	2.64812
H	-1.27237	3.71018	1.59385
H	-2.83768	3.86612	3.49717
H	-3.72372	1.69625	4.53721
H	-2.91665	-0.47619	3.55753
H	-1.47420	-3.18953	-0.92100
H	-0.79674	-4.75205	0.90250
H	-0.33124	-3.83998	3.21538
H	-0.65878	-1.41134	3.66358
N	-1.20456	1.62078	1.50478
C	-1.40067	1.00756	-2.69710
C	-0.45921	0.92041	-3.75020
C	-0.79978	1.31271	-5.05401
C	-2.08497	1.80891	-5.33539
C	-3.02952	1.90133	-4.29960
C	-2.69258	1.50240	-2.99565
H	0.54834	0.52241	-3.54748
H	-0.05099	1.22704	-5.85923
H	-2.34819	2.12353	-6.35884
H	-4.04125	2.28967	-4.50582
H	-3.43633	1.58270	-2.18542
C	-1.07881	0.54467	-1.31773
C	-1.71400	-0.58701	-0.88549
H	-2.40244	-1.07948	-1.60409
C	3.28887	-1.44826	0.63280
C	3.09019	-2.61625	1.41833
C	4.14238	-3.51524	1.63729
C	5.41309	-3.27279	1.08439
C	5.62489	-2.12064	0.30527
C	4.57823	-1.21745	0.07800
H	2.09282	-2.80316	1.84623
H	3.97040	-4.41770	2.24773
H	6.23857	-3.98249	1.26030
H	6.61842	-1.92456	-0.13136
H	4.74175	-0.31521	-0.53251
C	2.22826	-0.52204	0.40131

```
C         1.31559        0.30239        0.21237
H         0.26400        0.28858       -0.64737
C         1.31042        2.31808       -1.50290
O         2.08825        2.61727       -2.31760

$vibrational spectrum
#  mode     symmetry     wave number   IR intensity    selection rules
#                         cm**(-1)        km/mol          IR     RAMAN
     1         a          -818.71         0.00000        YES     YES
     2                       0.00         0.00000         -       -
     3                       0.00         0.00000         -       -
     4                       0.00         0.00000         -       -
     5                       0.00         0.00000         -       -
     6                       0.00         0.00000         -       -
     7                       0.00         0.00000         -       -
     8         a            18.30         0.19721        YES     YES
     9         a            21.28         0.01915        YES     YES
    10         a            26.40         0.75626        YES     YES
    11         a            33.40         0.19587        YES     YES
    12         a            42.15         0.09230        YES     YES
    13         a            50.64         0.08912        YES     YES
    14         a            57.19         0.13653        YES     YES
    15         a            67.34         0.68076        YES     YES
    16         a            75.41         0.20046        YES     YES
    17         a            84.28         0.13377        YES     YES
    18         a            91.22         0.17735        YES     YES
    19         a            97.03         0.68962        YES     YES
    20         a           101.39         0.27732        YES     YES
    21         a           106.48         0.31659        YES     YES
    22         a           115.84         1.61207        YES     YES
    23         a           117.76         0.45182        YES     YES
    24         a           126.50         0.19292        YES     YES
    25         a           144.88         0.28042        YES     YES
    26         a           168.61         0.87078        YES     YES
    27         a           187.86         7.01953        YES     YES
    28         a           190.45         4.95554        YES     YES
    29         a           216.18         1.64198        YES     YES
    30         a           218.75         0.32496        YES     YES
    31         a           244.43         0.22518        YES     YES
    32         a           266.79         8.56109        YES     YES
    33         a           282.94         0.69383        YES     YES
    34         a           302.88        10.82200        YES     YES
    35         a           321.54         0.78416        YES     YES
    36         a           359.86        12.20792        YES     YES
    37         a           371.06         8.14164        YES     YES
    38         a           400.89         0.86041        YES     YES
    39         a           402.33         0.27258        YES     YES
    40         a           404.56         5.61992        YES     YES
    41         a           437.79         5.59958        YES     YES
    42         a           446.77         1.14749        YES     YES
    43         a           470.12         3.85869        YES     YES
    44         a           476.75         6.23337        YES     YES
    45         a           480.72         2.71564        YES     YES
    46         a           489.31         4.01073        YES     YES
    47         a           493.95         3.24537        YES     YES
    48         a           508.99         0.94088        YES     YES
    49         a           516.54         7.40170        YES     YES
    50         a           520.12         4.68768        YES     YES
```

Complex VPh

SCF Energy (au) (RI)BP86/SV(P) -2585.7461640620
SCF Energy (au) PBE0/def2-TZVPP -2585.259938642
SCF Energy (au) PBE0/def2-TZVPP -2585.2761329115 (Et2O Correction)
Zero Point Energy (au) 0.4022033
Chemical potential (kJ mol-1) 882.64
Dispersion correction (au) PBE0/def2-TZVPP -0.08241356

xyz coordinates

55

Mn	0.01290	1.68683	-0.18082
C	1.07773	2.74322	0.79788
C	-1.09994	3.03775	-0.68500
O	-1.74213	3.96679	-0.98646
O	1.79267	3.41150	1.42705
C	-1.59111	0.22040	2.19103
C	-1.50397	2.55010	2.32144
C	-2.23146	2.58549	3.51418
C	-2.67742	1.37252	4.05833
C	-2.35916	0.19301	3.38246
C	-1.26410	-1.05728	1.51843
C	-1.20652	-1.14239	0.10001
C	-0.91513	-2.39012	-0.49185
C	-0.64950	-3.52508	0.28832
C	-0.68644	-3.43345	1.68847
C	-1.00154	-2.20819	2.29265
H	-1.16130	3.49053	1.86669
H	-2.44609	3.55431	3.99084
H	-3.27457	1.34614	4.98445
H	-2.72690	-0.77506	3.75298
H	-0.91346	-2.47140	-1.59043
H	-0.41325	-4.48411	-0.20099
H	-0.46110	-4.31260	2.31324
H	-0.99016	-2.13271	3.39171
N	-1.17676	1.41198	1.66039
C	-1.38825	0.96046	-3.14558
C	-0.60847	0.99124	-4.32835
C	-1.08390	1.61089	-5.49340
C	-2.35296	2.21395	-5.50239
C	-3.14285	2.18750	-4.33784
C	-2.66843	1.56709	-3.17483
H	0.38918	0.52262	-4.32711
H	-0.45789	1.62311	-6.40068
H	-2.73005	2.70154	-6.41653
H	-4.14227	2.65288	-4.33802
H	-3.30830	1.54777	-2.27866
C	-0.85671	0.24759	-1.96547
C	-1.49886	0.03593	-0.74879
H	-2.45972	0.54556	-0.55319
C	3.27170	-1.37026	1.07850
C	3.60639	-1.57904	2.44576
C	4.62996	-2.46435	2.81037
C	5.34928	-3.16782	1.82684
C	5.03336	-2.97228	0.46974
C	4.01186	-2.08785	0.09764
H	3.04707	-1.02648	3.21777
H	4.87229	-2.60647	3.87739
H	6.15502	-3.86291	2.11619
H	5.59351	-3.51556	-0.31031
H	3.76935	-1.93376	-0.96612
C	2.23249	-0.46586	0.70116

C	1.30062	0.29302	0.37774
H	0.02981	-0.37955	-2.16589
C	1.12535	1.95316	-1.55592
O	1.89013	2.13967	-2.41503

$vibrational spectrum

# mode	symmetry	wave number cm**(-1)	IR intensity km/mol	selection rules IR	selection rules RAMAN
1		0.00	0.00000	-	-
2		0.00	0.00000	-	-
3		0.00	0.00000	-	-
4		0.00	0.00000	-	-
5		0.00	0.00000	-	-
6		0.00	0.00000	-	-
7	a	13.01	0.00385	YES	YES
8	a	15.57	0.01558	YES	YES
9	a	20.84	0.13766	YES	YES
10	a	28.82	0.57255	YES	YES
11	a	42.77	0.09474	YES	YES
12	a	48.98	0.12729	YES	YES
13	a	51.53	0.52488	YES	YES
14	a	62.95	1.22310	YES	YES
15	a	74.97	0.67223	YES	YES
16	a	83.63	0.42445	YES	YES
17	a	90.59	1.05657	YES	YES
18	a	94.05	0.73019	YES	YES
19	a	101.70	1.50337	YES	YES
20	a	107.04	1.31684	YES	YES
21	a	107.72	0.45034	YES	YES
22	a	121.72	0.83483	YES	YES
23	a	128.27	0.13181	YES	YES
24	a	152.60	4.62867	YES	YES
25	a	162.22	0.63572	YES	YES
26	a	174.48	0.17281	YES	YES
27	a	176.69	1.87810	YES	YES
28	a	190.58	3.13033	YES	YES
29	a	217.64	0.43701	YES	YES
30	a	252.20	0.67843	YES	YES
31	a	275.65	0.17920	YES	YES
32	a	283.28	2.77329	YES	YES
33	a	298.55	0.51402	YES	YES
34	a	315.04	1.85625	YES	YES
35	a	346.58	4.69166	YES	YES
36	a	384.33	1.57518	YES	YES
37	a	391.92	6.79024	YES	YES
38	a	401.50	0.53221	YES	YES
39	a	402.85	0.00541	YES	YES
40	a	406.54	4.42489	YES	YES
41	a	444.40	2.34286	YES	YES
42	a	459.54	2.48149	YES	YES
43	a	466.74	1.77026	YES	YES
44	a	479.50	0.98956	YES	YES
45	a	486.58	3.63794	YES	YES
46	a	493.67	5.36757	YES	YES
47	a	505.44	16.01629	YES	YES
48	a	508.92	2.29273	YES	YES
49	a	510.87	0.67256	YES	YES
50	a	519.02	14.69989	YES	YES

Complex TSIVPh-VIPh

SCF Energy (au) (RI)BP86/SV(P) -2585.7027841150
SCF Energy (au) PBE0/def2-TZVPP -2585.208438866
SCF Energy (au) PBE0/def2-TZVPP -2585.2219306890 (Et2O Correction)
Zero Point Energy (au) 0.4003756
Chemical potential (kJ mol-1) 886.98
Dispersion correction (au) PBE0/def2-TZVPP -0.08728449

xyz coordinates

55

C	1.05117	-0.39583	-1.08134
C	-0.83611	0.36995	-1.54668
Mn	0.50454	1.51411	-0.38597
C	1.54887	2.36855	0.83236
C	-0.52635	2.93858	-0.81577
O	-1.15669	3.87217	-1.11770
O	2.23525	2.92997	1.58816
C	-1.37436	0.02641	1.91843
C	-1.24027	2.34706	2.00745
C	-2.00600	2.43169	3.17215
C	-2.48294	1.24184	3.73933
C	-2.16599	0.04297	3.09986
C	-1.15346	-1.31246	1.30759
C	-1.51072	-1.60893	-0.04115
C	-1.67474	-2.97697	-0.39389
C	-1.38380	-4.01821	0.49184
C	-0.92726	-3.71827	1.78869
C	-0.84622	-2.38081	2.18885
H	-0.85510	3.27096	1.55415
H	-2.21850	3.41787	3.61319
H	-3.10340	1.24765	4.65059
H	-2.55834	-0.91083	3.48207
H	-2.00642	-3.21168	-1.41971
H	-1.49994	-5.06558	0.16737
H	-0.65858	-4.52327	2.49180
H	-0.53220	-2.14416	3.21884
N	-0.91331	1.18812	1.37829
C	-1.09118	0.88063	-2.92954
C	-0.11402	0.86321	-3.95447
C	-0.41460	1.30929	-5.25087
C	-1.69481	1.80112	-5.55827
C	-2.67333	1.83701	-4.55123
C	-2.37721	1.37862	-3.25740
H	0.90072	0.49063	-3.74711
H	0.36621	1.27259	-6.02881
H	-1.92452	2.16028	-6.57476
H	-3.68032	2.23003	-4.77108
H	-3.14960	1.42190	-2.47178
C	-1.66750	-0.65237	-1.14194
H	-2.40592	-0.98650	-1.89985
C	2.58059	-0.74506	1.00974
C	2.60464	-0.37782	2.37870
C	3.61368	-0.84655	3.23355
C	4.62300	-1.69251	2.74271
C	4.61254	-2.07216	1.38727
C	3.60550	-1.60726	0.53181
H	1.81897	0.28758	2.76856
H	3.61508	-0.54285	4.29372
H	5.42110	-2.05237	3.41312
H	5.40412	-2.73034	0.99128
H	3.60762	-1.89494	-0.53205

```
C          1.56074       -0.26517        0.09723
H          1.10863       -0.95569       -2.01847
C          1.63505        2.07192       -1.63475
O          2.40568        2.47589       -2.41414

$vibrational spectrum
#  mode     symmetry     wave number   IR intensity    selection rules
#                         cm**(-1)        km/mol          IR     RAMAN
     1        a           -155.74          0.00000        YES     YES
     2                       0.00          0.00000         -       -
     3                       0.00          0.00000         -       -
     4                       0.00          0.00000         -       -
     5                       0.00          0.00000         -       -
     6                       0.00          0.00000         -       -
     7                       0.00          0.00000         -       -
     8        a             19.69          0.07473        YES     YES
     9        a             27.79          0.52798        YES     YES
    10        a             37.27          0.03193        YES     YES
    11        a             40.00          0.06882        YES     YES
    12        a             44.26          0.08914        YES     YES
    13        a             53.32          0.29347        YES     YES
    14        a             63.95          0.72767        YES     YES
    15        a             73.47          0.08001        YES     YES
    16        a             82.49          0.11039        YES     YES
    17        a             86.70          0.09430        YES     YES
    18        a             94.39          0.95455        YES     YES
    19        a            104.99          0.16620        YES     YES
    20        a            113.97          0.73587        YES     YES
    21        a            116.17          0.52501        YES     YES
    22        a            121.31          0.01179        YES     YES
    23        a            129.67          0.55420        YES     YES
    24        a            141.87          0.24634        YES     YES
    25        a            151.04          0.15456        YES     YES
    26        a            170.31          0.08500        YES     YES
    27        a            194.33          0.77608        YES     YES
    28        a            210.31          1.64438        YES     YES
    29        a            223.37          0.11023        YES     YES
    30        a            238.72          0.65745        YES     YES
    31        a            256.79          2.66190        YES     YES
    32        a            280.21          0.47172        YES     YES
    33        a            314.82          1.43156        YES     YES
    34        a            342.49          1.92713        YES     YES
    35        a            363.88          5.41031        YES     YES
    36        a            374.65          0.95676        YES     YES
    37        a            402.82          1.32616        YES     YES
    38        a            405.30          1.32648        YES     YES
    39        a            411.72         17.79907        YES     YES
    40        a            422.67          5.15219        YES     YES
    41        a            437.51          3.41380        YES     YES
    42        a            447.35          5.81998        YES     YES
    43        a            460.78          1.55284        YES     YES
    44        a            471.66          1.90331        YES     YES
    45        a            476.03          1.85540        YES     YES
    46        a            487.76          1.99790        YES     YES
    47        a            500.12          5.00821        YES     YES
    48        a            505.14          7.21102        YES     YES
    49        a            518.06          2.95945        YES     YES
    50        a            527.61          2.09376        YES     YES
```

Complex VIPh

SCF Energy (au) (RI)BP86/SV(P) -2585.7857465200
SCF Energy (au) PBE0/def2-TZVPP -2585.296922049
SCF Energy (au) PBE0/def2-TZVPP -2585.3098695133 (Et2O Correction)
Zero Point Energy (au) 0.4044353
Chemical potential (kJ mol-1) 904.30
Dispersion correction (au) PBE0/def2-TZVPP -0.08920220

xyz coordinates

55

C	0.86772	-0.80798	-0.50904
C	-0.05378	-0.65775	-1.60620
Mn	0.79905	1.29968	-0.66890
C	1.55040	2.51694	0.42244
C	0.47296	2.32933	-2.10947
O	0.24684	2.97107	-3.05850
O	2.06613	3.30704	1.10944
C	-1.50434	1.11255	1.22772
C	-1.51315	3.14134	0.07372
C	-2.51911	3.66352	0.89111
C	-2.99863	2.87876	1.95323
C	-2.48846	1.58554	2.11501
C	-1.01680	-0.34728	1.18265
C	-1.90937	-0.86849	0.03315
C	-3.21115	-1.39878	0.35751
C	-3.51812	-1.81076	1.63186
C	-2.50457	-1.73708	2.66770
C	-1.31826	-1.09507	2.47124
H	-1.08596	3.74114	-0.74437
H	-2.90136	4.67834	0.70104
H	-3.76685	3.26801	2.64148
H	-2.83825	0.92106	2.91859
H	-3.92053	-1.56495	-0.47223
H	-4.49151	-2.27827	1.85136
H	-2.69529	-2.23345	3.63504
H	-0.56065	-1.07395	3.26898
N	-0.99637	1.89933	0.24412
C	0.35645	-0.93126	-3.01968
C	1.49488	-1.70860	-3.33976
C	1.84658	-1.96839	-4.67403
C	1.06675	-1.46322	-5.72737
C	-0.07066	-0.69292	-5.42840
C	-0.41859	-0.43142	-4.09521
H	2.11512	-2.13976	-2.53797
H	2.73844	-2.58019	-4.88953
H	1.34436	-1.66674	-6.77466
H	-0.68970	-0.28073	-6.24267
H	-1.29657	0.20060	-3.88264
C	-1.45912	-0.79145	-1.26686
H	-2.17196	-1.00381	-2.08244
C	1.50934	-0.55676	1.91196
C	1.58209	0.37254	2.97846
C	2.50582	0.21184	4.02164
C	3.38578	-0.88520	4.02827
C	3.32245	-1.82224	2.98452
C	2.39061	-1.66302	1.94534
H	0.91298	1.24883	2.98173
H	2.54647	0.95794	4.83272
H	4.11561	-1.00916	4.84531
H	3.99706	-2.69473	2.98097
H	2.33200	-2.42860	1.15448

```
C          0.51580      -0.39007       0.80006
H          1.86404      -1.23957      -0.68481
C          2.44405       1.02399      -1.31946
O          3.52928       0.89221      -1.72868

$vibrational spectrum
#  mode     symmetry     wave number   IR intensity    selection rules
#                         cm**(-1)        km/mol         IR     RAMAN
     1                        0.00         0.00000        -       -
     2                        0.00         0.00000        -       -
     3                        0.00         0.00000        -       -
     4                        0.00         0.00000        -       -
     5                        0.00         0.00000        -       -
     6                        0.00         0.00000        -       -
     7         a             30.48         0.24741       YES     YES
     8         a             37.78         0.64948       YES     YES
     9         a             41.26         1.02279       YES     YES
    10         a             51.02         0.09730       YES     YES
    11         a             55.61         0.29075       YES     YES
    12         a             60.36         0.02674       YES     YES
    13         a             69.13         0.03744       YES     YES
    14         a             77.20         0.51465       YES     YES
    15         a             86.43         0.16906       YES     YES
    16         a             89.25         0.49140       YES     YES
    17         a            100.28         0.27810       YES     YES
    18         a            102.41         0.38183       YES     YES
    19         a            108.91         0.06864       YES     YES
    20         a            122.59         0.23125       YES     YES
    21         a            142.37         0.53336       YES     YES
    22         a            164.44         1.55917       YES     YES
    23         a            173.35         3.76589       YES     YES
    24         a            190.12         0.29340       YES     YES
    25         a            200.17         0.29123       YES     YES
    26         a            212.23         1.39410       YES     YES
    27         a            224.37         0.29196       YES     YES
    28         a            247.83         0.69322       YES     YES
    29         a            251.91         0.21037       YES     YES
    30         a            269.98         0.83562       YES     YES
    31         a            293.13         0.44399       YES     YES
    32         a            311.59         1.85552       YES     YES
    33         a            350.63         0.03883       YES     YES
    34         a            388.55         1.44839       YES     YES
    35         a            403.10         0.40644       YES     YES
    36         a            407.36         0.04911       YES     YES
    37         a            412.50         9.12295       YES     YES
    38         a            425.63         3.69594       YES     YES
    39         a            438.08         0.64853       YES     YES
    40         a            442.00        10.36629       YES     YES
    41         a            470.40         2.60240       YES     YES
    42         a            477.03         2.36729       YES     YES
    43         a            481.39         0.46121       YES     YES
    44         a            483.07         0.39993       YES     YES
    45         a            497.52         4.18563       YES     YES
    46         a            503.18         5.93483       YES     YES
    47         a            512.56         2.62473       YES     YES
    48         a            529.85        15.36549       YES     YES
    49         a            538.23        11.38191       YES     YES
    50         a            548.73        11.83674       YES     YES
```

Complex VIIaPh

SCF Energy (au) (RI)BP86/SV(P) -2585.7615008410
SCF Energy (au) PBE0/def2-TZVPP -2585.277080570
SCF Energy (au) PBE0/def2-TZVPP -2585.2923625444 (Et2O Correction)
Zero Point Energy (au) 0.4036996
Chemical potential (kJ mol-1) 896.52
Dispersion correction (au) PBE0/def2-TZVPP -0.08177741

xyz coordinates

55

C	-0.47827	0.93962	-0.52101
C	0.96113	0.79985	-0.68191
Mn	2.38794	-0.97133	-3.89648
C	3.89262	-1.87765	-4.25169
C	1.63074	-1.71980	-5.33818
O	1.13216	-2.19124	-6.28072
O	4.87930	-2.45480	-4.48123
C	-0.38134	-0.95594	2.24626
C	-0.05498	-3.05903	1.34240
C	-0.34282	-3.70123	2.56084
C	-0.65910	-2.89721	3.66606
C	-0.67906	-1.50080	3.51180
C	-0.33743	0.60284	1.97476
C	1.16248	0.90782	1.78289
C	1.94626	1.20672	2.95951
C	1.35381	1.61483	4.12938
C	-0.08564	1.80126	4.17798
C	-0.88893	1.37740	3.16372
H	0.20677	-3.64389	0.44087
H	-0.31567	-4.80052	2.63656
H	-0.88756	-3.35017	4.64581
H	-0.92131	-0.83823	4.35601
H	-1.97481	1.55822	3.20283
N	-0.07317	-1.72767	1.19348
C	1.55521	0.67100	-1.94138
C	0.82674	0.55549	-3.20888
C	1.42710	0.88616	-4.44756
C	2.84721	1.01660	-4.56168
C	3.61708	0.73513	-3.38941
C	2.98055	0.40378	-2.16940
H	-0.25878	0.37508	-3.20354
H	0.80069	0.96292	-5.35093
H	3.32619	1.27957	-5.51546
H	4.71612	0.69169	-3.45860
H	3.62049	0.09756	-1.32807
C	1.73769	0.85353	0.53371
H	2.83488	0.95380	0.47167
C	-2.60527	1.04740	0.71827
C	-3.43762	0.19710	1.49050
C	-4.83476	0.32974	1.46456
C	-5.44248	1.31853	0.67151
C	-4.63427	2.17743	-0.09323
C	-3.23754	2.04570	-0.06604
H	-2.98488	-0.59914	2.10227
H	-5.45587	-0.35456	2.06673
H	-6.54007	1.42260	0.65411
H	-5.09521	2.96885	-0.70800
H	-2.61242	2.74716	-0.64288
C	-1.12640	0.89337	0.68243
H	-1.09318	1.07992	-1.42425
C	1.75311	-2.22984	-2.75763

```
O          1.35356       -3.05300       -2.04041
H          3.04652        1.18991        2.86673
H          1.96927        1.89307        5.00070
H         -0.52682        2.32439        5.04420

$vibrational spectrum
#  mode     symmetry     wave number   IR intensity    selection rules
#                         cm**(-1)        km/mol         IR     RAMAN
     1                      0.00         0.00000         -        -
     2                      0.00         0.00000         -        -
     3                      0.00         0.00000         -        -
     4                      0.00         0.00000         -        -
     5                      0.00         0.00000         -        -
     6                      0.00         0.00000         -        -
     7         a           20.63         0.54158        YES      YES
     8         a           27.04         0.55096        YES      YES
     9         a           32.88         0.21511        YES      YES
    10         a           40.38         0.80053        YES      YES
    11         a           47.49         1.16584        YES      YES
    12         a           54.81         0.40465        YES      YES
    13         a           56.93         0.12798        YES      YES
    14         a           60.93         0.73137        YES      YES
    15         a           81.54         0.50597        YES      YES
    16         a           89.26         0.32399        YES      YES
    17         a           89.64         1.38419        YES      YES
    18         a          106.38         0.49352        YES      YES
    19         a          110.61         0.63658        YES      YES
    20         a          125.17         1.99024        YES      YES
    21         a          128.83         0.11263        YES      YES
    22         a          142.27         2.32791        YES      YES
    23         a          167.33         0.09543        YES      YES
    24         a          191.67         0.74490        YES      YES
    25         a          206.13         4.35963        YES      YES
    26         a          213.24         4.05289        YES      YES
    27         a          228.43         0.38069        YES      YES
    28         a          245.10         2.35134        YES      YES
    29         a          260.59         2.94533        YES      YES
    30         a          280.53        16.83325        YES      YES
    31         a          298.67         8.07313        YES      YES
    32         a          321.90         1.55789        YES      YES
    33         a          341.74         5.48413        YES      YES
    34         a          368.07         1.68309        YES      YES
    35         a          379.10        14.73030        YES      YES
    36         a          401.03         1.94993        YES      YES
    37         a          407.11         0.37612        YES      YES
    38         a          416.51         0.05916        YES      YES
    39         a          435.60         3.47340        YES      YES
    40         a          445.69         5.51922        YES      YES
    41         a          461.44         8.47971        YES      YES
    42         a          479.35         4.69265        YES      YES
    43         a          481.90         4.31579        YES      YES
    44         a          482.58         6.77723        YES      YES
    45         a          485.61         1.78153        YES      YES
    46         a          492.39         3.14881        YES      YES
    47         a          499.08        29.40351        YES      YES
    48         a          516.16         6.54529        YES      YES
    49         a          523.41        73.34846        YES      YES
    50         a          536.76         8.46781        YES      YES
```

Complex VIIbPh

SCF Energy (au) (RI)BP86/SV(P) -2585.7601751930
SCF Energy (au) PBE0/def2-TZVPP -2585.275812042
SCF Energy (au) PBE0/def2-TZVPP -2585.2920412018 (Et2O Correction)
Zero Point Energy (au) 0.4035734
Chemical potential (kJ mol-1) 894.01
Dispersion correction (au) PBE0/def2-TZVPP -0.08100534

xyz coordinates

55

C	-0.52316	0.37309	-0.49675
C	0.91531	0.23318	-0.66424
Mn	2.60273	1.32290	-4.06640
C	1.98795	2.01737	-5.59950
C	4.25007	1.87652	-4.50481
O	5.32893	2.21841	-4.78445
O	1.57852	2.45386	-6.60000
C	-0.45197	-1.50391	2.30637
C	-0.22455	-3.63345	1.43825
C	-0.46485	-4.24306	2.68292
C	-0.70460	-3.40689	3.78366
C	-0.69944	-2.01479	3.59718
C	-0.38068	0.04651	2.00203
C	1.12397	0.31835	1.80209
C	1.91823	0.59622	2.97781
C	1.33818	1.03625	4.14232
C	-0.09474	1.27102	4.18646
C	-0.91067	0.85655	3.17850
H	-0.02981	-4.25062	0.53999
H	-0.46201	-5.34087	2.78202
H	-0.89459	-3.83209	4.78379
H	-0.88641	-1.32818	4.43591
H	-1.99054	1.07114	3.21212
N	-0.21496	-2.30628	1.25648
C	1.50591	0.12239	-1.92875
C	2.95054	0.07755	-2.18005
C	3.49042	-0.51294	-3.34768
C	2.66255	-0.78011	-4.48263
C	1.29563	-0.36849	-4.39242
C	0.79185	0.21849	-3.20651
H	3.65591	0.33690	-1.37639
H	4.57574	-0.69370	-3.41147
H	3.06587	-1.24464	-5.39342
H	0.64831	-0.43324	-5.28164
H	-0.24190	0.59354	-3.23187
C	1.69392	0.26563	0.55080
H	2.79373	0.32517	0.49237
C	-2.64680	0.48595	0.74880
C	-3.47715	-0.34209	1.54700
C	-4.87391	-0.20400	1.52876
C	-5.48363	0.76905	0.71808
C	-4.67756	1.60732	-0.07152
C	-3.28138	1.47009	-0.05194
H	-3.02381	-1.12444	2.17586
H	-5.49309	-0.87100	2.15197
H	-6.58081	0.87829	0.70747
H	-5.13953	2.38793	-0.69915
H	-2.65821	2.15875	-0.64594
C	-1.16838	0.32977	0.70879
H	-1.14534	0.49016	-1.39766
C	2.21921	2.80147	-3.09235

```
O          1.97052        3.75138       -2.46992
H          3.01747        0.54460        2.88592
H          1.96176        1.30618        5.01042
H         -0.51874        1.82281        5.04332

$vibrational spectrum
#  mode     symmetry     wave number   IR intensity    selection rules
#                         cm**(-1)        km/mol         IR     RAMAN
     1                        0.00          0.00000       -       -
     2                        0.00          0.00000       -       -
     3                        0.00          0.00000       -       -
     4                        0.00          0.00000       -       -
     5                        0.00          0.00000       -       -
     6                        0.00          0.00000       -       -
     7         a             18.53          0.12549      YES     YES
     8         a             19.79          0.47919      YES     YES
     9         a             32.50          0.08758      YES     YES
    10         a             36.46          0.31315      YES     YES
    11         a             38.24          1.64647      YES     YES
    12         a             51.41          0.97012      YES     YES
    13         a             57.59          0.31850      YES     YES
    14         a             58.67          0.02851      YES     YES
    15         a             82.33          0.10566      YES     YES
    16         a             88.16          1.01084      YES     YES
    17         a             89.76          1.02886      YES     YES
    18         a            106.90          0.90026      YES     YES
    19         a            112.54          0.01305      YES     YES
    20         a            122.22          0.17732      YES     YES
    21         a            130.81          0.89439      YES     YES
    22         a            148.85          2.67908      YES     YES
    23         a            165.18         13.08519      YES     YES
    24         a            171.00          0.15575      YES     YES
    25         a            208.91          1.06147      YES     YES
    26         a            224.27          4.15977      YES     YES
    27         a            229.19          0.22748      YES     YES
    28         a            249.63          2.39717      YES     YES
    29         a            257.78          0.70489      YES     YES
    30         a            274.45          9.51992      YES     YES
    31         a            290.00         19.33926      YES     YES
    32         a            322.59          2.62132      YES     YES
    33         a            338.81          1.01470      YES     YES
    34         a            366.14          3.10651      YES     YES
    35         a            380.68         11.47740      YES     YES
    36         a            399.96          1.90040      YES     YES
    37         a            407.09          0.05927      YES     YES
    38         a            416.92          0.06438      YES     YES
    39         a            436.24          5.06146      YES     YES
    40         a            444.65          2.66150      YES     YES
    41         a            461.85          7.71946      YES     YES
    42         a            474.86          6.02745      YES     YES
    43         a            482.41          3.75220      YES     YES
    44         a            484.61          1.95439      YES     YES
    45         a            485.37          0.60294      YES     YES
    46         a            493.04          5.45140      YES     YES
    47         a            496.73         42.73093      YES     YES
    48         a            515.13          6.45941      YES     YES
    49         a            522.72         63.16807      YES     YES
    50         a            537.08         15.03188      YES     YES
```

Complex 10Ph

SCF Energy (au) (RI)BP86/SV(P) -2585.7883711070
SCF Energy (au) PBE0/def2-TZVPP -2585.304991267
SCF Energy (au) PBE0/def2-TZVPP -2585.3189446206 (Et2O Correction)
Zero Point Energy (au) 0.4041721
Chemical potential (kJ mol-1) 899.32
Dispersion correction (au) PBE0/def2-TZVPP -0.08732026

xyz coordinates

55

C	-2.71354	-1.55858	1.22415
C	-1.15523	-1.56419	3.17191
C	-0.51833	-3.02322	1.07938
C	1.21475	-0.60823	1.34840
C	0.32753	0.35688	1.89957
H	0.48068	0.69151	2.93891
C	-0.80542	0.84474	1.16692
C	-1.03172	0.20596	-0.10557
H	-1.98046	0.40467	-0.63115
C	-0.12474	-0.73145	-0.67039
C	1.34516	-0.70436	-0.19030
C	2.09117	-1.98671	-0.55598
H	2.00312	-2.34873	-1.59114
C	2.90566	-2.63593	0.31578
C	2.20749	-1.25343	2.19484
C	2.16323	0.51625	-0.71438
C	1.59746	1.54840	-1.49253
H	0.53851	1.51474	-1.78831
C	2.40988	2.62132	-1.89636
H	1.98996	3.43426	-2.51232
C	3.75547	2.63999	-1.50338
H	4.42946	3.46238	-1.79324
C	4.22433	1.56722	-0.72257
C	-1.69180	1.92738	1.67267
C	-1.96921	2.08161	3.05194
H	-1.56721	1.35572	3.77730
C	-2.78691	3.12467	3.50881
H	-2.99742	3.21762	4.58707
C	-3.35057	4.03736	2.59867
H	-3.99766	4.85387	2.95917
C	-3.08478	3.89682	1.22707
H	-3.51594	4.60811	0.50321
C	-2.26160	2.85474	0.76983
H	-2.03923	2.77345	-0.30678
C	-0.51792	-1.36883	-1.96927
C	-1.76105	-2.02779	-2.12071
H	-2.41756	-2.15312	-1.24633
C	-2.16615	-2.54751	-3.35964
H	-3.13808	-3.06218	-3.43899
C	-1.33399	-2.42990	-4.48546
H	-1.64686	-2.84628	-5.45711
C	-0.09580	-1.77959	-4.35644
H	0.56802	-1.67219	-5.23043
C	0.30737	-1.25631	-3.11731
H	1.27272	-0.73007	-3.05315
Mn	-0.95208	-1.30114	1.41325
N	3.45901	0.53528	-0.34460
O	-3.86659	-1.71938	1.13002
O	-1.28662	-1.71678	4.32202
O	-0.29543	-4.14986	0.88970
C	3.03869	-2.21648	1.69919

```
H          5.27899        1.53917       -0.38702
H          2.22980       -0.97790        3.26356
H          3.46356       -3.52402       -0.02666
H          3.76791       -2.71998        2.35417

$vibrational spectrum
#  mode     symmetry     wave number   IR intensity    selection rules
#                         cm**(-1)        km/mol         IR     RAMAN
     1                       0.00         0.00000         -       -
     2                       0.00         0.00000         -       -
     3                       0.00         0.00000         -       -
     4                       0.00         0.00000         -       -
     5                       0.00         0.00000         -       -
     6                       0.00         0.00000         -       -
     7         a            25.11         0.06392        YES     YES
     8         a            36.87         0.30958        YES     YES
     9         a            40.38         0.03368        YES     YES
    10         a            42.53         0.18033        YES     YES
    11         a            47.36         0.44949        YES     YES
    12         a            51.31         0.19054        YES     YES
    13         a            65.05         0.18758        YES     YES
    14         a            66.47         0.36188        YES     YES
    15         a            71.20         0.34695        YES     YES
    16         a            87.63         0.31608        YES     YES
    17         a            94.72         0.50447        YES     YES
    18         a            96.79         1.21589        YES     YES
    19         a           105.41         0.46623        YES     YES
    20         a           114.27         0.44948        YES     YES
    21         a           117.39         0.14183        YES     YES
    22         a           141.10         1.00444        YES     YES
    23         a           157.30         2.38592        YES     YES
    24         a           184.63         3.66150        YES     YES
    25         a           196.19         1.66483        YES     YES
    26         a           206.95         0.35915        YES     YES
    27         a           211.82         1.66013        YES     YES
    28         a           233.18         1.45079        YES     YES
    29         a           243.14         0.65240        YES     YES
    30         a           268.71         1.35909        YES     YES
    31         a           294.64         6.08372        YES     YES
    32         a           325.48         0.41767        YES     YES
    33         a           336.52         1.07377        YES     YES
    34         a           382.37         0.57233        YES     YES
    35         a           396.91         4.90187        YES     YES
    36         a           399.21         2.77133        YES     YES
    37         a           403.10         0.47570        YES     YES
    38         a           407.44         0.12068        YES     YES
    39         a           423.77         1.76842        YES     YES
    40         a           444.64         8.56038        YES     YES
    41         a           453.74         0.40888        YES     YES
    42         a           461.68         1.61225        YES     YES
    43         a           475.29        11.73835        YES     YES
    44         a           488.51         0.49663        YES     YES
    45         a           499.37         2.50927        YES     YES
    46         a           504.54         0.59257        YES     YES
    47         a           523.30        15.82477        YES     YES
    48         a           524.57         4.21860        YES     YES
    49         a           528.50        16.33218        YES     YES
    50         a           538.62        11.41079        YES     YES
```

PhC=CH

SCF Energy (au) (RI)BP86/SV(P) -308.1698939972
SCF Energy (au) PBE0/def2-TZVPP -308.1232351678
SCF Energy (au) PBE0/def2-TZVPP -308.1288805520 (Et2O Correction)
Zero Point Energy (au) 0.1064946
Chemical potential (kJ mol-1) 199.25
Dispersion correction (au) PBE0/def2-TZVPP -0.01224568

xyz coordinates

14

C	0.06253	-0.51956	0.51746
C	1.20573	0.18505	0.05791
C	1.08074	1.17386	-0.92774
C	-0.17983	1.47832	-1.47222
C	-1.31941	0.78636	-1.02417
C	-1.20440	-0.20407	-0.03883
H	2.19157	-0.05609	0.48600
H	1.97749	1.71311	-1.27539
H	-0.27456	2.25635	-2.24760
H	-2.31009	1.02126	-1.44742
H	-2.09512	-0.74750	0.31411
C	0.18593	-1.53070	1.52457
C	0.29314	-2.39627	2.38625
H	0.38630	-3.16013	3.14705

$vibrational spectrum

# mode	symmetry	wave number	IR intensity	selection rules	
#		cm**(-1)	km/mol	IR	RAMAN
1		0.00	0.00000	-	-
2		0.00	0.00000	-	-
3		0.00	0.00000	-	-
4		0.00	0.00000	-	-
5		0.00	0.00000	-	-
6		0.00	0.00000	-	-
7	a	137.62	1.54239	YES	YES
8	a	152.55	1.03541	YES	YES
9	a	359.94	2.76692	YES	YES
10	a	399.86	0.00003	YES	YES
11	a	458.49	0.36532	YES	YES
12	a	517.31	2.56247	YES	YES
13	a	545.24	5.37218	YES	YES
14	a	599.90	35.42594	YES	YES
15	a	614.16	0.52393	YES	YES
16	a	644.63	35.19285	YES	YES
17	a	692.49	31.23483	YES	YES
18	a	758.17	2.37031	YES	YES
19	a	760.72	32.28818	YES	YES
20	a	831.08	0.00007	YES	YES
21	a	909.88	3.38795	YES	YES
22	a	957.19	0.00017	YES	YES
23	a	982.21	0.08346	YES	YES
24	a	983.86	0.16195	YES	YES
25	a	1025.10	3.71162	YES	YES
26	a	1071.38	5.62927	YES	YES
27	a	1142.08	0.00035	YES	YES
28	a	1158.70	0.00087	YES	YES
29	a	1204.65	0.89088	YES	YES
30	a	1290.24	0.08234	YES	YES
31	a	1356.94	0.05075	YES	YES
32	a	1438.07	4.75311	YES	YES
33	a	1482.44	13.36586	YES	YES

34	a	1581.64	1.09448	YES	YES
35	a	1614.48	1.64749	YES	YES
36	a	2144.36	3.12605	YES	YES
37	a	3091.78	0.38767	YES	YES
38	a	3100.56	5.07513	YES	YES
39	a	3110.97	15.11875	YES	YES
40	a	3118.68	16.60336	YES	YES
41	a	3122.26	3.97788	YES	YES
42	a	3386.07	81.84204	YES	YES

5. References

1. A. Klamt, G. Schuurmann, *J. Chem. Soc., Perkin Trans. 2*, 1993, 799.
2. (a) S. Grimme, J. Antony, S. Ehrlich, H. Krieg, *J. Chem. Phys.*, 2010, **132**; (b) S. Grimme, S. Ehrlich, L. Goerigk, *J.Comput. Chem.*, 2011, **32**, 1456
3. (a) P. Csaszar, P. Pulay, *J. Mol,Struct.*, 1984, **114**, 31; (b) R. Ahlrichs, M. Bar, M. Haser, H. Horn, C. Kolmel, *Chem. Phys. Lett.*, 1989, **162**, 165; (c) K. Eichkorn, O. Treutler, H. Ohm, M. Haser, R. Ahlrichs, *Chem. Phys. Lett.*, 1995, **240**, 283; (d) O. Treutler, R. Ahlrichs, *J. Chem. Phys.*, 1995, **102**, 346; (e) K. Eichkorn, F. Weigend, O. Treutler, R. Ahlrichs, *Theor. Chem. Acc.*, 1997, **97**, 119; (f) M. v. Arnim, R. Ahlrichs, *J. Chem. Phys.*, 1999, **111**, 9183; (g) P. Deglmann, F. Furche, *J. Chem. Phys.*, 2002, **117**, 9535; (h) P. Deglmann, F. Furche, R. Ahlrichs, *Chem. Phys. Lett.*, 2002, **362**, 511; (i) P. Deglmann, K. May, F. Furche, R. Ahlrichs, *Chem. Phys. Lett.*, 2004, **384**, 103.
